# DNA methylation signatures associated with bipolar disorder in peripheral blood improve prediction models

**DOI:** 10.1016/j.ebiom.2026.106284

**Published:** 2026-05-08

**Authors:** Markos Tesfaye, Anne-Kristin Stavrum, Kira D. Höffler, Kevin S. O'Connell, Friederike S. David, Melanie E. Garrett, Sonia Hesam-Shariati, Bronwyn J. Overs, Claudia Pisanu, Luana Spano, Oliver J. Watkeys, Antoine Weihs, Rafaella Ardau, Allison E. Ashley-Koch, Lavinia Athanasiu, Jean C. Beckham, Kyle J. Bourassa, Caterina Chillotti, Srdjan Djurovic, Ole K. Drange, Josef Frank, Anouar Khayachi, Nathan A. Kimbrel, Lourdes Martorell, Susanne Meinert, Ingrid Melle, Gunnar Morken, Pasquale Paribello, Marco Pinna, Gloria Roberts, Guy Rouleau, Peter R. Schofield, Esteban Sepúlveda, Giovanni Severino, Vidar M. Steen, Frederike Stein, Fabian Streit, VA Mid-Atlantic MIRECC Workgroup, Jean Beckham, Patrick Calhoun, Eric Dedert, Eric Elbogen, Hurley Robin, Jason Kilts, Nathan Kimbrel, Angela Kirby, Anna Magnante, Sarah Martindale, Christine Marx, Scott McDonald, Scott Moore, Victoria O'Connor, Rajendra Morey, Jennifer Naylor, Jared Rowland, Robert Shura, Cindy Swinkels, Ryan Wagner, Trine Vik Lagerberg, Martin Alda, Udo Dannlowski, Andreas J. Forstner, Janice M. Fullerton, Hans J. Grabe, Melissa J. Green, Tilo Kircher, Javier Labad, Mirko Manchia, Philip B. Mitchell, Jair C. Soares, Alessio Squassina, Alexander Teumer, Leonardo Tondo, Elisabet Vilella, Ole A. Andreassen, Boris Chaumette, Gabriel R. Fries, Stephanie Le Hellard

**Affiliations:** a Department of Clinical Sciences, University of Bergen, Bergen, Norway; b Department of Psychiatry and Behavioral Sciences, Institute for Genomics in Health, State University of New York Downstate Health Sciences University, Brooklyn, NY, USA; c Centre for Precision Psychiatry, Division of Mental Health and Addiction, Oslo University Hospital and Institute of Clinical Medicine, University of Oslo, Oslo, Norway; d Bergen Center for Brain Plasticity, Haukeland University Hospital, Bergen, Norway; e Department of Psychiatry, McLean Hospital, Harvard Medical School, Belmont, MA, USA; f Institute of Human Genetics, University of Bonn, School of Medicine & University Hospital Bonn, Bonn, Germany; g Department of Psychiatry and Psychotherapy, Philipps-University, Marburg, Germany; h Duke Molecular Physiology Institute, Duke University Medical Center, Durham, NC, USA; i Neuroscience Research Australia, Randwick, Sydney, NSW, Australia; j School of Psychology, University of New South Wales, Sydney, Australia; k Department of Biomedical Sciences, University of Cagliari, Cagliari, Italy; l Université Paris Cité, Institute of Psychiatry and Neuroscience of Paris (IPNP), INSERM, U1266, Paris, France; m School of Clinical Medicine, University of New South Wales, Australia; n German Center for Neurodegenerative Diseases (DZNE), Site Rostock/Greifswald, Greifswald, Germany; o Department of Psychiatry and Psychotherapy, University Medicine Greifswald, Greifswald, Germany; p Unit of Clinical Pharmacology, University Hospital Agency of Cagliari, Cagliari, Italy; q VA Mid-Atlantic Mental Illness Research Education and Clinical Center, Durham, NC, USA; r Durham VA Health Care System, Durham, NC, USA; s Department of Psychiatry and Behavioral Sciences, Duke University School of Medicine, Durham, NC, USA; t Department of Psychology, Georgetown University, Washington, DC, USA; u Department of Medical Genetics, Oslo University Hospital and University of Oslo, Oslo, Norway; v Department of Psychiatry, Sørlandet Hospital HF, Arendal/Kristiansand, Norway; w Department of Genetic Epidemiology in Psychiatry, Central Institute of Mental Health, Medical Faculty Mannheim, Heidelberg University, Mannheim, Germany; x Montreal Neurological Institute-Hospital, McGill University, Montréal, Canada; y Hospital Universitari Institut Pere Mata, Reus, Spain; z Institut d'Investigació Sanitària Pere Virgili-CERCA, Reus, Spain; aa Universitat Rovira i Virgili, Tarragona, Spain; ab CIBERSAM-Instituto de Salud Carlos III, Madrid, Spain; ac Institute for Translational Psychiatry, University of Münster, Münster, Germany; ad Institute for Translational Neuroscience, University of Münster, Münster, Germany; ae Section for Clinical Psychosis Research, Division of Mental Health and Addiction, Oslo University Hospital, Oslo, Norway; af Department of Adult Psychiatry, Institute of Clinical Medicine, University of Oslo, Oslo, Norway; ag Department of Psychiatry, St Olavs University Hospital, Trondheim, Norway; ah Faculty of Medicine and Health Sciences, Norwegian University of Science and Technology (NTNU), Trondheim, Norway; ai Unit of Psychiatry, Department of Medical Sciences and Public Health, University of Cagliari, Cagliari, Italy; aj Discipline of Psychiatry and Mental Health, School of Clinical Medicine, Faculty of Medicine & Health, University of New South Wales, Sydney, Australia; ak Dr. Einar Martens Research Group for Biological Psychiatry, Department of Medical Genetics, Haukeland University Hospital, Bergen, Norway; al Center for Mind, Brain and Behavior, University of Marburg, Marburg, Germany; am Hector Institute for Artificial Intelligence in Psychiatry, Heidelberg University, Mannheim, Germany; an Department for Psychiatry and Psychotherapy, Central Institute of Mental Health, Mannheim, Germany; ao German Center for Mental Health (DZPG), Mannheim - Heidelberg - Ulm, Germany; ap Division of Mental Health and Addiction, Department for Research and Innovation, Oslo University Hospital, Oslo, Norway; aq Department of Psychology, University of Oslo, Oslo, Norway; ar Department of Psychiatry, Dalhousie University, Halifax, Nova Scotia, Canada; as National Institute of Mental Health, Klecany, Czech Republic; at Department of Psychiatry, Medical School and University Medical Center OWL, Protestant Hospital of the Bethel Foundation, Bielefeld University, Germany; au German Center for Mental Health (DZPG), Site Jena Magdeburg Halle, Germany; av Center for Intervention and Research on Adaptive and Maladaptive Brain Circuits Underlying Mental Health (C-I-R-C), Site Jena Magdeburg Halle, Germany; aw Institute of Neuroscience and Medicine (INM-1), Research Center Jülich, Germany; ax School of Biomedical Sciences, Faculty of Medicine & Health, University of New South Wales, Kensington, Sydney, NSW, Australia; ay Department of Mental Health and Addictions, Consorci Sanitari del Maresme, Mataró, Spain; az Translational Neuroscience Research Unit, Universitat Autònoma de Barcelona, Spain; ba Department of Pharmacology, Dalhousie University, Halifax, Canada; bb Faillace Department of Psychiatry and Behavioral Sciences, McGovern Medical School, University of Texas Health Science Center at Houston, Houston, TX, USA; bc Neuroscience Graduate Program, UT MD Anderson Cancer Center UTHealth Graduate School, Houston, TX, USA; bd German Centre for Cardiovascular Research (DZHK), Partner Site Greifswald, Greifswald, Germany; be Lucio Bini Mood Disorder Center, Cagliari, Italy; bf Department of Psychiatry, Harvard Medical School, Boston, MA, USA; bg K.G. Jebsen Centre for Neurodevelopmental Disorders, University of Oslo, Oslo, Norway; bh GHU-Paris Psychiatrie et Neurosciences, Hôpital Sainte Anne, Paris, France; bi Department of Psychiatry, McGill University, Montreal, Canada; bj School of Behavioral Health Sciences, University of Texas Health Science Center at Houston, Houston, TX, USA

**Keywords:** Bipolar disorder, DNA methylation, Epigenetics, Polymethylation score, Polygenic score, Clinical prediction

## Abstract

**Background:**

Bipolar disorder (BD) is a major mood disorder influenced by both genetic and environmental factors. While DNA methylation from peripheral tissues can reflect both genetic and environmental influences and reveal insights into disease biology, it remains understudied in BD. DNA methylation signatures may complement polygenic scores (PGS) and hold potential as biomarkers. Here, we conducted the largest epigenome-wide association study (EWAS) of BD to date and evaluated the predictive value of polymethylation scores (PMS) in classifying case–control status.

**Methods:**

DNA methylation from peripheral blood of 1729 cases and 1747 controls, comprising twelve cohorts, was obtained. We performed meta-analyses for the total sample, male-only, and female-only analyses. Differentially methylated regions (DMRs) were identified using the comb-p method. Polymethylation scores for BD (BD-PMS) were tested for association with BD, and in combination with PGS.

**Findings:**

We identified 47 differentially methylated CpG positions (DMPs) in the total and four in the female-only analysis. Ninety, fourteen and six DMRs were identified in the total sample, female-only, and male-only analyses, respectively. Genes annotated to the top DMPs were enriched for immune activation and phosphorylation pathways. DMRs were annotated to genes relevant to neurotransmission, including *GABBR1* and *CACNA2D4*. BD-PMS explained 2% of the variance in BD case–control status, and improved the variance explained from 7.9 to 8.5% when combined with PGS. For bipolar I disorder, BD-PMS explained 4.9% of the variance, and improved the variance explained by PGS from 15.9 to 18.5%. Association of BD with PMS for schizophrenia and major depression suggests pleiotropic epigenetic effects.

**Interpretation:**

DNA methylation signatures of BD are detectable in blood using adequately powered data and may reveal novel BD biology that is not captured by genetic studies. PMS from large cohorts have the potential to facilitate the development of prediction tools to aid clinical decision-making.

**Funding:**

This investigation was primarily funded by the Research Council of Norway (RCN #250299, #273446, #223273) and the University of Bergen. A complete list of funding organisations is provided in the Acknowledgements.

**Research in context**

**Evidence before this study**

DNA methylation changes, influenced by genetic or environmental factors, have been associated with bipolar disorder (BD) in candidate gene studies. However, whether epigenome-wide association studies (EWASs) using peripheral blood cells can be leveraged for classifying BD is not known. To assess the current state of research, we searched PubMed and Google Scholar (October 31, 2025), using terms: [[“epigenetic∗” OR “EWAS” OR “epigenome-wide” OR “methylation”] AND [“bipolar disorder” OR “bipolar I disorder” OR “bipolar II disorder”]], restricting the search to human studies and articles published in English.

Previous epigenetic studies in BD have been limited to relatively small samples, typically comprising at most a few hundred cases and controls. Most investigations have focused on selected candidate genes or global DNA methylation differences, with inconsistent replication of findings. Several of these studies have reported differential DNA methylation in individuals with BD compared with controls. One study examined the association between schizophrenia polymethylation scores (PMS) and BD case–control status in a small sample but found no association. More recently, methylation levels at seven DMRs were reported to predict lithium treatment response in patients with BD. To date, there are no EWAS meta-analyses involving multiple populations, nor analyses that incorporated sex-informed approaches to BD. No studies have evaluated whether PMS can improve the classification of BD cases from controls when combined with polygenic scores.

**Added value of this study**

The current study leveraged a dataset of 3476 individuals from seven countries and explored genome-wide DNA methylation variability. We identified 47 CpGs associated with BD diagnosis in the total sample analysis and four CpGs specific to females. Genes annotated to the top CpGs showed enrichment for immune-related pathways and phosphorylation processes. PMS derived from this study were significantly associated with BD in an independent sample. This association was also true for bipolar I disorder, but not for other subtypes, possibly due to their limited sample sizes in the EWAS and different biological mechanisms. Finally, we showed that, despite the pervasive influence of SNP variation on DNA methylation, PMS explained variance in BD independently of genetic variation, and that combining PMS with polygenic risk scores improved the prediction of BD.

**Implications of all the available evidence**

Available evidence indicates that epigenome-wide DNA methylation changes associated with BD can be identified in peripheral tissues. While GWAS will require substantial effort to explain larger biological effects and improve predictive tools, EWASs could complement those with relatively smaller sample sizes. PMS from adequately powered EWASs have the potential to serve as biomarkers of BD and its subtypes, in combination with polygenic scores. Future large-scale longitudinal EWAS, including medication-naïve patients, may help elucidate the biological changes that occur throughout the course of BD.

## Introduction

Bipolar disorder (BD) is a severe mood disorder characterised by episodes of depression and mania or hypomania. The lifetime prevalence of broadly defined BD is approximately 2.4% globally, and a BD diagnosis is associated with substantial disability and increased suicide risk.^1^ BD is a highly polygenic phenotype with substantial contribution from genetic factors.^2^ Environmental factors, including prenatal factors, have also been linked to risk, onset, and long-term course of BD.^3^ The liability threshold model of multifactorial mental disorders suggests contribution from both genetic and environmental risk factors, where combined liability crosses an unobserved threshold to develop the disorder.

Epigenetic mechanisms, such as DNA methylation, which capture both genetic and environmental effects, may provide insight into the aetiology of mental disorders. Differences in DNA methylation have been linked to exposure to non-genetic factors such as stressful events or birth complications.^4^ Adequately powered epigenome-wide association studies (EWASs), employing rigorous analytical methods, can help identify biological changes in peripheral tissues (e.g., blood) associated with disease states. To this end, relatively large EWAS meta-analyses of schizophrenia, major depression (MDD), and posttraumatic stress disorder (PTSD) have shed light on the biological mechanisms of these respective disorders.^5^, ^6^, ^7^ However, similarly powered EWASs in BD do not exist. Differentially methylated regions (DMR) have been associated with BD, for instance, in genes that were identified in genome-wide association studies (GWAS) of BD.^8^ However, until now, most studies were underpowered, and the reported associations seldom reached genome-wide significance.

The considerable advances in identifying genetic factors for BD have led to the development of polygenic scores (PGS), which currently explain only approximately 7% of the BD liability.^2^ Current power estimates suggest an effective sample size of 2.5 million will be required for BD GWAS to capture 50% of its genetic variance.^9^ Clinically meaningful application of PGS requires integration with environmental factors, which contribute to the risk of BD independently or through gene–environment interactions.^3^^,^^10^ Polymethylation scores (PMS) derived from adequately powered EWAS of BD, by accounting for biological embeddings of environmental factors, may complement PGS as prediction tools to support clinical decision making. Studies have demonstrated that PMS explained a small yet significant proportion of the variance for schizophrenia and MDD,^5^^,^^11^ and a considerable proportion (36%) of the variance for PTSD—a primarily environment-triggered condition.^12^ Notably, these PMS were derived from considerably smaller sample EWAS compared to GWAS of the respective phenotypes. Although EWAS-derived PMS for BD have not been tested, DNA methylation levels at seven DMRs, combined with other variables, accurately classified lithium response in 90% of individuals with BD,^13^ highlighting the potential for clinical translation of epigenetic markers.

Here, we report findings from the largest meta-analysis of EWASs on BD to date, comprising 3476 individuals. We applied rigorous, harmonised quality control pipelines ensuring consistent data quality, and included samples from different populations.^14^ We employed sex-informed meta-analyses to account for potential sex differences.^5^ We identified DMPs and DMRs associated with BD and highlighted the biological pathways impacted. We demonstrated that PMS were significantly associated with BD and improves the variance explained when combined with PGS.

## Methods

### Cohort information

Members of the Psychiatric Genomics Consortium Bipolar Disorder Working Group (PGC-BD) were approached and invited to contribute to this EWAS of BD effort (Montréal 2023). Additional PGC-BD members and authors identified in published literature on DNA methylation in BD were contacted and invited to collaborate. Cohorts were required to include both individuals with BD and controls with DNA from whole blood typed on methylation microarrays. A total of 3476 participants (cases = 1729 and controls = 1747) from 12 cohorts were included in the analyses. The mean ages ranged from 16.4 to 53.4 years, and 56% were females (Table 1 and Table S1).

**Table 1:** Contributing cohorts of bipolar disorder by sex, clinical subtype, and epigenome-wide analysis approach.

Cohort/sample (country)	Cases					Controls	
	All	Females	BDI	BDII	BDNOS	All	Females
BIPOGENT-IPM (Spain)^§,∗^	74	51 (69%)	59	13	2	89	60 (67%)
FOR2107 (Germany)^§,∗^	96	50 (52%)	50	46	0	242	151 (62%)
Halifax-Cagliari (Canada and Italy)^§,∗^	256	163 (64%)	154	92	10	47	34 (72%)
IGPF (Australia)^‡^	24	14 (58%)	24	0	0	34	19 (56%)
IGPB (Australia)^‡^	37	27 (73%)	37	0	0	23	10 (43%)
PDMH (United States)^§,‡^	35	11 (31%)	25	4	6	144	13 (9%)
TOP1 (Norway)^§,∗^	183	106 (58%)	113	56	14	278	111 (40%)
TOP3 (Norway)^§,∗^	166	102 (61%)	101	8	57	440	208 (47%)
TOP+ (Norway)^§,∗^	588	339 (58%)	270	265	53	326	180 (55%)
Unica-BD (Italy)^§,∗^	90	58 (64%)	59	26	6	32	18 (56%)
UNSW-BipolarKids&Sibs (Australia)^‡^	17	11 (65%)	3	12	2	16	7 (44%)
UTHealth Houston (United States)^§,∗^	163	118 (72%)	142	21	0	76	49 (64%)
**Total**	**1729**	**1050 (61%)**	**1037 (60%)**	**543 (31%)**	**149 (9%)**	**1747**	**860 (49%)**

BDI: bipolar I disorder, BDII: bipolar II disorder, BDNOS: bipolar disorder not otherwise specifiedModels of epigenome-wide association analysis applied for each cohort: ^∗^Female-only, ^§^Male-only, and ^‡^Sex-adjusted.

N.B. For the PDMH cohort, the male-only summary statistics were excluded from the total sample meta-analysis since the sex-adjusted results included both sexes.

### Ethics statement

The respective ethics committees approved the study protocols for each cohort in accordance with the Declaration of Helsinki, and all participants provided written informed consent. Approvals were obtained from: the IISPV Ethics Committee (Spain, 24/09/2015) for BIPOGENT-IPM; the local Ethics Committees of Marburg (AZ:07/14) and Münster (AZ:2014-422-b-S) for FOR2107 (Germany); the Nova Scotia Health Authority (REB FILE #1020604, Canada) and the University of Cagliari (#348/FC/2013 and PG/2018/11,693, Italy) for the Halifax-Cagliari cohorts; the UNSW Human Research Ethics Committee (HC12384), St. Vincent's Hospital (HREC/10/SVH/9), and the South East Sydney and Illawarra Area Health Service (HREC 09/081) for IGPF and IGPB (Australia); the VA hospital's local institutional review board (#1596360) for PDMH (USA); the Norwegian Scientific-Ethical Committee and Data Protection Agency (REK #2009/2485) for the TOP project (TOP1, TOP3, TOP+); the University of Cagliari (#348/FC/2013 and #PG/2018/11,693) for Unica-BD (Italy); the UNSW Human Research Ethics Committee (HREC Protocol 09/097) for UNSW-BipolarKids&Sibs (Australia); and the local institutional review board (#HSC-MS-09-0340) for UTHealth Houston (USA).

### Recruitment and phenotypic ascertainment

Cases included in the contributing cohorts were recruited from outpatient clinics, hospitals, or identified from registries. A diagnosis of bipolar I disorder (BDI), bipolar II disorder (BDII), or unspecified BD (BDNOS) according to DSM-IV/DSM-IV-TR/DSM-5 or ICD-10 was ascertained through semi-structured clinical interviews (e.g., SCID). Controls were recruited from communities residing in the same catchment area as the cases (or from populations generally comparable to the cases). Semi-structured clinical interviews and additional information were used to exclude individuals with personal or first-degree family history of psychosis, mood disorders, and substance use disorders. Detailed information on each cohort is provided in the Supplementary File.

### DNA methylation: data preparation, quality check and analysis

Genomic DNA was extracted from peripheral blood. DNA methylation data was obtained from methylation arrays: 450K; EPIC array v1, or EPIC array v2 (Illumina Inc., San Diego, CA, United States). A common quality control (QC) pipeline based on CPACOR was applied to each dataset.^15^ The QC consisted of several steps, including background correction followed by probe- and sample-level filtering, as described below.

First, background correction of the intensity values was performed using the minfi ‘bgcorrect.illumina’ function.^16^ Second, samples were removed if the bisulfite conversion rate was less than 80%,^17^ if they had a mismatch between predicted and reported sex, if they were outliers in the minfi sex plot, if they had a low call rate (<95% of probes had a detection *p*-value <1 × 10^−16^ for autosomes and *p*-value <1 × 10^−05^ for sex chromosomes), or if they appeared as outliers on visual inspection of principal component and beta distribution plots. Third, probes were removed if they had low bead counts (>5% of samples with counts <3), low probe call rate (less than 95% of the samples have a detection *p*-value <1 × 10^−16^ for autosomes and *p*-value <1 × 10^−16^ for sex chromosome probes), or were known to have low quality based on previous publications: cross-reactive probes, SNP probes, flagged probes and probes with mapping inaccuracies according to Illumina (EPICv2).^18^^,^^19^ Fourth, the intensity values for methylated and unmethylated signals were quantile-normalised before computing beta values, which were then normalised using BMIQ (for autosomes only).^20^ Finally, missing values were imputed using K-nearest neighbour by applying the Chip Analysis Methylation Pipeline (ChAMP).^21^ Replicated probes were handled similarly to the *rmPosReps(.)* function of the DMRcate package, with precision prioritized over sensitivity and weighted averages instead of standard averages calculated.^18^^,^^22^ CpGs with overlapping SNPs and MAF >0.05 were excluded during quality control but not from genetic ancestry (https://github.com/KiraHoeffler/EpiAnceR).^14^

### Extraction and estimation of covariates from the methylation data

Data on variables that potentially confound the associations between methylation and the phenotype were extracted or estimated from the methylation data. These variables were included as covariates in the downstream statistical models (Table S1).

*Cell proportions* were estimated using the Houseman algorithm, as implemented by the Bioconductor package FlowSorted.Blood.EPIC. This package uses an optimised library of CpGs, combined with a reference set of measured cell proportions for the deconvolution.^23^ The standard function *estimateCellCounts2* was used for array types 450K and EPIC v1, while *projectCellType_CP* was used for EPIC v2, due to the need to handle replicate probes on this array before deconvolution. After deconvolution, principal components (PC) were calculated. *Ancestry* was estimated based on array-specific SNP probes overlapping with CpGs, combined with SNP probes on the arrays, and adjusted for technical and biological factors. Ancestry PCs were calculated and adjusted for in the analyses.^14^ *Control probes*: PCs were calculated from the positive control probes on the Illumina arrays to adjust for technical variation. *Smoking status*: the M-values of the probe cg05575921 were used as a measure of smoking.^24^

### Epigenome-wide association analyses

Methylation beta values were transformed to M-values and regressed on BD case–control status using the limma package. The linear regression models were adjusted for age, smoking status, five cell proportion PCs, ancestry PCs, and control probe PCs. Sex-specific analyses were performed for cohorts with at least 35 males or females to minimise spurious associations from overfitting. For cohorts with fewer participants, analyses were restricted to sex-adjusted models, using sex as a covariate. We ran several models, including combinations of different numbers of ancestry PCs (two, five, or 10) and control probe PCs (two, five, 10, or 15). The covariates used in the models for the EWASs of each cohort are provided in the Supplementary Tables (Table S1). We selected the model with the least inflation, as evidenced by the appearance of QQ plots and the lambda value closest to 1. Control probe PCs that showed multicollinearity with sex were excluded from the models. Bacon correction was applied to the TOP cohorts to reduce inflation in the QQ plots using the *bacon* package.

### Meta-analysis

All meta-analyses were performed using the *metagen* function of the R package *meta* (https://CRAN.R-project.org/package=meta). Effect sizes and standard errors from each cohort-specific EWAS were meta-analysed using random effects models. CpGs present in at least two cohorts were meta-analysed. Three meta-analyses were conducted: female-specific (eight cohorts), male-specific (nine cohorts), and sex-agnostic (12 cohorts). A study-wide significance threshold was set as recommended based on 5% family-wise error.^25^ As this threshold was derived for EPIC v1, we adjusted for the additional EPIC v2 probes (n = 136,784), resulting in a final threshold of 7.2 × 10^−08^.

### Sensitivity analysis

*Leave-one-out meta-analyses:* To test whether a particular cohort was driving the association of significant findings in the meta-analysis, we performed leave-one-out meta-analyses for each DMP. In each iteration of the meta-analysis, one cohort was left out, and the summary statistics were compared using forest plots.

*Age moderation analysis:* Meta-regression analyses were conducted to evaluate whether age influenced the results of the DMPs identified in the meta-analysis. A mixed-effects model was fitted using the *rma* function from the R package *metafor*. Cohort-specific effect estimates and their standard errors were entered into the model, with age specified as a moderator. The between-study variance was estimated using the default restricted maximum-likelihood (REML) method.

### Differentially methylated regions

DMRs were identified using the *comb-p* procedure in Python,^26^ with a seed *p*-value of 0.001 and a maximum probe distance of 750 base pairs. DMRs containing at least four probes were considered significant at a threshold of *p*< 0.05 after Sidak correction for multiple testing (Stouffer–Liptak test) with a minimum of four probes.^26^

### Annotation

CpG sites were annotated in a stepwise manner, proceeding to the next step only if no annotation was obtained in the previous one. Step 1–Regulatory Region Proximity: CpGs were first annotated to the nearest gene based on proximity to transcription start sites (TSS) of regulatory regions, as defined by ENCODE (downloaded from https://screen.wenglab.org/downloads, April 2025). Step 2–Proximity to TSS or Exon 1: CpGs located within 2000 base pairs upstream of a gene's TSS or within exon 1 were annotated to that gene using RefSeq gene annotations obtained from the UCSC Genome Browser.^27^ Step 3–Gene Body Location: CpGs located within the gene body were annotated using RefSeq gene annotations.^27^

### Correlation of blood and brain DNA methylation

Using online platforms, BECon and the Blood Brain DNA Methylation Comparison Tool,^28^^,^^29^ we examined the correlation between DNA methylation in blood and brain for the identified DMPs. We also examined whether any of the DMRs overlapped with the list of blood–brain correlated CpGs as described previously.^30^

### Overlap with GWAS catalogue

The GWAS catalogue was screened for the DMR regions and the DMP regions (±25 kb) using https://genome.ucsc.edu/cgi-bin/hg.^27^ The list was filtered for BD, for mental disorders with known genetic overlap with BD, for brain morphology (i.e., volume and thickness), and for cognitive traits (including educational achievement).

### Pathways

The top 0.5% of CpGs with the lowest *p*-values in the sex-agnostic meta-analysis for gene set analysis were annotated in the R/Bioconductor package *missMethyl*, using gene sets available through the functions *goMeth*, with collection “GO”, using the function *gsameth*. For clustering, all pathways with false discovery rate (FDR) < 0.05 were uploaded to REVIGO (http://revigo.irb.hr/),^31^ filtering out gene sets >1000 genes.

### Post-hoc analysis

Based on the gene ontology results, which revealed an enrichment of immune system-related pathways, we next examined the association between BD case–control status and estimated immune cell-type proportions. For each of the six immune cell types, cell proportion was regressed on BD status, adjusting for age and sex. Study-specific effect estimates and standard errors were subsequently meta-analysed across cohorts using a random-effects model implemented in the *meta* R package.

### Polymethylation and polygenic scores

The TOP + cohort (cases = 588 and controls = 326) was used as a test sample for PMS. The discovery data, consisting of all cohorts except TOP+ (N = 2562), were meta-analysed to produce summary statistics for the PMS (BD-PMS). The BD-PMS weights for each CpG in the TOP + cohort were computed as the sum of weighted beta values for the corresponding CpGs in the discovery meta-analysis summary statistics. To remove correlated CpGs from the PMS calculation, we used CoMeBack to identify correlated regions defined as CpGs <500bp apart with correlation >0.3.^32^ A pipeline developed by Chen et al. was then used to calculate the PMS.^33^ For each co-methylated region identified by CoMeBack, the CpG with the lowest *p*-value is selected to represent the region. CpGs that are not in co-methylated regions are added as singletons. PMS were calculated using a thresholding method using *p*-value thresholds ranging from 5 × 10^−02^ to 5 × 10^−11^ in the TOP + cohort. Principal component analysis was then applied to the full set of PMS, and the first PC was used for the association analyses. Similarly, PMS of schizophrenia (SCZ-PMS), MDD (MDD-PMS), and PTSD (PTSD-PMS) were calculated using the respective EWAS summary statistics.^5^, ^6^, ^7^

In the TOP + cohort, we tested whether the BD-PMS, SCZ-PMS, MDD-PMS, and PTSD-PMS were associated with BD case–control status. We also tested the association of BD-PMS with medication type and smoking. The BD-PMS associations with different psychotropic medications were tested by comparing individuals on one medication with those on the other medications. The samples were: on antipsychotics (N = 67 versus 88 ‘controls’), antidepressants (N = 46 versus 109 ‘controls’), anti-epileptic (N = 59 versus 96 ‘controls’), and lithium (N = 29 versus 126 ‘controls’). Since cg05575921 methylation was a covariate in the discovery analysis, we calculated smoking scores in the 588 patients using the method developed previously,^34^ using an existing script.^35^ The association between smoking scores and BD-PMS was then tested using linear regression.

PGS was calculated using the recent Psychiatric Genomics Consortium (PGC) BD GWAS, excluding TOP and 23andMe cohorts, as the discovery sample comprising 58,309 cases and 775,699 controls.^2^ PGS were calculated for 498 cases and 287 controls in the TOP + cohort using PRSice at 11 thresholds (5 × 10^−08^ to 1).^36^ The first PC, calculated across the PGS thresholds, was then tested for association with BD case–control status in the TOP + cohort. A multiple regression model including BD-PGS and BD-PMS, along with five genetic PCs, was run against the BD case–control status. The different models were compared to each other and to a null model containing only 5 genetic PCs using ANOVA to obtain *p*-values. The explained variance was calculated using Nagelkerke's R^2^. To test the predictive ability of the BD-PGS, BD-PMS, and the combination of BD-PGS + PMS, area under receiver's characteristic curve (AUC) were calculated using the function *‘auc’* from the pRoc package. We employed a Monte Carlo cross-validation framework to estimate the predictive performance and its variability.^37^ In each of 1000 iterations, an 80% random sample of the TOP + cohort was used as the training set, with the complementary 20% serving as the independent test set. The AUC was computed for each iteration. The results are summarized as the mean AUC and the 95% confidence interval derived from the distribution of the 1000 AUC values.

### Role of funders

The study sponsors had no role in study design; in the collection, analysis, and interpretation of data; in the writing of the report; and in the decision to submit the paper for publication.

## Results

### Summary of cohort demographics and processing

**Fig. 1:**
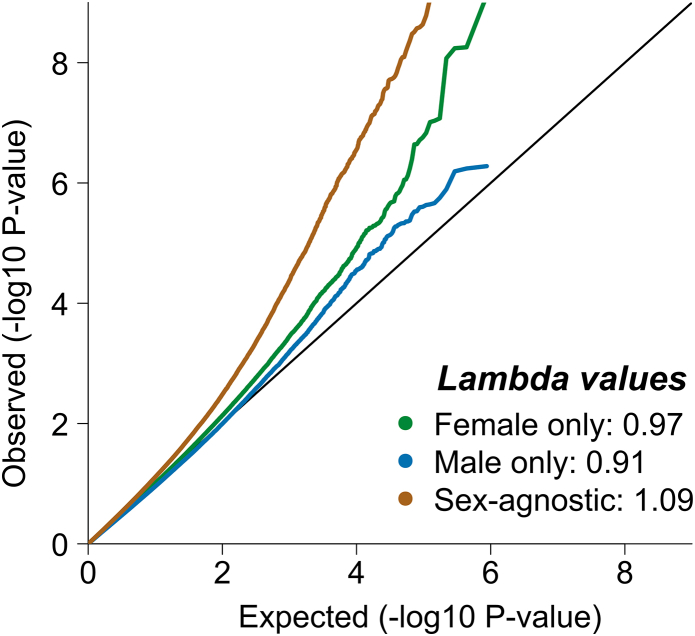
QQ plots of *p*-values (Z-test) in female-only, male-only, and total sample EWAS meta-analyses of bipolar disorder. The genomic inflation factor, lambda values 0.95–1.10 indicate minimal or no inflation relative to the expected null distribution (black line).

Most of the cases (60%, N = 1037) had a diagnosis of BDI, and the remaining 40% had a diagnosis of BDII (N = 543) or BDNOS (N = 149) (Table 1). EWASs were performed separately in males and females, except for three samples in which the number of males or females was <35, for which analyses were performed for the whole sample, adjusting for sex as a covariate. The final samples for EWAS meta-analyses comprised female-only (N = 1835), male-only (N = 1503), and total sample (N = 3476). The QQ plots and lambda values do not show inflation (Fig. 1 and Figures S1–S3).

In the total sample, we identified 47 DMPs associated with BD diagnosis (*p*< 7.2 × 10^−08^), 12 of which were hypermethylated and 35 hypomethylated. In sex-stratified meta-analyses, we identified four DMPs in female-only and no DMPs in male-only analyses (Table 2; and Tables S2 and ST3). The DMPs had the same effect directions across most samples as those observed in the total sample meta-analysis. Additionally, their overall effect remained significant in the Leave-One-Out meta-analyses (Figure S4). No age effects were observed for the DMPs (Table S4).

**Table 2:** Differentially methylated CpGs in blood associated with bipolar disorder in the total sample and female-only analyses.

CpG name	Meta-stats count	*p*-value^a^	Genomic position (GRCh38)	Relation to CpG Island	ENCODE enhancer	Type of enhancer	Annotated gene(s)
**Total sample**							
cg18436544	18	8.40 × 10^−12^	chr13:24,251,643	OpenSea	EH38E1660788	distal enhancer	*SPATA13-AS1*
cg26689077	20	4.29 × 10^−11^	chr12:53,206,022	OpenSea	EH38E1613313	proximal enhancer	*ITGB7*
cg12836863	20	7.52 × 10^−11^	chr13:32,314,886	Shore	EH38E1665800	proximal enhancer	*BRCA2*
cg15958828	20	2.08 × 10^−10^	chr3:71,549,229	OpenSea	EH38E2213519	distal enhancer	*MIR1284*
cg01538969	20	5.08 × 10^−10^	chr6:30,656,859	OpenSea			*DHX16*
cg04418434	20	8.03 × 10^−10^	chr6:7,110,540	Shore			*RREB1*
cg03460132	18	8.58 × 10^−10^	chr1:67,732,921	OpenSea	EH38E1357223	distal enhancer	*RNU7-80P*
cg15300024	18	1.65 × 10^−09^	chr2:112,729,590	OpenSea	EH38E2025511	distal enhancer	*NT5DC4*
cg07687574	18	2.32 × 10^−09^	chr17:8,941,264	OpenSea	EH38E1845691	distal enhancer	*PIK3R5*
cg13240557	18	2.58 × 10^−09^	chr3:52,872,546	OpenSea			*STIMATE-MUSTN1; STIMATE*
cg26720491	18	2.68 × 10^−09^	chr1:167,548,529	OpenSea	EH38E1394729	distal enhancer	*CREG1*
cg11222608	18	3.08 × 10^−09^	chr16:85,733,231	OpenSea			*C16orf74*
cg24410257	18	3.32 × 10^−09^	chr10:30,459,524	OpenSea	EH38E1459049	distal enhancer	*MAP3K8*
cg07869232	20	4.30 × 10^−09^	chr13:114,024,873	Shelf	EH38E1700448	distal enhancer	*C13orf46*
cg05508862	20	5.28 × 10^−09^	chr17:18,982,124	OpenSea	EH38E1851217	distal enhancer	*SLC5A10;FAM83G*
cg13241645	20	6.46 × 10^−09^	chr3:52,201,943	Shelf	EH38E2202126	distal enhancer	*ALAS1*
cg25247520	20	8.05 × 10^−09^	chr8:127,795,771	Shore	EH38E2666248	promoter	*PVT1*
cg00565090	20	8.10 × 10^−09^	chr19:47,755,001	OpenSea	EH38E1959550	proximal enhancer	*SNORD23*
cg13549638	20	8.89 × 10^−09^	chr17:80,886,276	Shelf	EH38E1891548	distal enhancer	*RPTOR*
cg13947735	20	1.07 × 10^−08^	chr17:82,967,093	Shelf	EH38E1893991	distal enhancer	*B3GNTL1*
cg12753728	18	1.24 × 10^−08^	chr17:10,021,552	OpenSea	EH38E1846384	proximal enhancer	*GAS7*
cg26538377	18	1.46 × 10^−08^	chr15:89,112,208	OpenSea			*RNU7-195P*
cg22123711	20	1.64 × 10^−08^	chr1:12,125,788	OpenSea	EH38E1319404	promoter	*TNFRSF8*
cg21369801	20	1.75 × 10^−08^	chr17:82,245,085	Island			*CSNK1D*
cg06980173	20	1.81 × 10^−08^	chr1:154,403,868	Shore	EH38E1386103	proximal enhancer	*IL6R*
cg07249152	18	1.86 × 10^−08^	chr2:8,299,087	OpenSea	EH38E1970735	distal enhancer	*LINC00299*
cg19204843	18	1.89 × 10^−08^	chr4:184,355,208	OpenSea			*LINC02363*
cg14463959	18	1.91 × 10^−08^	chr1:159,085,077	OpenSea	EH38E1389616	distal enhancer	*RAD1P2*
cg12116137	20	1.99 × 10^−08^	chr17:1,673,155	OpenSea	EH38E1840205	distal enhancer	*PRPF8*
cg27663181	18	2.57 × 10^−08^	chr1:111,489,873	OpenSea	EH38E1376392	promoter	*RNU6-792P*
cg05380077	18	2.68 × 10^−08^	chr11:63,504,753	OpenSea			*LGALS12*
cg18532548	18	2.69 × 10^−08^	chr12:45,219,778	Shelf	EH38E1607189	distal enhancer	*ANO6*
cg26262644	18	2.76 × 10^−08^	chr15:73,921,905	OpenSea			*LOXL1-AS1*
cg10519313	18	2.92 × 10^−08^	chr2:201,209,130	OpenSea	EH38E2065932	distal enhancer	*MTND5P25*
cg15349696	20	3.20 × 10^−08^	chr17:82,587,558	Island			*FOXK2; LOC105371942*
cg16357878	18	4.04 × 10^−08^	chr11:47,988,101	OpenSea	EH38E1538648	distal enhancer	*PTPRJ*
cg13876222	20	4.21 × 10^−08^	chr9:136,504,896	Island			*NOTCH1*
cg01909551	20	4.57 × 10^−08^	chr3:52,189,935	OpenSea			
cg12022722	18	4.88 × 10^−08^	chr19:39,338,019	Shelf	EH38E1953241	proximal enhancer	*GMFG*
cg05244443	18	5.01 × 10^−08^	chr6:81,941,408	OpenSea	EH38E2481981	distal enhancer	*LINC02542*
cg08606580	18	5.12 × 10^−08^	chr17:27,531,689	OpenSea	EH38E1853271	distal enhancer	*MSANTD3P1*
cg19739596	20	5.41 × 10^−08^	chr11:60,056,688	OpenSea			*MS4A3*
cg02862467	20	5.88 × 10^−08^	chr1:19,081,403	OpenSea	EH38E1324297	proximal enhancer	*UBR4*
cg18556420	20	6.12 × 10^−08^	chr10:818,656	OpenSea			*LARP4B*
cg00094412	20	6.37 × 10^−08^	chr6:29,625,077	Shelf	EH38E2457791	distal enhancer	*GABBR1*
cg24325900	8	6.43 × 10^−08^	chr8:10,028,315	OpenSea			
cg03640051	20	7.09 × 10^−08^	chr4:42,655,672	Shore			*ATP8A1*
**Females**							
cg03497652	8	7.63 × 10^−10^	chr16:4,701,568	OpenSea			*NUDT16L1*
cg27003968	8	5.54 × 10^−09^	chr19:41,206,079	OpenSea	EH38E1954604	Distal enhancer	*CYP2S1*
cg26450266	8	5.75 × 10^−09^	chr17:18,246,373	Shore			*FLII*
cg00565090	8	8.45 × 10^−09^	chr19:47,755,001	OpenSea	EH38E1959550	Proximal enhancer	*SNORD23*

a Z-test using study-wide significance threshold set at 7.2 × 10^−08^ using family-wise error and accounting for additional probes from EPIC v2 array.

**Fig. 2:**
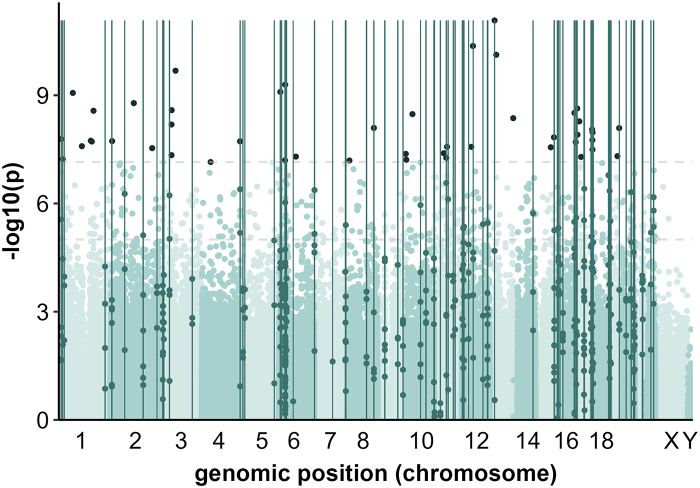
Manhattan plot of differentially methylated regions (DMR) associated with bipolar disorder in meta-analysis of the total sample. Y-axis: -log10 of *p*-values (Z-test) of CpGs; chromosomes: x-axis; dark-green vertical lines represent an identified DMR; dark-green beads represent CpGs.

We also identified 90 DMRs in the total sample, 14 for female-only, and six for male-only meta-analyses (Fig. 2, and Tables S5–S7). Ten and three of the DMRs in the total sample were mostly driven by female or male samples, respectively. This includes a DMR that maps to *VARS2*, a mitochondrial aminoacyl-tRNA synthetase previously identified through a DMP in a PRS-stratified EWAS.^38^ Several of the DMRs identified in the current study were previously reported in association with BD, or related phenotypes: *FURIN, DDR1, DOC2A, CACNA2D4, CSNK1D, GABBR1, STAT3* and *SOCS3*.

### Pathways–gene ontology

The largest cluster of pathways were observed for biological processes involving the activation, regulation, and execution of the immune response, particularly within innate and adaptive immunity (e.g., regulation of cytokines, TNF superfamily cytokines). The second cluster comprised biological processes related to phosphorylation and phosphorus metabolism pathways, which are crucial for cell signalling and regulation of protein function (e.g., macromolecule phosphorylation). The third cluster consisted of cellular components related to the compartmentalisation and trafficking of molecules for degradation, immune defence, and secretion. The fourth cluster included molecular functions, such as nucleoside-triphosphate activity and enzyme regulator activity (Table S8; Figures S5: A–D).

### Estimated blood cell proportion differences (*post-hoc analysis*)

**Fig. 3:**
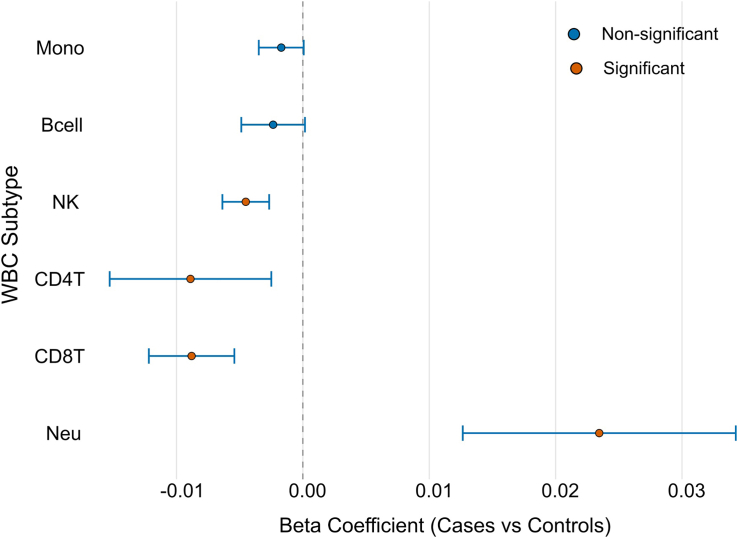
Random-effects meta-analysis of associations of estimated blood cell type proportions in cases of bipolar disorder compared to controls. NK; natural killer, Neu; neutrophils, Mono; monocytes, Bcell; B lymphocytes, CD4T; CD4 T lymphocytes, and CD8T; CD8 T lymphocytes.

In linear regression adjusted for age and sex, estimated blood cell proportions between BD cases and controls showed significantly higher neutrophils (β = 0.023; Z-test, adjusted *p*-value <0.001). On the other hand, CD4T (β = −0.009; Z-test, adjusted *p*-value <0.05), CD8T (β = −0.009; Z-test, adjusted *p*-value <0.001), and natural killer cells (β = −0.004; Z-test, adjusted *p*-value <0.001) had lower proportions in BD cases compared to controls. The proportions of B-cells and monocytes were not significantly different in the two groups (Z-test, adjusted *p*-value >0.05) (Fig. 3).

### GWAS overlap

We investigated whether genes annotated to DMPs and DMRs have previously been implicated in GWAS of traits relevant to BD. These traits included psychiatric disorders, brain morphology, and cognition. A search of the GWAS catalogue identified 163 trait-associated genomic regions overlapping with the DMPs or DMRs (Table S9). After restricting the search to regions associated with psychiatric disorders, brain morphology, or cognition, 16 regions remained (Table S10). These regions correspond to the following genes: *GHRL, STIMATE, mir1284, RREB1, NOTCH1, DCDC1, OLR1, ITGB7, B3GNTL1, GAS7 and KDM4B*.

### Brain correlation

In BECon, cg13241645 and cg22123711 showed the highest correlations (Figures S6–S8). Four DMPs were significantly correlated between blood and brain regions: cg13876222 (*NOTCH1*)–prefrontal cortex; cg05508862 (*SLC5A10; FAM83G*)- entorhinal cortex; cg15349696 (*FOXK2*) and cg00565090 (*SNORD23*)–cerebellum (Tables S11 and S12; Figures S9–S12). The DMR chr5:8457425-8457857, annotated to *LINC02226* and *MIR4458HG,* was shown to present a significant correlation between blood and brain tissue. Among the genes annotated to the identified DMPs and DMRs in this study, *RAB44*, *GET4, CLMN, PIK3R6, B3GNTL1*, and *ERG* overlapped with genes reported to be differentially methylated in the prefrontal cortex of BD cases.^8^

### Polymethylation and polygenic scores

**Fig. 4:**
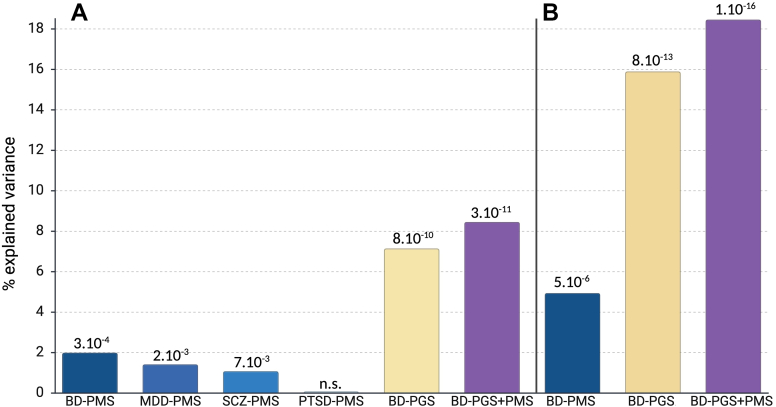
Association between PMSs and PGS with BD (A), and BDI (B) case–control status. The numbers on the y-axis indicate the proportion of variance explained. X-axis: BD: Bipolar disorder, BDI: bipolar I disorder, MDD: Major depressive disorder, SCZ: schizophrenia, PTSD: posttraumatic stress disorder, PMS: polymethylation scores, PGS: polygenic score. The numbers on the top of the bars indicate *p*-values (Wald test) for the association.

BD-PMS, MDD-PMS, and SCZ-PMS, but not PTSD-PMS, had a statistically significant association with BD case–control status. The association with BD-PMS was stronger than that of MDD-PMS and SCZ-PMS (Wald test, *p*-value <0.05) (Fig. 4 and Table S13). BD-PMS were not associated with smoking, and the association with being on antipsychotic treatment (Wald test, *p*-value = 0.01) became non-significant when the model was adjusted for BD subtype (Table S13).

We compared the variance explained by BD-PMS and BD-PGS. It is important to note that the effective sample size for the discovery of PGS was 95-fold that of the PMS. In the model comparing BD cases and controls, the PGS explained 7.1% and the PMS explained 2.0% of the variance. A model combining BD-PGS and PMS explained 8.5% of the variance, representing a significant improvement over the model with BD-PGS alone (Likelihood ratio test, *p*-value = 0.0056). The increase in variance explained by the combined model was not fully additive, demonstrating that the BD-PGS and BD-PMS partially overlap. To evaluate the potential clinical utility of the model combining BD-PGS + PMS, we calculated AUC in the TOP + cohort and found that the combined model slightly improved the AUC compared to PGS alone (Table 3).

**Table 3:** Area under receiver's operating characteristic curve for the association between bipolar disorder case–control status, and polymethylation scores (BD-PMS) and polygenic scores (BD-PGS).

Phenotype in the target sample	BD-PMS [95% CI]	BD-PGS [95% CI]	BD-PGS + PMS [95% CI]
Bipolar disorder	0.675 [0.594, 0.750]	0.715 [0.644, 0.785]	0.719 [0.648, 0.788]
Bipolar I disorder	0.694 [0.600, 0.785]	0.752 [0.660, 0.838]	0.762 [0.666, 0.850]

In the analyses for BD subtypes in the TOP+, BD-PMS was associated with BDI but not BDII or BDNOS compared to controls. In models comparing BDI cases and controls, the variance explained by the BD-PMS and the BD-PGS were 4.9% and 15.9%, respectively. The model that included both BD-PGS + PMS explained 18.5% of the variance, which was significantly different from the BD-PGS model (Likelihood ratio test, *p*-value = 0.001). This translated into an increase of AUC from 0.75 for BD-PGS only to 0.76 for the model including BD-PGS + PMS.

## Discussion

Here, we investigated genome-wide methylation differences in blood-derived DNA in association with BD, using to our knowledge the largest sample to date. We identified 47 DMPs and 90 DMRs associated with BD, four additional DMPs and 14 DMRs in the female-only, and six DMRs in the male-only analyses. Some of those converged on genes identified in genetic studies. Pathways for immune activation of both innate and adaptive immune response, as well as phosphorylation were enriched. We further demonstrated a significant association between BD-PMS and BD diagnostic status. Additionally, BD-PMS improved the variance explained when combined with BD-PGS from 7.1% to 8.5%. The variance explained was even larger for BDI, up to 18.5% in the model BD-PGS + PMS. Altogether, our findings revealed that DNA methylation marks in peripheral blood are associated with BD, and the combination of PMS with PGS is potentially useful for developing biomarkers for clinical translation.

EWASs of complex phenotypes such as BD require larger sample sizes for the reliable discovery of DMPs, which likely have small effect sizes.^39^ The sample sizes of existing EWASs on psychiatric phenotypes are orders of magnitude smaller than their respective GWAS. Several candidate gene studies have reported associations between DNA methylation in peripheral tissues and BD phenotypes.^40^, ^41^, ^42^ Recent large-scale EWAS meta-analyses have demonstrated the potential for epigenetic profiling in schizophrenia, MDD, and PTSD.^5^, ^6^, ^7^ Our findings reveal that DNA methylation in peripheral tissues may be leveraged for epigenetic classification of individuals with BD from controls. Despite explaining 2% of the variance compared to 7% for the PGS, the PMS result is still remarkable given the EWAS discovery sample was only a small fraction (1.1%) of that of the GWAS.^2^ The independent association of PMS and the improvement in the variance explained by the model that included PMS and PGS has important implications. The identified DNA methylation differences may capture biological differences in BD that are not accounted for by genetic studies (e.g., environmental factors). Achieving further improvement in the explained variance of BD with genetic data alone would require increasing the sample size for GWAS by millions,^9^ which needs tremendous investment and coordination efforts. In contrast, EWAS of BD is only at its beginning, and the potential of PMS can be unleashed through a modest increase in samples. Such an increase may prove to be a cost-efficient approach, facilitating the development of a clinically applicable algorithm that complements those based on genetic tools.

The differential association of PMS with BDI versus BDII is consistent with the findings for PGS in the latest GWAS of BD,^2^ but definitive conclusions on putative molecular divergence require a larger sample of individuals with BDII. Despite its comparable prevalence in the community,^1^ BDII is often underrepresented in genetic and epigenetic studies.^2^^,^^43^ BD subtypes may have underlying biological differences; hence, discoveries reported in studies of BD may not necessarily apply to BDII. Therefore, EWASs with larger samples of BDII may help mitigate potential healthcare disparities for this subgroup of patients. Nonetheless, the findings point to the potential for PMS as an epigenetic classification tool in BD to aid clinical diagnostics. In support, a preliminary study has shown DNA methylation-based tools could predict lithium response in individuals with BDI.^44^

The diagnostic criteria and symptomatology of BD considerably overlap those of MDD and schizophrenia.^45^ Genetic studies indicate that shared genetic factors may underlie the phenotypic overlap.^43^^,^^46^ The association of MDD-PMS and SCZ-PMS with the diagnosis of BD in the current study provides further support for overlapping biology. In addition to being driven by shared genetic factors, the shared DNA methylation marks across disorders may signify epigenetic embeddings of shared environmental risk factors (e.g., childhood trauma).

Gene set analyses highlight immune-related, phosphorylation, and phosphate metabolism pathways, which align with the existing literature and shed light on the biological changes associated with BD.^47^ Both brain and systemic immune activation have been linked to BD, and evidence suggests a causal link between immune phenotypes and BD.^48^ Similarly, the observed pattern of blood cell proportions in BD, with lower proportions of cell types involved in cell-mediated immunity, aligns with a state of chronic low-grade inflammation leading to suppression of adaptive immunity.^49^, ^50^, ^51^ Furthermore, the use of mood stabilizers and antipsychotics, which are commonly used in the treatment of BD, is associated with a reduction of circulating cytokines and downregulation of inflammation-associated genes.^52^ On the other hand, phosphorylation plays an important role in the regulation of enzymes, signal transduction, and energy metabolism. Some antipsychotic medications may exacerbate metabolic dysregulations in patients with BD.^53^ Antipsychotics and mood stabilizers have known effects on DNA methylation and histone marks.^54^ However, DNA methylation differences associated with antipsychotic treatment may be a result of the confounding effect of BD subtype. Our results are possibly driven by more frequent use of antipsychotic medications for BDI than for BDII.^55^

Several genes identified in the current study align with epigenetic associations found in related traits, including *GABBR1* in schizophrenia,^5^ as well as in childhood maltreatment,^56^ suggesting that DNA methylation changes mediate environmental risk for BD. Other genes annotated to DMRs, such as *RREB1*, *KDM4B*, *NOTCH1, FURIN, CACNA2D4, GAS7*, and *B3GNTL1,* were previously linked to neurodevelopmental processes or phenotypes.^57^

The findings from sex-stratified analysis suggest that DNA methylation signatures of BD may partly be sex-specific, although larger samples are required to increase the power to detect DMPs and DMRs and fully elucidate these differences. Given the sex differences in the prevalence of BD subtypes,^1^ an important question is whether clinical subtypes account for the observed epigenetic sex differences. Future studies designed to address this question must also consider other potential confounding factors, including sex disparities in comorbidities (e.g., substance use disorders) and treatment selection biases.^58^

Our findings should be interpreted in the light of some limitations. First, given the cell specificity of DNA methylation, DMPs in the brain associated with BD may only partially overlap with those observed in blood, limiting mechanistic insights into molecular changes in the brain. Secondly, pharmacological treatments of BD and remission status potentially influence DNA methylation. Future longitudinal studies involving treatment-naive individuals with BD are needed to elucidate the DNA methylation changes in peripheral tissues associated with disease status. Thirdly, the small sample of individuals with BDII, coupled with the predominance of the BDI subtype, limits the robustness of findings related to methylation scores and other BD subtypes. Fourth, most study participants are of European ancestry, and findings may not be generalisable to other populations. Fifth, the relatively smaller samples for sex-specific analyses may limit identification of DMPs; however, the identified DMRs suggest that some DNA methylation signatures of BD are sex-specific. Finally, DNA methylation is a dynamic process, and changes that may occur with the different clinical phases of BD (i.e., current mood states of depression, mania, mixed or euthymia) were not examined here. Future longitudinal studies, with DNA methylation profiles specifically examined during different phases of BD, may shed light on molecular predictors of both disease course and treatment effects.

Due to the limited power of the test sample for subgroups, we did not perform further analyses with PMS to explore potential overlaps of the different subtypes with other mental disorders. For example, genetic research suggests that schizophrenia shows stronger overlap with BDI, while MDD has stronger overlap with BDII.^43^ Likewise, we did not explore the potential moderating effect of specific environmental exposures posited to impact BD risk (e.g., childhood trauma). However, our results demonstrate that the study of DNA methylation from peripheral blood in BD cases has the potential to identify effects complementary to genetic studies and should therefore encourage the collection of larger datasets in the future.

In conclusion, we leveraged the largest collection of DNA methylation data on BD and found that DNA methylation profiles from peripheral tissue are associated with BD diagnosis and significantly improve PGS in classifying BD cases and controls. Despite the potentially complex relationship between DNA methylation signatures of BD in peripheral blood, disease biology, acute mood states, lifestyles, and medications, PMS in combination with PGS have the potential to reach a suitable level of sensitivity and specificity to become a clinically relevant biomarker. While international collaborative efforts in GWAS have brought an understanding of the disorder and led to PGS that can explain 15% of the variance in BDI, EWAS efforts to date have been limited. We now show that, even with a discovery sample which is approximately 1.1% of the GWAS, PMS can explain a significant proportion of the variance. Thus, PMS from larger EWASs have great potential to provide a valuable clinical prediction tool to complement genetic profiles. Clinical subtypes of BD need to be accounted for to gain a better understanding of the biological effects. These observations are likely to be relevant to other complex disorders and encourage greater investment in epigenetic studies.

## Contributors

Study Design: MT, AKS, SLH.

Data/sample collection: AJF, AK, AKS, AS, BC, BJO, CC, ES, EV, FS, GM, GR, GRF, GRo, GS, HJG, IM, JCB, JCS, JF, JL, JMF, KDH, KJB, LA, LM, LT, MA, MJG, MM, MP, MT, NAK, OAA, OJW, OKD, PBM, PP, PRS, RA, SD, SHS, SLH, SM, TK, TV, UD, VMS, Workgroup VA Mid-Atlantic MIRECC.

Manuscript writing: AKS, KDH, KSO, MT, SLH.

Manuscript revision: all.

Data analysis: AEA, AKS, AT, AW, CP, FSD, GRF, JMF, KDH, KSO, LS, MEG, MJG, MT, OJW, SLH.

VA Mid-Atlantic MIRECC Workgroup contributed to the overall design and conduct of the larger PDMH study, including obtaining funding, recruiting and enrolling participants, and conducting core study procedures (administering questionnaires and clinical interviews).

All authors read and approved the final version of the manuscript. Markos Tesfaye and Anne-Kristin Stavrum had access to all the summary statistics data that were meta-analysed and verified that they met the quality control standards.

## Data sharing statement

The analytical scripts are available on GitHub: https://github.com/codedbyanne/BIP-meta. Summary statistics are available upon request from Prof. Le Hellard; however, individual-level data is subject to data privacy restrictions.

## Declaration of interests

BC received speaking fees from Otsuka and Lundbeck outside the submitted work; MM reports grant from Lundbeck (speaker's honorarium), Fidia Farmaceutici (speaker's honorarium), Angelini (speaker's honorarium), Rovi (speaker's honorarium), Johnson and Johnson (speaker's honorarium); OAA is consultant to Ledidi, CorTechs.ai and Precision Health, and has received speaker's honorarium from BMS, Lilly, Janssen, Lundbeck, Sunovion and Otsuka. HJG has received travel grants and speaker's honoraria from Neuraxpharm, Servier, Indorsia, and Janssen Cilag. JMF reports speaker's honorarium from Illumina. JL has received honoraria for lectures or advisory boards from Janssen-Cilag, Otsuka, Lundbeck, Laboratorios Rovi, Angelini, Idorsia, and Casen Recordati. ES reports receiving support for travel and attendance at scientific and academic meetings from Janssen-Cilag, Otsuka Pharmaceuticals, and Lundbeck. F. Stein received travel grants and awards from SOBP, SIRS, Discourse, and WFSBP. JCS reports advisory board fees from ALKERMES; consulting fees from JOHNSON & JOHNSON and SUNOVIAN; and research grants from COMPASS PATHWAYS, MIND MED, and RELMADA. PRS has received consulting fees from Outside Opinion Pty Ltd, Moira Clay Consulting Pty Ltd and Neuroscience Research Australia. PRS is a Director of the Australian Dementia Network Ltd and the Childhood Dementia Network Ltd, both not-for-profit organizations, and serves as Chair of the National Medical Advisory Panel for the Mason Foundation.

## Appendix A Supplementary data

Supplementary Tables

Workgroup table PUBMED
