# Supplementary material for: DNA methylation signatures associated with bipolar disorder in peripheral blood improve prediction models
Source: eBioMedicine. 2026 May 8;128:106284. doi: 10.1016/j.ebiom.2026.106284 (PMC13187609; doi:10.1016/j.ebiom.2026.106284)
Supplement: Supplementary Material [file mmc1.docx]

**Supplementary information for:**

**DNA methylation signatures associated with bipolar disorder in peripheral blood improve prediction models**

Markos Tesfaye^1,2,3^ *, Anne-Kristin Stavrum^1^ *, Kira D. Höffler^1,4,5^, Kevin S. O'Connell^3^, Friederike S. David^6,7^, Melanie E. Garrett^8^, Sonia Hesam-Shariati^9,10^, Bronwyn J. Overs^9^, Claudia Pisanu^11^, Luana Spano^12^, Oliver J. Watkeys^13^, Antoine Weihs^14,15^, Rafaella Ardau^16^, Allison E. Ashley-Koch^8^, Lavinia Athanasiu^3^, Jean C. Beckham^17,18,19^, Kyle J. Bourassa^17,18,20^, Caterina Chillotti^16^, Srdjan Djurovic^3,21^, Ole K. Drange^3,22^, Josef Frank^23^, Anouar Khayachi^24^, Nathan A. Kimbrel^17,18,19^, Lourdes Martorell^25,26,27,28^, Susanne Meinert^29,30^, Ingrid Melle^31,32^, Gunnar Morken^33,34^, Pasquale Paribello^35^, Marco Pinna^35^, Gloria Roberts^36^, Guy Rouleau^24^, Peter R. Schofield^36^, Esteban Sepúlveda^25,26,27,28^, Giovanni Severino^11^, Vidar M. Steen^1,37^, Frederike Stein^7,38^, Fabian Streit^23,39,40,41^, VA Mid-Atlantic MIRECC Workgroup^17,18^, Trine Vik Lagerberg^42,43^, Martin Alda^44,45^, Udo Dannlowski^29,46,47,48^, Andreas J. Forstner^6,49^, Janice M. Fullerton^9,50^, Hans J. Grabe^14,15^, Melissa J. Green^13^, Tilo Kircher^7,38^, Javier Labad^28,51,52^, Mirko Manchia^35,53^, Philip B. Mitchell^36^, Jair C. Soares^54,55,62^, Alessio Squassina^11,44^, Alexander Teumer^15,56^, Leonardo Tondo^57,58^, Elisabet Vilella^25,26,27,28^, Ole A. Andreassen^3,59^, Boris Chaumette^12,60,61^, Gabriel R. Fries^54,55,62^, Stephanie Le Hellard^1,4^

1. Department of Clinical Sciences, University of Bergen, Bergen, Norway
2. Department of Psychiatry and Behavioral Sciences, Institute for Genomics in Health, State University of New York Downstate Health Sciences University, Brooklyn, NY, USA.
3. Centre for Precision Psychiatry, Division of Mental Health and Addiction, Oslo University Hospital and Institute of Clinical Medicine, University of Oslo, Oslo, Norway
4. Bergen Center for Brain Plasticity, Haukeland University Hospital, Bergen, Norway
5. Department of Psychiatry, McLean Hospital, Harvard Medical School, Belmont, MA, USA
6. Institute of Human Genetics, University of Bonn, School of Medicine & University Hospital Bonn, Bonn, Germany
7. Philipps-University, Department of Psychiatry and Psychotherapy, Marburg, Germany
8. Duke Molecular Physiology Institute, Duke University Medical Center, Durham, NC, USA
9. Neuroscience Research Australia, Randwick, Sydney, NSW, Australia
10. School of Psychology, University of New South Wales, Sydney, Australia
11. Department of Biomedical Sciences, University of Cagliari, Cagliari, Italy
12. Université Paris Cité, Institute of Psychiatry and Neuroscience of Paris (IPNP), INSERM, U1266, Paris, France
13. School of Clinical Medicine, University of New South Wales, Australia
14. German Center for Neurodegenerative Diseases (DZNE), Site Rostock/Greifswald, Greifswald, Germany
15. Department of Psychiatry and Psychotherapy, University Medicine Greifswald, Greifswald, Germany
16. Unit of Clinical Pharmacology, University Hospital Agency of Cagliari, Cagliari, Italy
17. VA Mid-Atlantic Mental Illness Research Education and Clinical Center, Durham, NC, USA
18. Durham VA Health Care System, Durham, NC, USA
19. Department of Psychiatry and Behavioral Sciences, Duke University School of Medicine, Durham, NC, USA
20. Department of Psychology, Georgetown University, Washington, DC, USA
21. Department of Medical Genetics, Oslo University Hospital and University of Oslo, Oslo, Norway
22. Department of Psychiatry, Sørlandet Hospital HF, Arendal/Kristiansand, Norway
23. Department of Genetic Epidemiology in Psychiatry, Central Institute of Mental Health, Medical Faculty Mannheim, Heidelberg University, Mannheim, Germany
24. Montreal Neurological Institute-Hospital, McGill University, Montréal, Canada
25. Hospital Universitari Institut Pere Mata, Reus, Spain
26. Institut d'Investigació Sanitària Pere Virgili-CERCA, Reus, Spain
27. Universitat Rovira i Virgili, Tarragona, Spain
28. CIBERSAM-Instituto de Salud Carlos III, Madrid, Spain
29. Institute for Translational Psychiatry, University of Münster, Münster, Germany
30. Institute for Translational Neuroscience, University of Münster, Münster, Germany
31. Section for Clinical Psychosis Research, Division of Mental Health and Addiction, Oslo University Hospital, Oslo, Norway.
32. Department of Adult Psychiatry, Institute of Clinical Medicine, University of Oslo, Oslo, Norway.
33. Department of Psychiatry, St Olavs University Hospital, Trondheim, Norway
34. Faculty of Medicine and Health Sciences, Norwegian University of Science and Technology (NTNU), Trondheim, Norway
35. Unit of Psychiatry, Department of Medical Sciences and Public Health, University of Cagliari, Cagliari, Italy
36. Discipline of Psychiatry and Mental Health, School of Clinical Medicine, Faculty of Medicine & Health, University of New South Wales, Sydney, Australia
37. Dr. Einar Martens Research Group for Biological Psychiatry, Department of Medical Genetics, Haukeland University Hospital, Bergen, Norway.
38. Center for Mind, Brain and Behavior, University of Marburg, Marburg, Germany
39. Hector Institute for Artificial Intelligence in Psychiatry, Heidelberg University, Mannheim, Germany
40. Department for Psychiatry and Psychotherapy, Central Institute of Mental Health, Mannheim, Germany
41. German Center for Mental Health (DZPG), Mannheim - Heidelberg - Ulm, Germany
42. Department for Research and Innovation, Division of Mental Health and Addiction, Oslo University Hospital, Oslo, Norway.
43. Department of Psychology, University of Oslo, Oslo, Norway.
44. Department of Psychiatry, Dalhousie University, Halifax, Nova Scotia, Canada
45. National Institute of Mental Health, Klecany, Czech Republic
46. Department of Psychiatry, Medical School and University Medical Center OWL, Protestant Hospital of the Bethel Foundation, Bielefeld University
47. German Center for Mental Health (DZPG), Site Jena Magdeburg Halle, Germany
48. Center for Intervention and Research on Adaptive and Maladaptive Brain Circuits Underlying Mental Health (C-I-R-C), Site Jena Magdeburg Halle, Germany
49. Institute of Neuroscience and Medicine (INM-1), Research Center Jülich, Germany
50. School of Biomedical Sciences, Faculty of Medicine & Health, University of New South Wales, Kensington, Sydney, NSW, Australia
51. Department of Mental Health and Addictions, Consorci Sanitari del Maresme, Mataró, Spain
52. Translational Neuroscience Research Unit, Universitat Autònoma de Barcelona, Spain
53. Department of Pharmacology, Dalhousie University, Halifax, Canada
54. Faillace Department of Psychiatry and Behavioral Sciences, McGovern Medical School, University of Texas Health Science Center at Houston, Houston, TX, USA
55. Neuroscience Graduate Program, UT MD Anderson Cancer Center UTHealth Graduate School, Houston, TX, USA
56. German Centre for Cardiovascular Research (DZHK), Partner Site Greifswald, Greifswald, Germany
57. Lucio Bini Mood Disorder Center, Cagliari, Italy
58. Department of Psychiatry, Harvard Medical School, Boston, MA, USA
59. K.G. Jebsen Centre for Neurodevelopmental Disorders, University of Oslo, Oslo, Norway
60. GHU-Paris Psychiatrie et Neurosciences, Hôpital Sainte Anne, Paris, France
61. Department of Psychiatry, McGill University, Montreal, Canada
62. School of Behavioral Health Sciences, University of Texas Health Science Center at Houston, Houston, TX, USA

* These authors have equally contributed to this work.

**Corresponding authors**

Stéphanie Le Hellard, Department of Clinical Sciences, University of Bergen, Postboks 7804

5020 Bergen, Norway,

[stephanie.lehellard@uib.no](mailto:stephanie.lehellard@uib.no)

Markos Tesfaye, Department of Psychiatry and Behavioral Sciences, Institute for Genomics in Health, SUNY Downstate Health Sciences University, 450 Clarkson Avenue, Psychiatry – MS 1203, Brooklyn, NY 11203, USA

[markos.woldeyohannes@downstate.edu](mailto:markos.woldeyohannes@downstate.edu)

Contents

[Samples Description 3](#_Toc226831035)

[BIPOGENT-IPM 3](#_Toc226831036)

[FOR2107 3](#_Toc226831037)

[HALIFAX-CAGLIARI 3](#_Toc226831038)

[Imaging Genetics in Psychosis 4](#_Toc226831039)

[PMDH 5](#_Toc226831040)

[TOP-TOP+ 5](#_Toc226831041)

[UNICA_BD 6](#_Toc226831042)

[UNSW Bipolar Kids & Sibs 6](#_Toc226831043)

[UTHealth Houston 7](#_Toc226831044)

[Supplementary Figures 8](#_Toc226831045)

[Figure S1(A-D): QQ plots for sex-adjusted EWAS samples. 8](#_Toc226831046)

[Figure S2 (A-H): QQ plots for female-only EWAS samples. 9](#_Toc226831047)

[Figure S3 (A-I): QQ plots for male-only EWAS samples. 10](#_Toc226831048)

[Figure S4: Forest plots and leave-one-out plots for DMPs. 11](#_Toc226831049)

[Figure S5. GO term Revigo clusters (http://revigo.irb.hr) of enriched pathways for the total sample EWAS.(Supek et al., 2011) 17](#_Toc226831050)

[Blood-brain correlations 19](#_Toc226831051)

[Correlations in BECon database 19](#_Toc226831052)

[Figure S6: CpG variability of the identified sites. 20](#_Toc226831053)

[Figure S7: Correlation plots of the DMPs in relevant Brodmann regions 21](#_Toc226831054)

[Figure S8: Variance brain plot of the DMPs in relevant Brodmann regions 22](#_Toc226831055)

[Blood Brain Comparison Hannon et al. tool (Hannon et al., 2015) 22](#_Toc226831056)

[Figure S9: cg13876222. 23](#_Toc226831057)

[Figure S10: cg05508862. 24](#_Toc226831058)

[Figure S11: cg15349696. 25](#_Toc226831059)

[Figure S12: cg00565090. 26](#_Toc226831060)

[Funding: 27](#_Toc226831061)

[References 29](#_Toc226831062)

# Samples Description

## BIPOGENT-IPM

In the BIPOGENT-IPM cohort, patients with bipolar disorder, who fulfilled the study criteria,^1^ were recruited and evaluated at the Hospital Universitari Institut Pere Mata centers in the Tarragona province in Spain. Diagnosis was established using the Statistical Manual of Mental Disorders, fourth edition (DSM-IV). Healthy controls were contacted through advertisements and study dissemination in the same area as patients and evaluated by the same professionals as patients. All participants provided written informed consent, and the study protocol was approved by the IISPV Ethics Committee (24/09/2015).

Peripheral blood was collected from participants in EDTAK3-containing tubes, and the buffy coat was separated, washed 3 times with phosphate-buffered saline, and stored in a freezer. Genomic DNA was extracted with the Gentra Puregene Blood Kit (Qiagen) including treatment with proteinase K according to the manufacturer’s instructions. DNA concentration was measured by fluorescence using a commercial kit (Sigma Aldrich).

## FOR2107

FOR2107 is a longitudinal cohort study aiming to integrate clinical and neurobiological associations of genetic and environmental risk factors and their interaction involved in the etiology, onset and course of affective disorders.^2^ Participants of the present study were part of the bi-center “Marburg Münster Affective Disorders Cohort Study” (MACS) and were recruited from in and out-patient departments of the universities of Marburg and Münster, Germany, local psychiatric hospitals (Vitos Marburg, Gießen, Herborn, and Haina, LWL Münster, Germany), and via postings in local newspapers and flyers. Lifetime bipolar disorder was assessed using the semi-structured SCID-I interview according to DSM-IV-TR (Diagnostic and Statistical Manual of Mental Disorders) (Wittchen et al., 1997, https://doi.org/10.1026//0084-5345.28.1.68) applied by trained staff. All procedures were approved by the local Ethics Committees of Marburg (AZ:07/14) and Münster (AZ:2014-422-b-S), Germany according to the Declaration of Helsinki. Participants gave written informed consent prior study participation and received financial compensation. DNA methylation was profiled using the Infinium MethylationEPIC v1.0 BeadChip (Illumina, San Diego, CA, USA) based on DNA extracted from whole blood samples.

## HALIFAX-CAGLIARI

Halifax-Cagliari cohort, is a study that enrolled two cohorts from Canada and Italy.^3-6^ For the Halifax part, participants with bipolar disorder were recruited from patients longitudinally followed at specialty mood disorders clinic in Halifax and from the Maritime Bipolar Registry (Canada). All cases were interviewed in a blind fashion with the Schedule of Affective Disorders and Schizophrenia-Lifetime version (SADS-L) by pairs of clinical researchers (psychiatrists and/or trained research nurses). Additional clinical details were extracted from hospital records. All interviews and case notes were presented to a panel of senior clinical researchers to reach consensus diagnosis according to DSM-IV Criteria and Research Diagnostic Criteria (RDC). Protocols and procedures were approved by the local Ethics Committee at Nova Scotia Health Authority (REB FILE #: 1020604), and written informed consent was obtained from all patients before participation in the study

 For the Cagliari part, patients were recruited at the Clinical Psychopharmacology Centre, University Hospital Agency of Cagliari, Italy, the Unit of Clinical Psychiatry of the University Hospital of Cagliari, Italy, and at the Mood Disorder Lucio Bini Center, Cagliari (Italy). Patients were enrolled in the genetic study if they met the following inclusion criteria: diagnosis of either bipolar type I or bipolar type II disorder according to DSM-5 criteria using the Italian version of the SCID-5-CV (Structured Clinical Interview for DSM-5 Clinical Version) and being in euthymic phase. Exclusion criteria included diagnosis of any eating disorders, post-traumatic stress disorder, substance use disorders, neurological disorders, traumatic brain injury, or severe medical conditions. For an accurate assessment of treatment response, we used all available information including data from clinical records, diagnostic interviews, and prospective follow-up assessed by NIMH Life- Chart Method. The Retrospective Criteria of Long-Term Treatment Response in Research Subjects with Bipolar Disorder scale score criteria (Alda scale) were used to assess lithium response, which has a range of 0 to 10, with scores of 7 and higher considered a good response.

Healthy controls were recruited based on the same exclusion criteria described for patients and had no personal or familial history of psychiatric disorders in first degree relatives. Controls were administered the Italian version of the SCID-I/NP 26 to rule out the presence of Axis I psychiatric disorders.

The research protocol followed the principles of the Declaration of Helsinki and was approved by the Ethics Committee of the University of Cagliari, Italy (approval number: 348/FC/2013 and PG/2018/11,693). All individuals provided a written consent form regarding the use of their biological and clinical data for research purposes. Blood samples were gathered at the beginning of the study along with the relevant demographic and biometrical data. DNA was isolated from the buffy coat using the DNeasy Mini Kit (Qiagen) or the salting out method and stored at -80.

## Imaging Genetics in Psychosis

The Imaging Genetics in Psychosis study is a cross-sectional study comprising individuals with bipolar-I disorder or schizophrenia,^7^ in addition to a sample of healthy controls. Participants were recruited from the Greater Sydney area in New South Wales, Australia. Clinical cases were recruited either from outpatient services of the South Eastern Sydney Illawarra Area Health Service (SESIAHS), the Australian Schizophrenia Research Bank,^8^ and the Sydney Bipolar Disorders Clinic.^9^ Healthy controls were recruited from the local community. Cases were required to meet ICD-10 criteria for schizophrenia/schizoaffective disorder (the “schizophrenia” group) or bipolar-I disorder. Participants were excluded if they had a current neurological disorder, currently met diagnostic criteria for substance abuse or dependence, or had received electroconvulsive treatment in the past six months. Additional exclusion criteria for the control group included if participants had a lifetime history of a DSM-IV Axis-I disorder or a history of psychotic disorders among first-degree biological relatives. Only bipolar disorder patients and healthy controls were included in the current study. DNA methylation quantification was performed on blood or ficoll-treated blood samples using Illumina 450K BeadChip (Illumina, San Diego, CA, USA), with the EZ-96 DNA Methylation kit (Zymo Research, Orange, CA, USA) used to complete bisulfite conversions [see Watkeys et al., (2020) for full details - 10.1016/j.pnpbp.2020.109925]. All participants provided informed consent, and this study was conducted in accordance with the Declaration of Helsinki with ethical approval provided by the UNSW Human Research Ethics committees (HC12384), St. Vincent’s Hospital (HREC/10/SVH/9), and the South East Sydney and Illawarra Area Health Service (HREC 09/081).

## PMDH

BPD cases and control samples from the same methylation chips were selected from the Post-Deployment Mental Health (PDMH) study, a multi-site study of U.S. Afghanistan and Iraq era veterans conducted by the U.S. Department of Veterans Affairs (VA) Mid-Atlantic Mental Illness Research, Education, and Clinical Center (MIRECC).^10^ Participants were recruited at four VA hospitals located in the Southeastern U.S through mailings, advertisements, and clinician referrals. The study protocol was approved by each VA hospital’s local institutional review board (#1596360) and written informed consent was obtained from all participants prior to enrollment. BPD status was determined via the Structured Clinical Interview for DSM-IV (SCID-IV). DNA was extracted from whole blood and analyzed on the Infinium Methylation EPICv1 Beadchip.

## TOP-TOP+

In the TOP study (Tematisk Område Psykoser), individuals of European ancestry with bipolar disorder (BD) who were born in Norway were recruited from psychiatric hospitals in the Oslo region.^11^ To participate in the current study, subjects needed to be between 18 and 65 years old, obtain an IQ score of above 70, meet the DSM-IV criteria for BD, and be willing and able to provide informed consent. Diagnosis was established using the Structured Clinical Interview for DSM-IV-TR-axis I disorders (SCID-I).

Healthy control subjects were randomly selected from statistical records of individuals residing in the same catchment area as the patient groups. These controls underwent screening through interviews and the Primary Care Evaluation of Mental Disorders (PRIME-MD). Control subjects were excluded if they had a history of moderate to severe head injury, neurological disorders, intellectual disability, or if they fell outside the age range of 18–65 years. Additionally, healthy controls were excluded if they or any close relatives had a lifetime history of severe psychiatric disorders.

All participants provided written informed consent, and the study protocol was approved by the Norwegian Scientific-Ethical Committee and the Norwegian Data Protection Agency (REK #​2009/2485).

DNA was extracted from whole blood. Typing was performed in three phases: TOP1 was typed on EPIC v1 (2016), TOP3 on EPIC v1 (2019), and TOP+ on EPIC v2 (2024). All methylation typing was performed at Life & Brain Genomics, GmBH. The three phases were QC’d and analysed separately.

## UNICA_BD

Patients were recruited at the Clinical Psychopharmacology Centre, University Hospital Agency of Cagliari, Italy, and the Unit of Clinical Psychiatry of the University Hospital of Cagliari, Italy.^3-5^ Patients were enrolled in the genetic study if they met the following inclusion criteria: diagnosis of either Bipolar I or Bipolar II disorder according to DSM-5 criteria using the Italian version of the SCID-5-CV (Structured Clinical Interview for DSM-5 Clinical Version),and being in euthymia. Exclusion criteria were a diagnosis of any eating disorders, post-traumatic stress disorder, substance use disorders, neurological disorders, traumatic brain injury, or severe medical conditions. For an accurate assessment of treatment response, we used all available information including data from clinical records, diagnostic interviews, and prospective follow-up assessed by NIMH Life- Chart Method. The Retrospective Criteria of Long-Term Treatment Response in Research Subjects with Bipolar Disorder scale score criteria (Alda scale) were used to assess lithium response, which has a range of 0 to 10, with scores of 7 and higher considered a good response.

Healthy controls were recruited based on the same exclusion criteria described for patients and had no personal or familial history of psychiatric disorders in first degree relatives. Controls were administered the Italian version of the SCID-I/NP 26 to rule out the presence of Axis I psychiatric disorders.

All individuals provided a written consent form regarding the use of their biological and clinical data for research purposes. Blood samples were gathered at the beginning of the study along with the relevant demographic and biometrical data. DNA was isolated from the buffy coat using the DNeasy Mini Kit (Qiagen) or the salting out method, and stored at -80.

The research protocol followed the principles of the Declaration of Helsinki and was approved by the Ethics Committee of the University of Cagliari, Italy (approval number: 348/FC/2013 and PG/2018/11,693).

## UNSW Bipolar Kids & Sibs

Recruitment area: Participants were recruited from the greater Sydney area of New South Wales, Australia, and were predominantly of European ancestral descent.^12,13^

Method of recruitment (cases and controls): Participants were aged 12–30 years and were recruited from families who had previously participated in BD family studies, specialised BD research clinics, mental health consumer organisations, or in response to public notices via print/electronic media, and noticeboards in universities and local communities. Written informed consent was obtained from all participants, with additional parental consent for participants aged <16 years.

Diagnosis criteria: BD cases met DSM-IV criteria for BD type-I (BD-I) or type-II (BD-II). Control participants had no personal or familial (first-degree) history of BD-I, BD-II, recurrent unipolar disorder, SABP, schizophrenia, recurrent substance abuse or psychiatric hospitalisation, and no second-degree relative with a past mood-disorder hospitalisation or history of psychosis.

Ethical approval: This study was approved by University of New South Wales Human Research Ethics Committee (HREC Protocol 09/097).

DNA source: Peripheral blood - whole blood or ficoll separated blood cells

DNA extraction and other information on sample processing: Peripheral blood samples were collected in EDTA tubes for processing and DNA extraction. Samples were processed either as: 1) whole blood, or 2) ficoll separated blood cells (where the extraction of DNA and establishment of a transformed cell line was required from a single collection tube). For the latter, blood was diluted with saline, then centrifuged through a ficoll layer to isolate lymphocytes for EBV transformation. The leftover ficoll layer cells (i.e. red and white cells from the ficoll/PBS/plasma layer, including pelleted cells and granulocytes) was processed for DNA extraction. DNA was extracted by Genetic Repositories Australia, using either the Qiagen Autopure® LS or a Puregene salting out methodology (Qiagen, Chadstone, Victoria, Australia), according to manufacturers’ instructions. Participants meeting inclusion criteria for this study were selected from methylation quantification performed across two batches using the Illumina MethylEPICv1 BeadChip.

## UTHealth Houston

One hundred and sixty-three adults with BD and 76 non-psychiatric controls were recruited at the Center of Excellence in Mood Disorders, Houston, TX. BD diagnosis was ascertained in the Structured Clinical Interview for DSM-IV Axis I Disorders (SCID-I).^14^ Other demographic and clinical characteristics (e.g., substance use, previous hospitalizations, psychiatric comorbidities) were obtained by demographic questionnaire and clinical interview. Interviews were administered by trained evaluators and reviewed by a board-certified psychiatrist. Young Mania Rating Scale (YMRS) and Montgomery-Asberg Depression Rating Scale (MADRS) were administered for assessing manic and depressive symptomatology. Exclusion criteria for all participants included neurological disorders and traumatic brain injury, schizophrenia, developmental disorders, eating disorders, intellectual disability, and recent illicit drug use by urine drug screen. Exclusion criteria for controls included a history of any Axis I disorder in first-degree relatives or if they had taken prescribed psychotropic medication at any point in their lives. The study protocol was approved by the local institutional review board (IRB), approval #HSC-MS-09-0340, and informed consent was obtained from all participants at enrolment and before any procedure.

All participants provided peripheral blood by venipuncture, which was stored in EDTA-containing vacutainers at −80 degrees Celsius. DNA was isolated from the buffy coat using the DNeasy Blood & Tissue Mini Kit (Qiagen, Hilden, Germany). Five hundred nanograms of DNA were bisulfite-converted using the EZ DNA Methylation Kit (Zymo Research, Irvine, CA, USA). Genome-wide DNA methylation was measured using the Infinium EPICMethylation BeadChip version 1.0 (Illumina, San Diego, CA, USA), according to the manufacturer’s instructions.

# Supplementary Figures

## Figure S1(A-D): QQ plots for sex-adjusted EWAS samples.

| 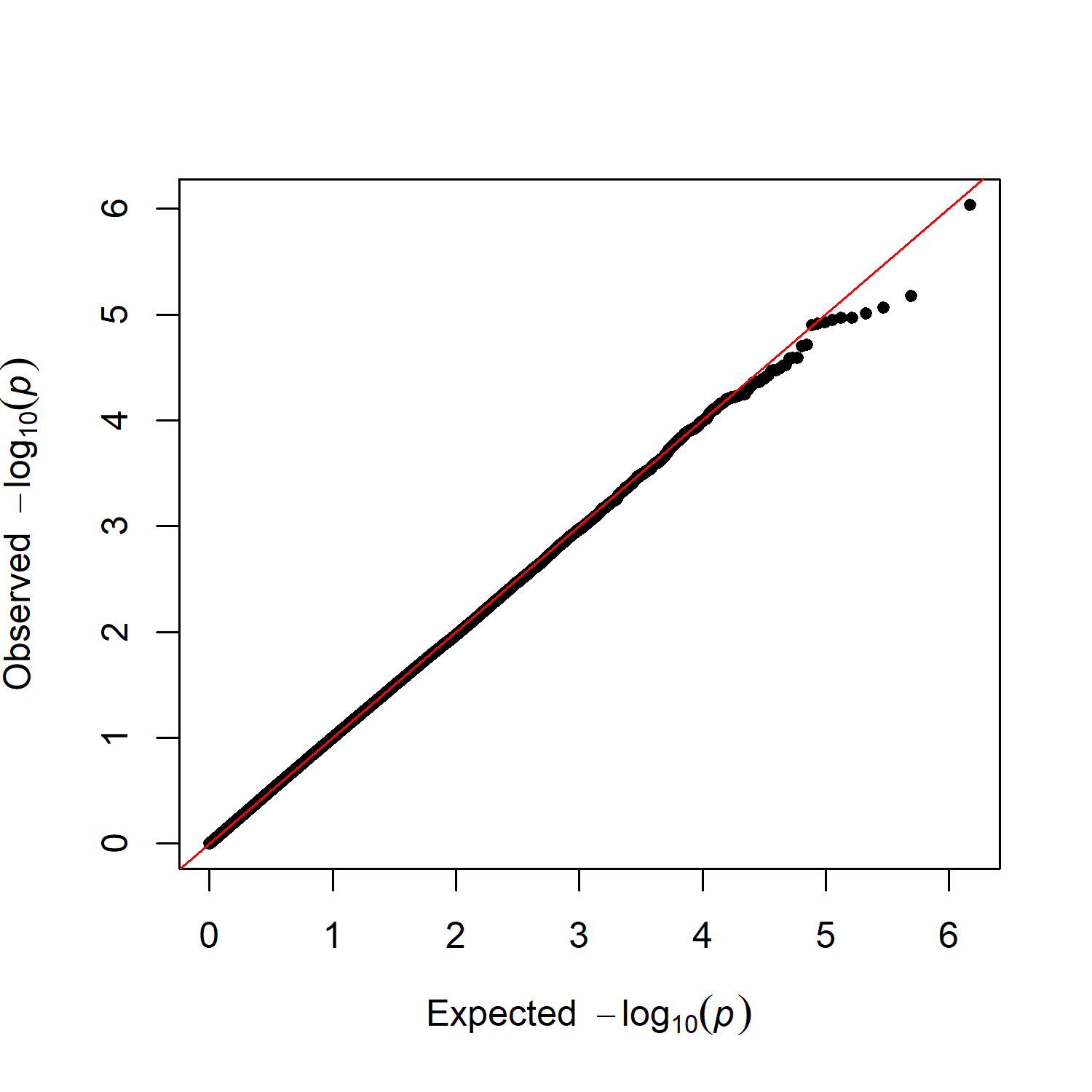 | 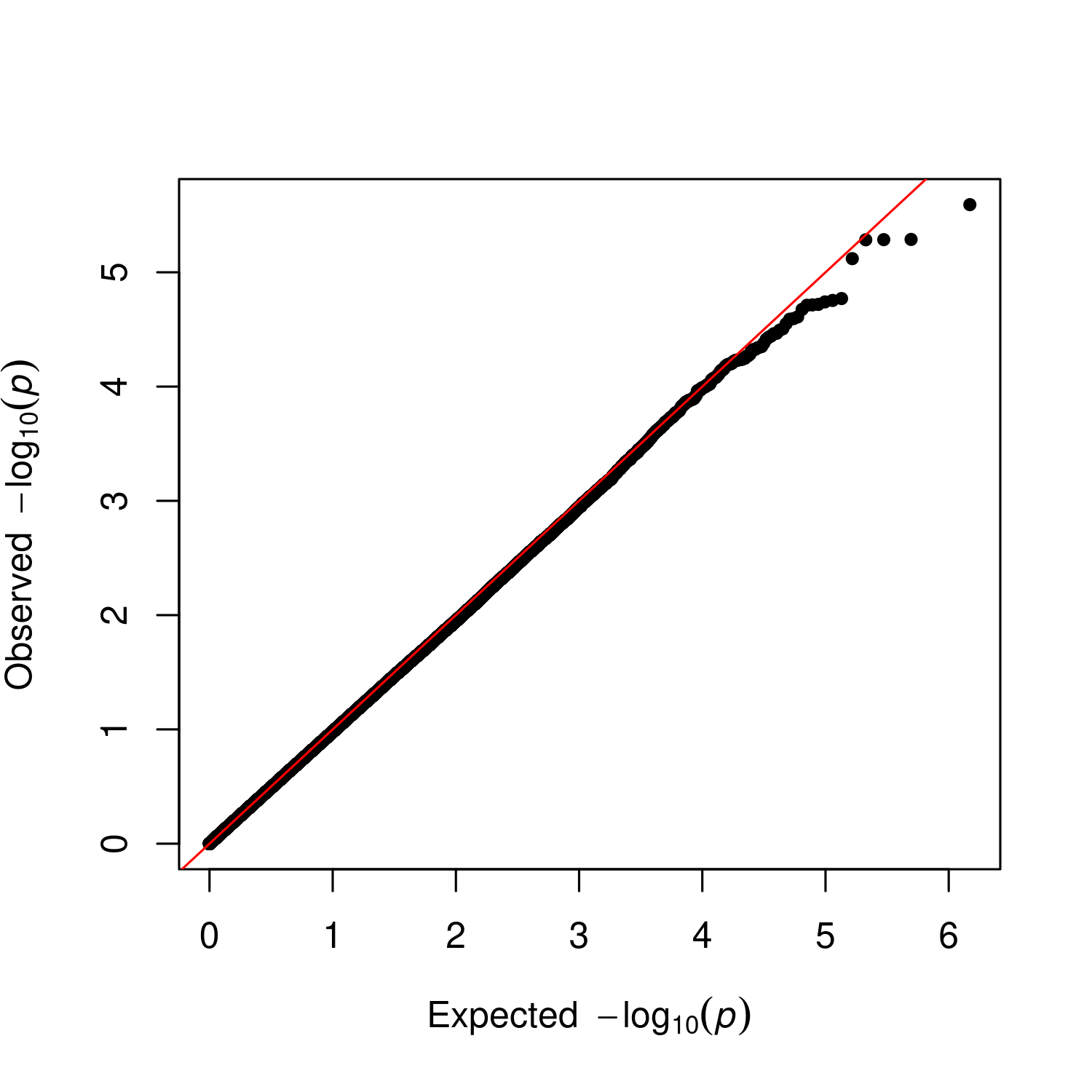 |
| --- | --- |
| 1. UNSW_BipolarKids&Sibs | 1. PDMH |
| 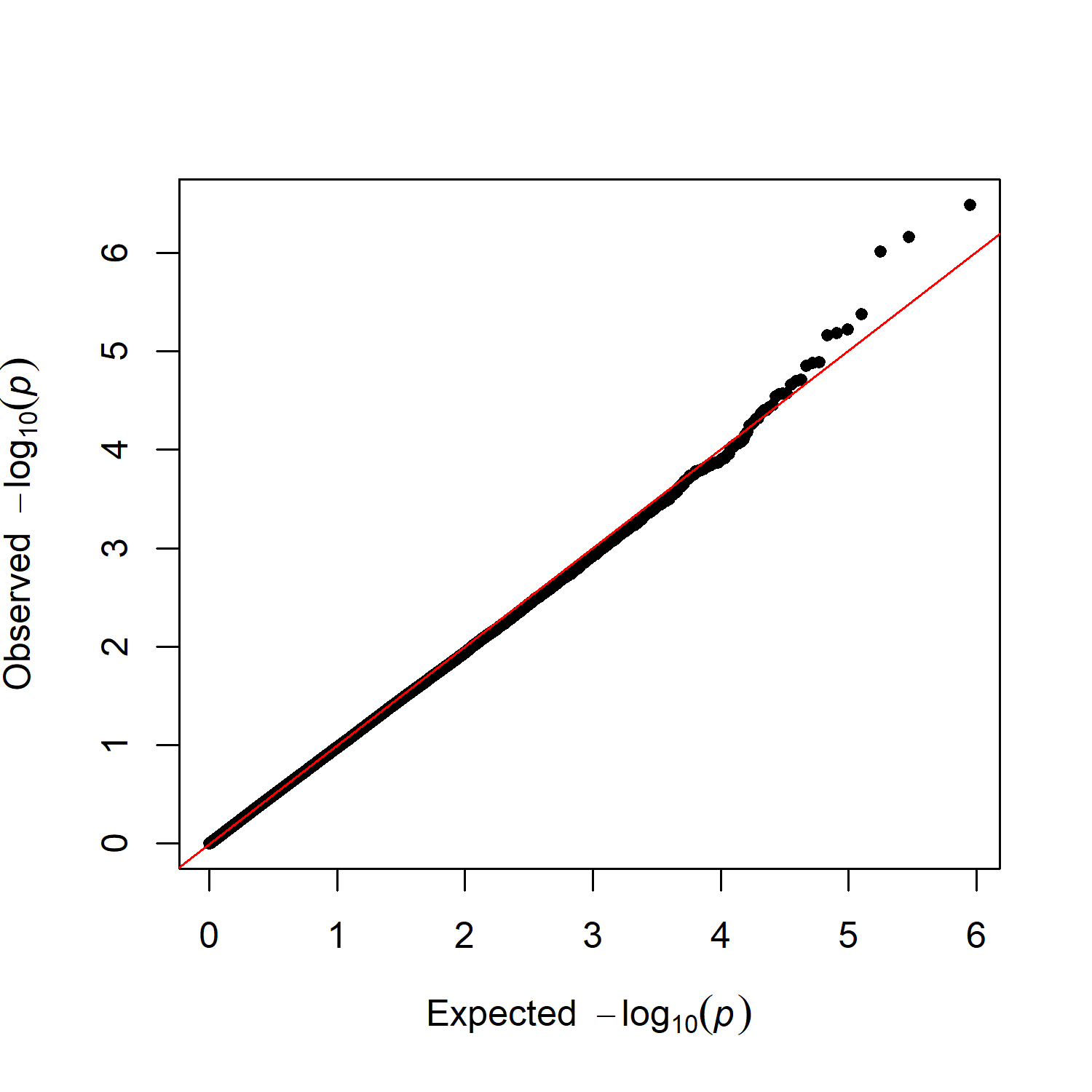 | 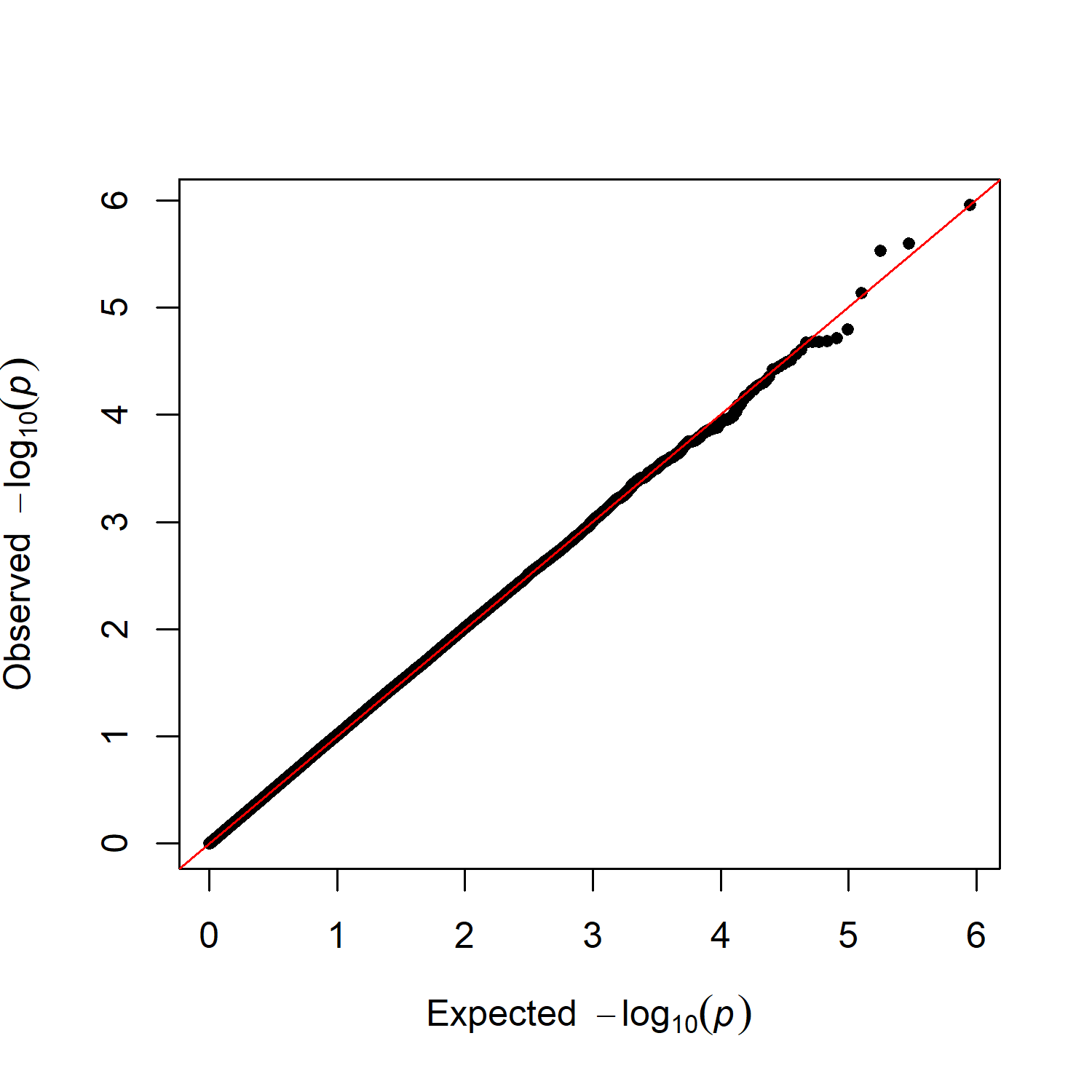 |
| 1. IGP - ficoll | 1. IGP – whole blood |
|  | |

## Figure S2 (A-H): QQ plots for female-only EWAS samples.

| 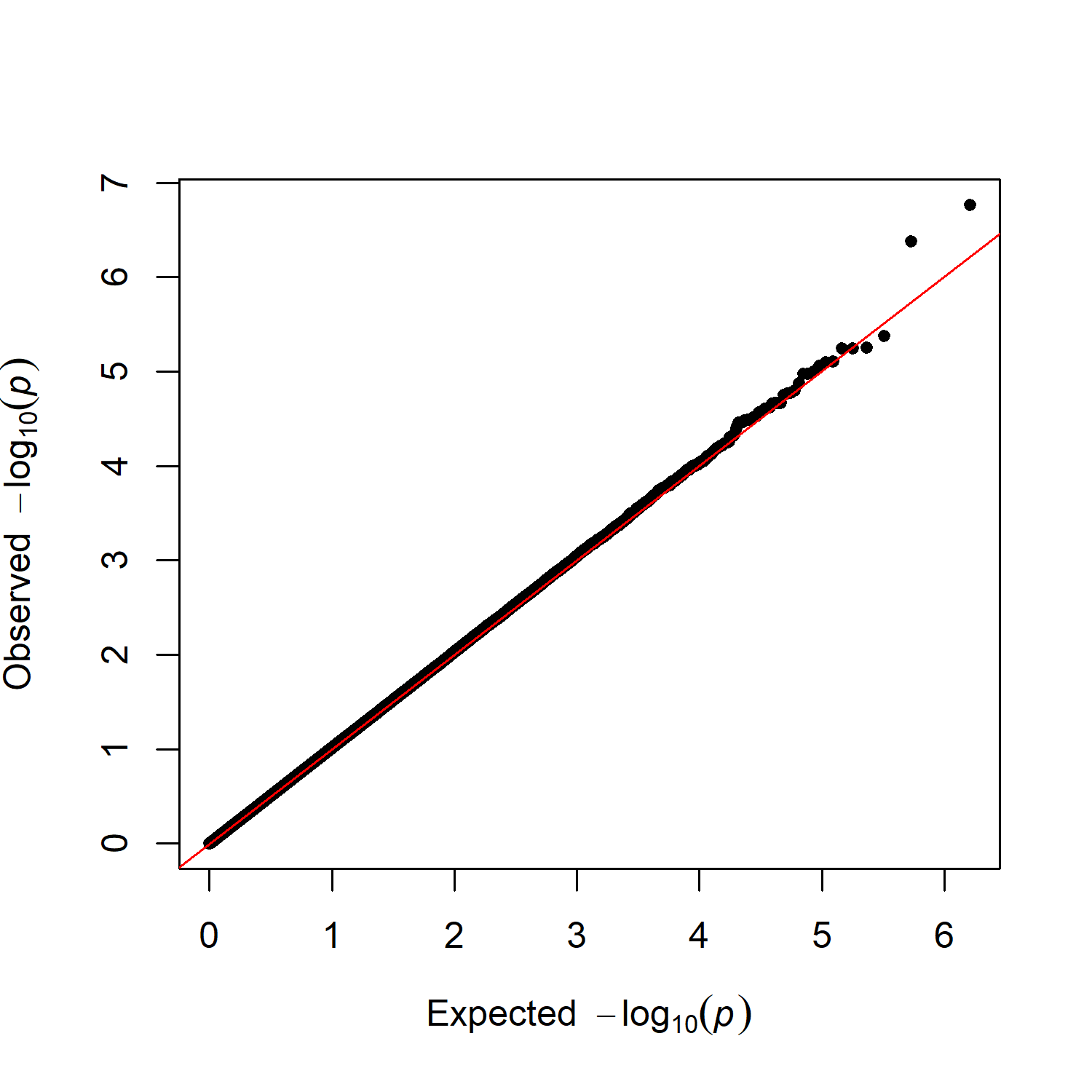 | 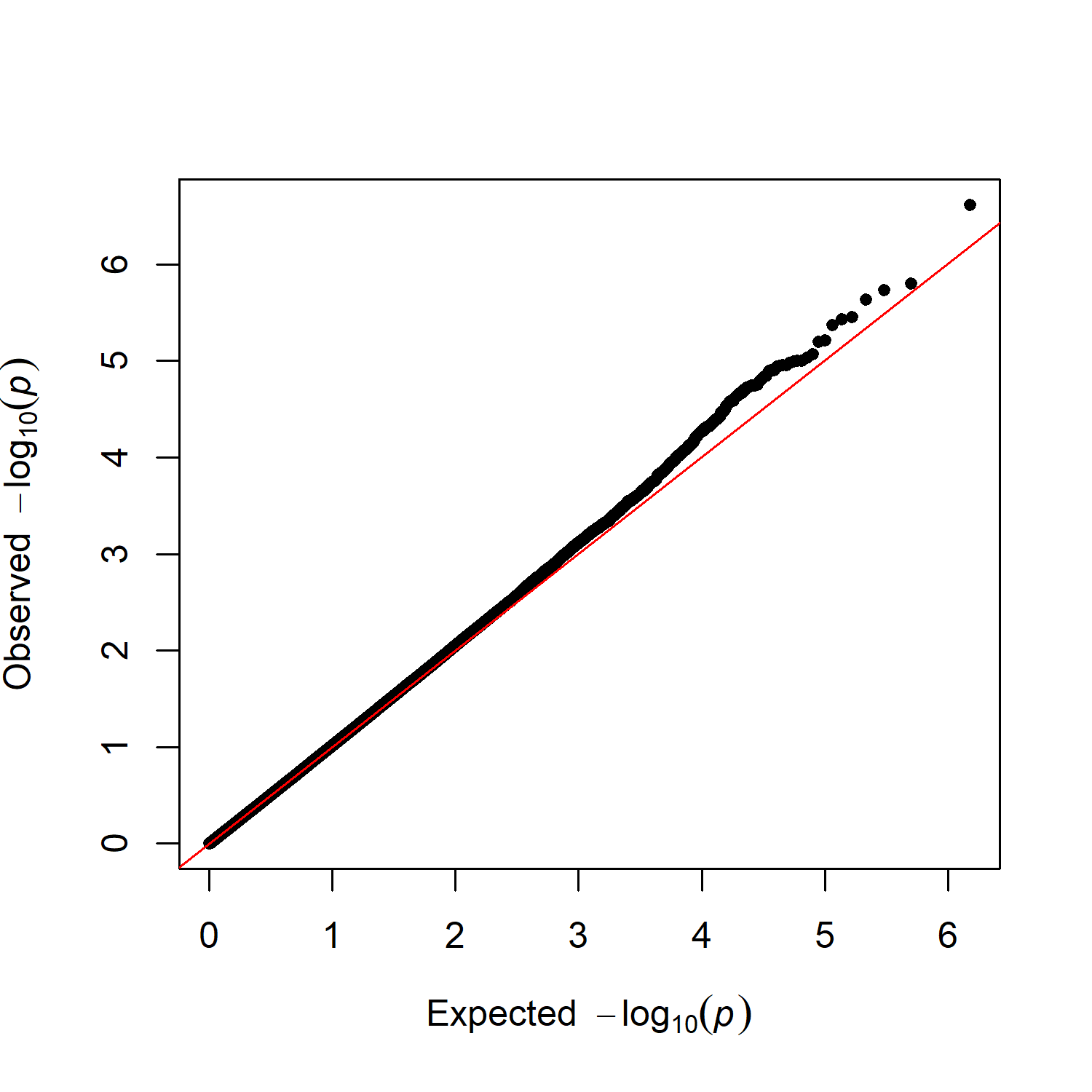 | 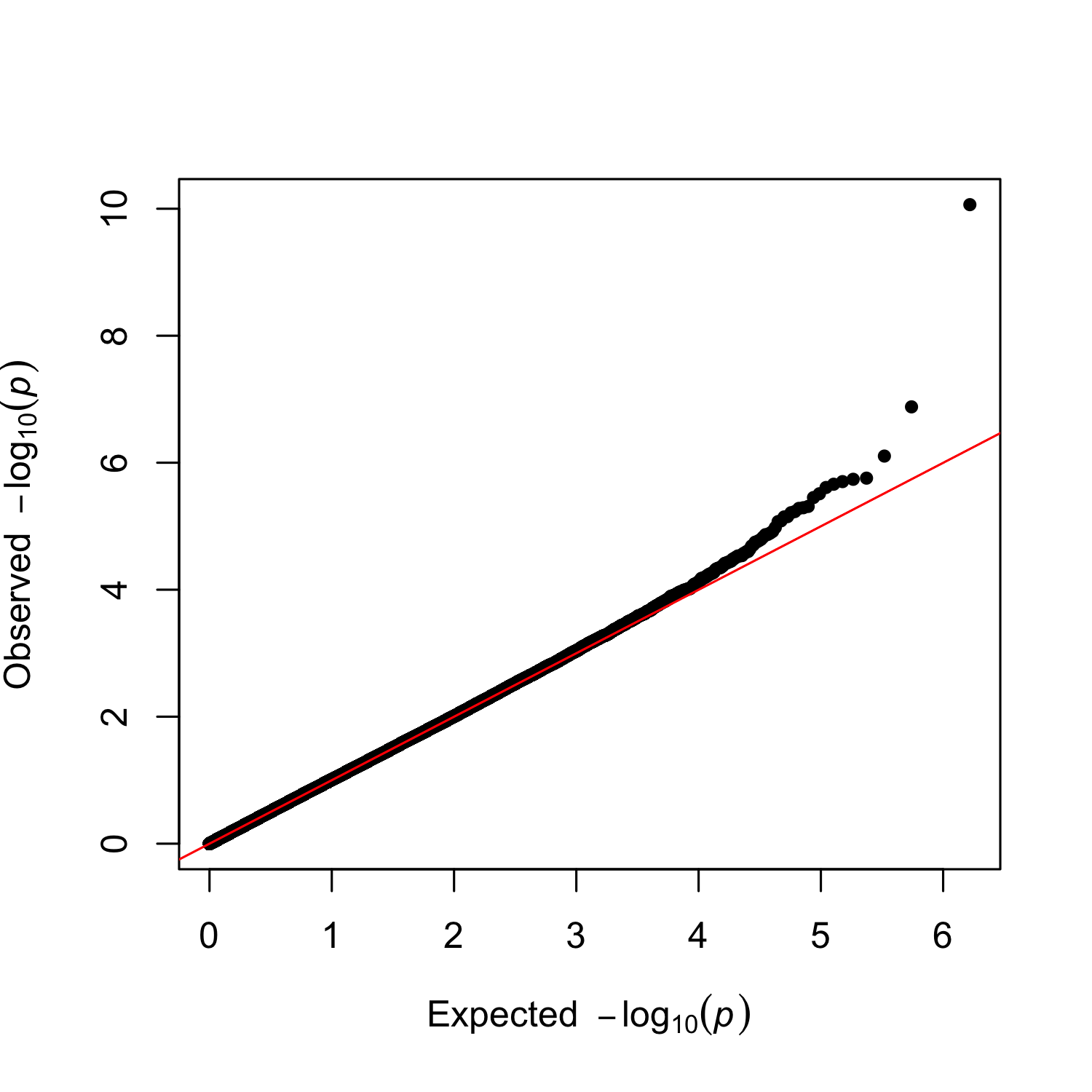 | 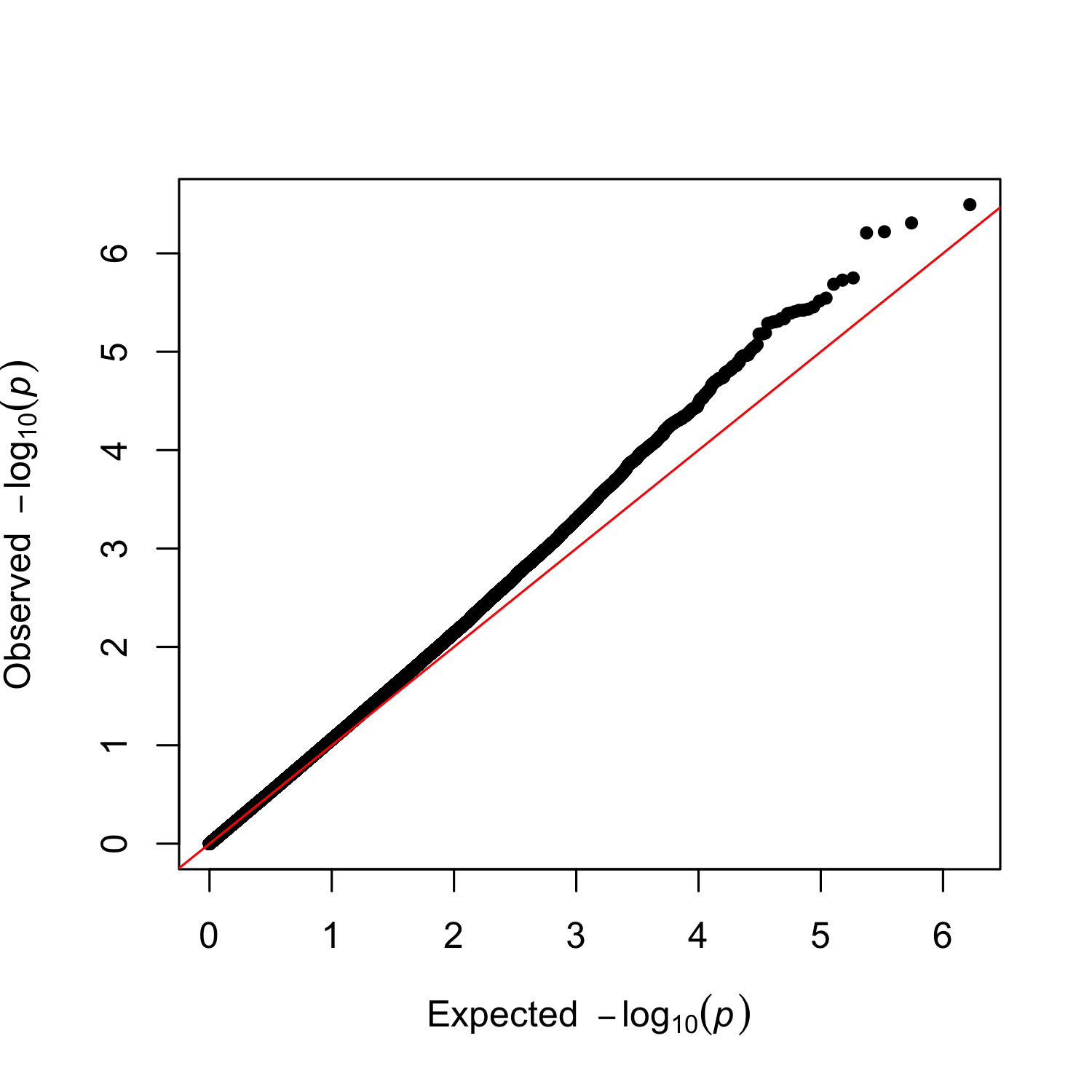 |
| --- | --- | --- | --- |
| 1. UNICA | 1. FOR2017 | 1. Halifax-Cagliari | 1. BIPOGENT-IPM |
| 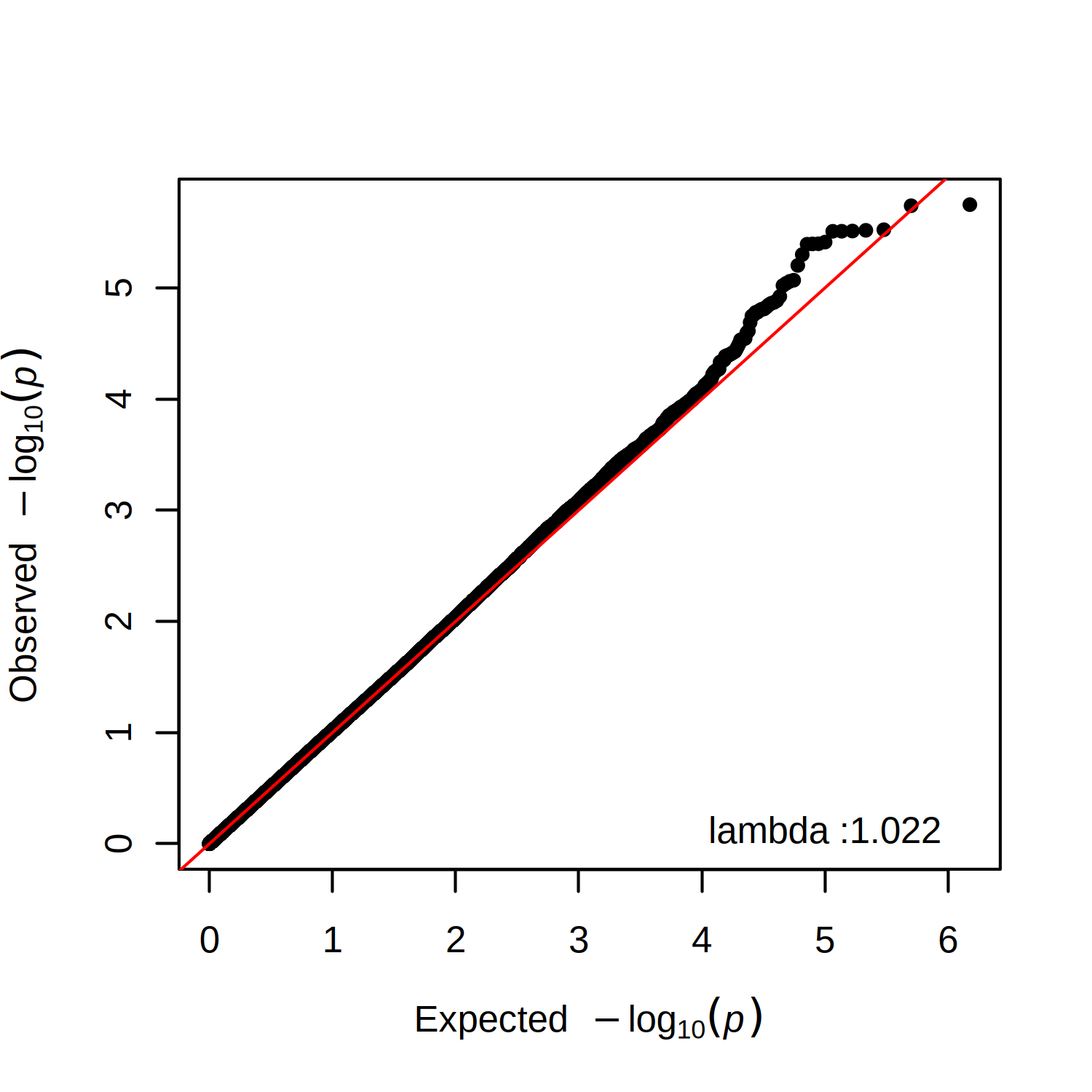 | 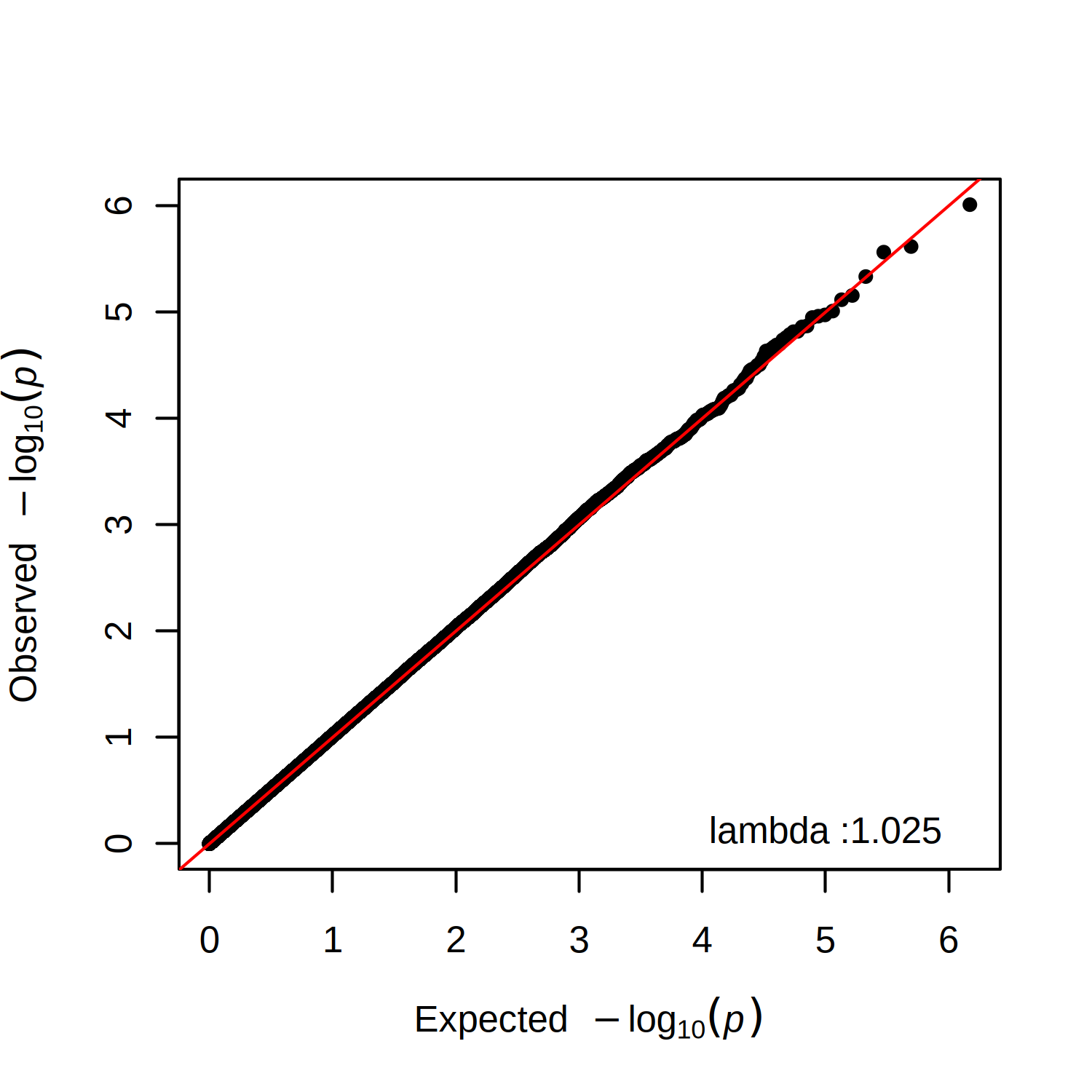 | 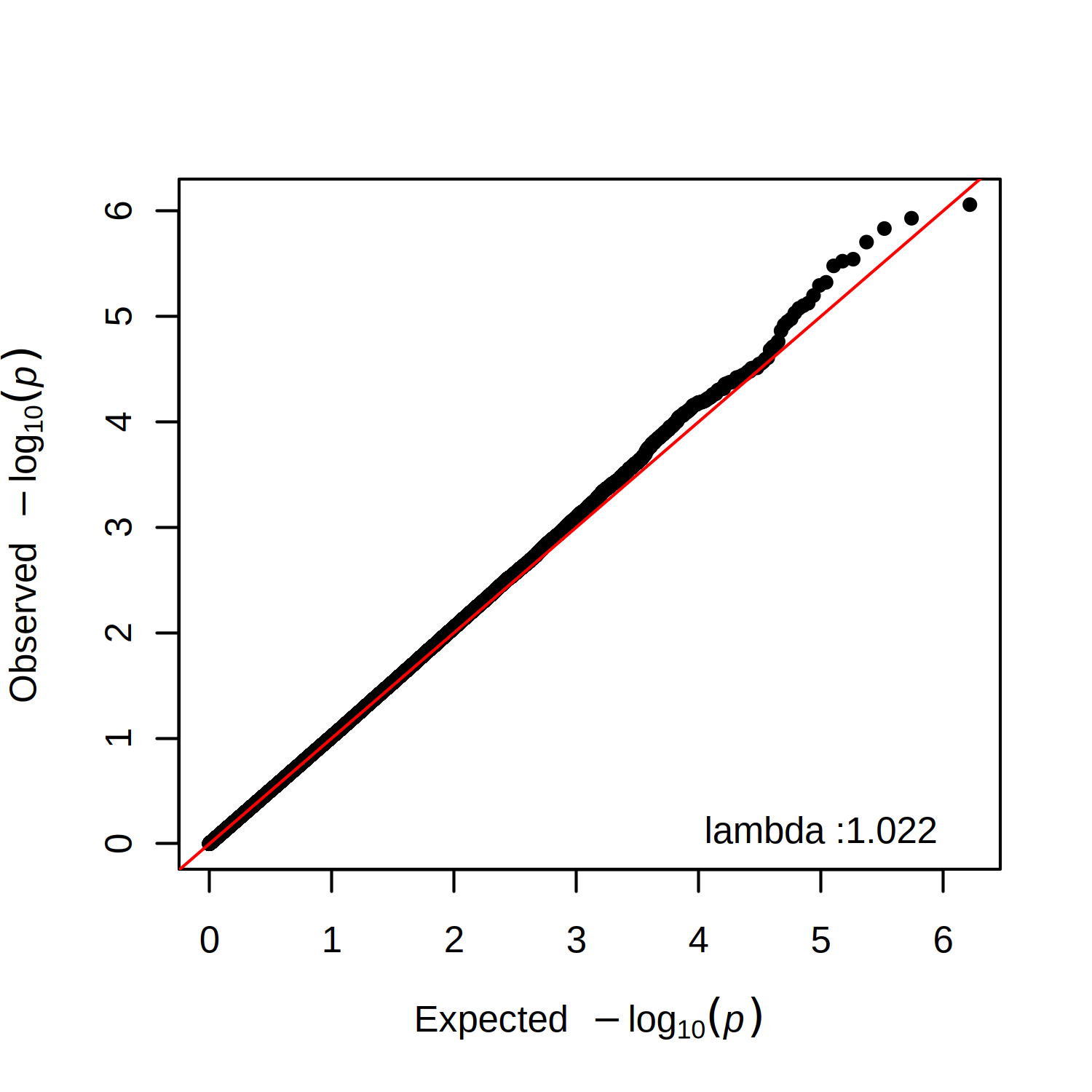 | 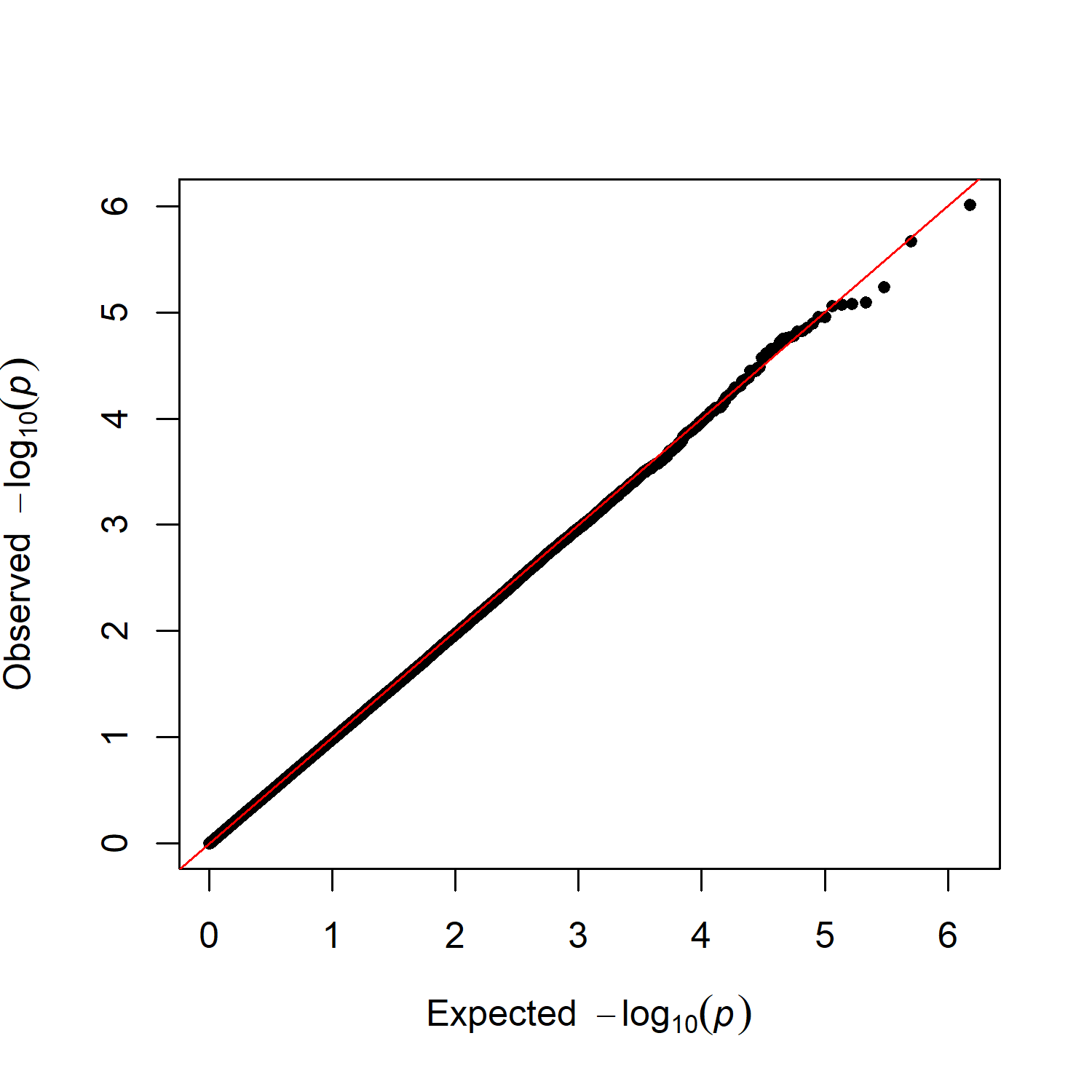 |
| 1. TOP1 | 1. TOP3 | 1. TOP+ | 1. UTHealth-Houston |
|  | | | |

| Figure S3 (A-I): QQ plots for male-only EWAS samples. |
| --- |

| 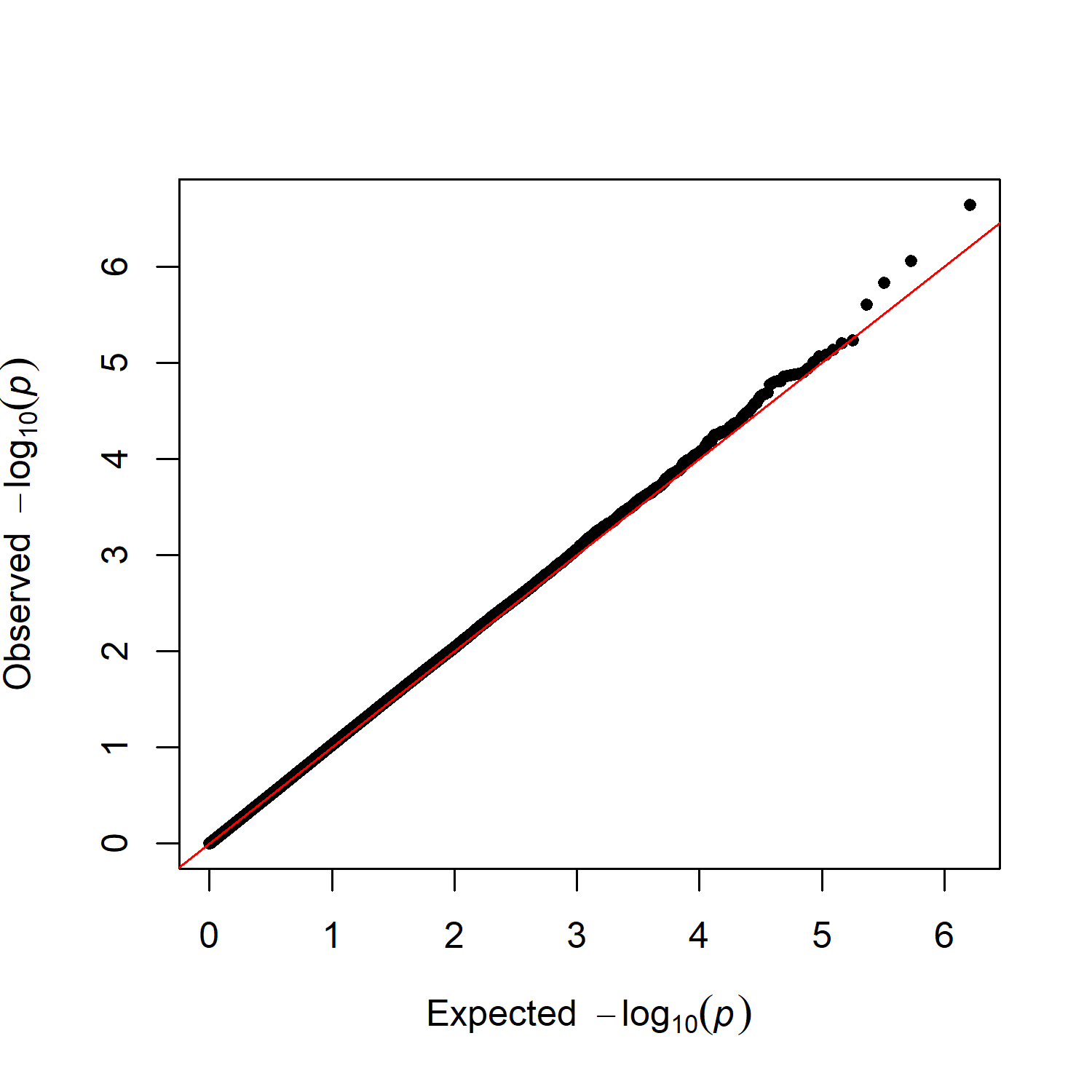 | 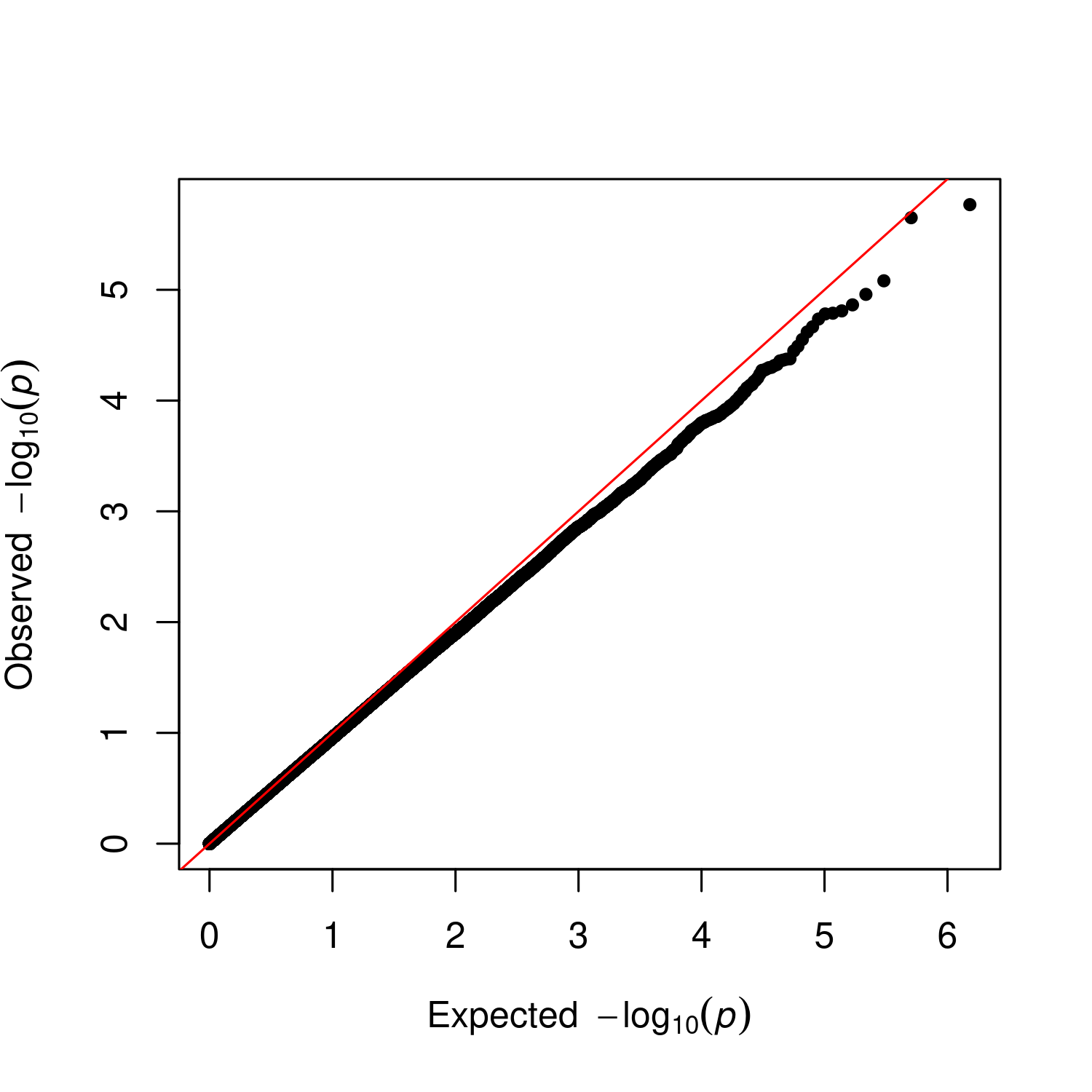 | 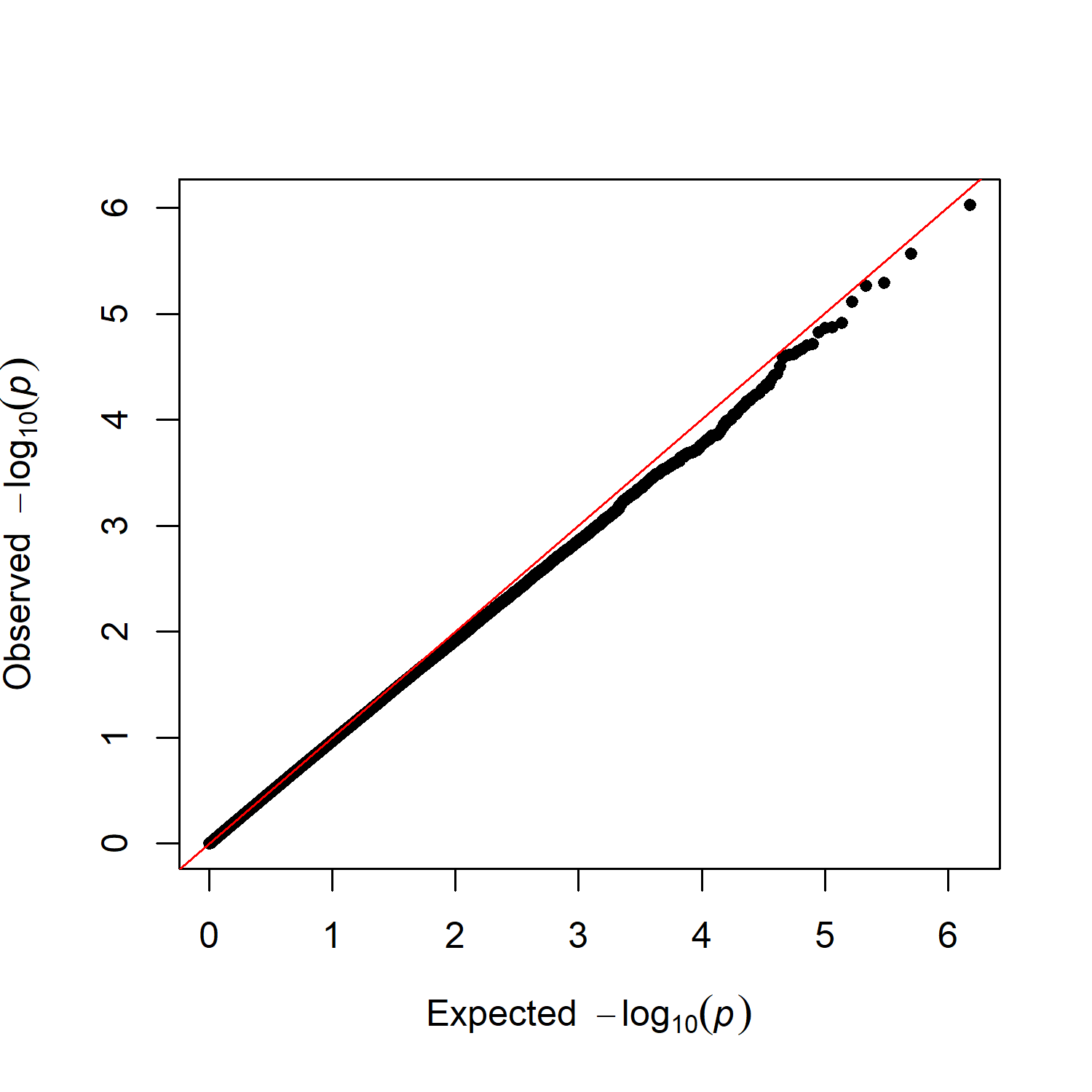 |
| --- | --- | --- |
| 1. UNICA | 1. PDMH | 1. FOR2017 |
| 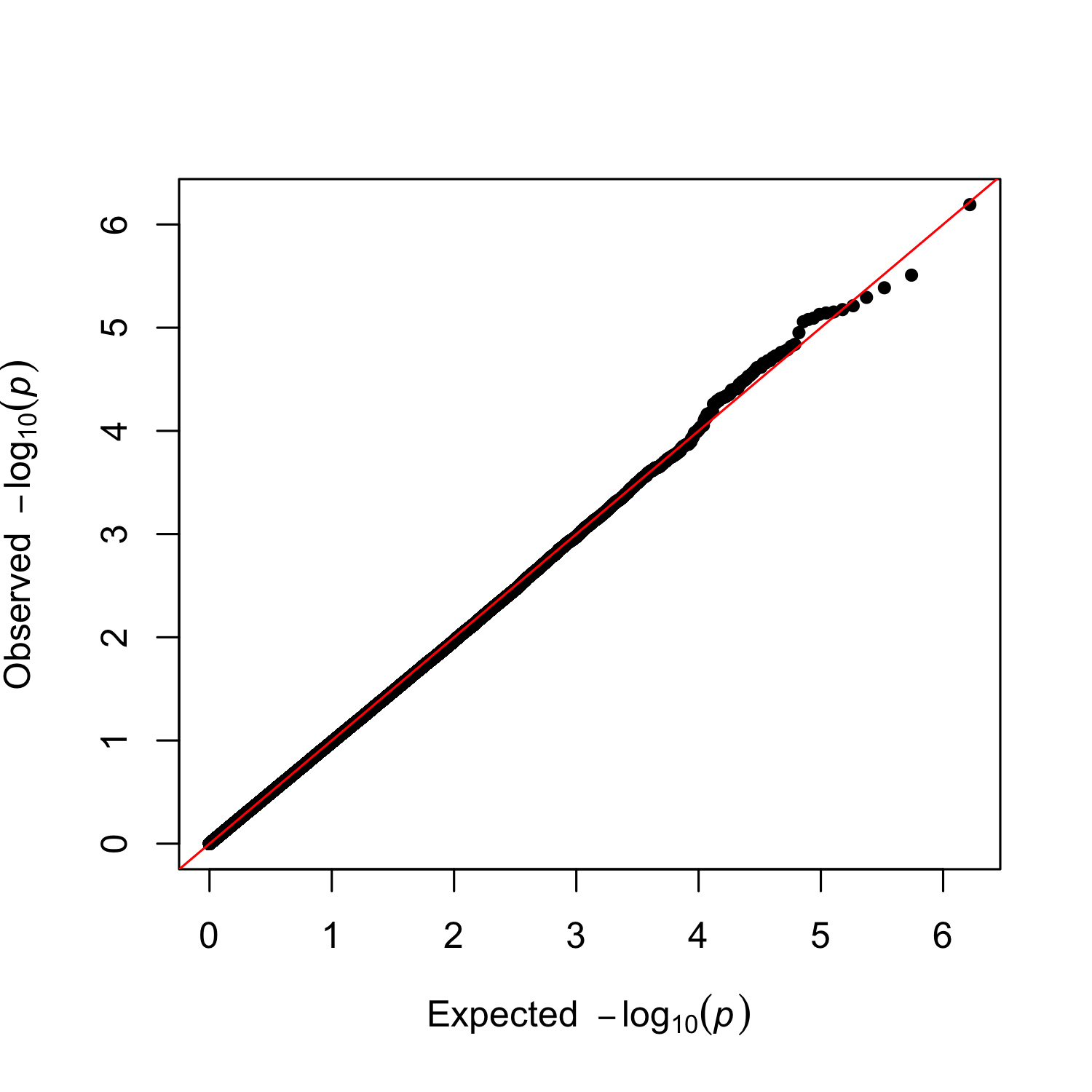 | 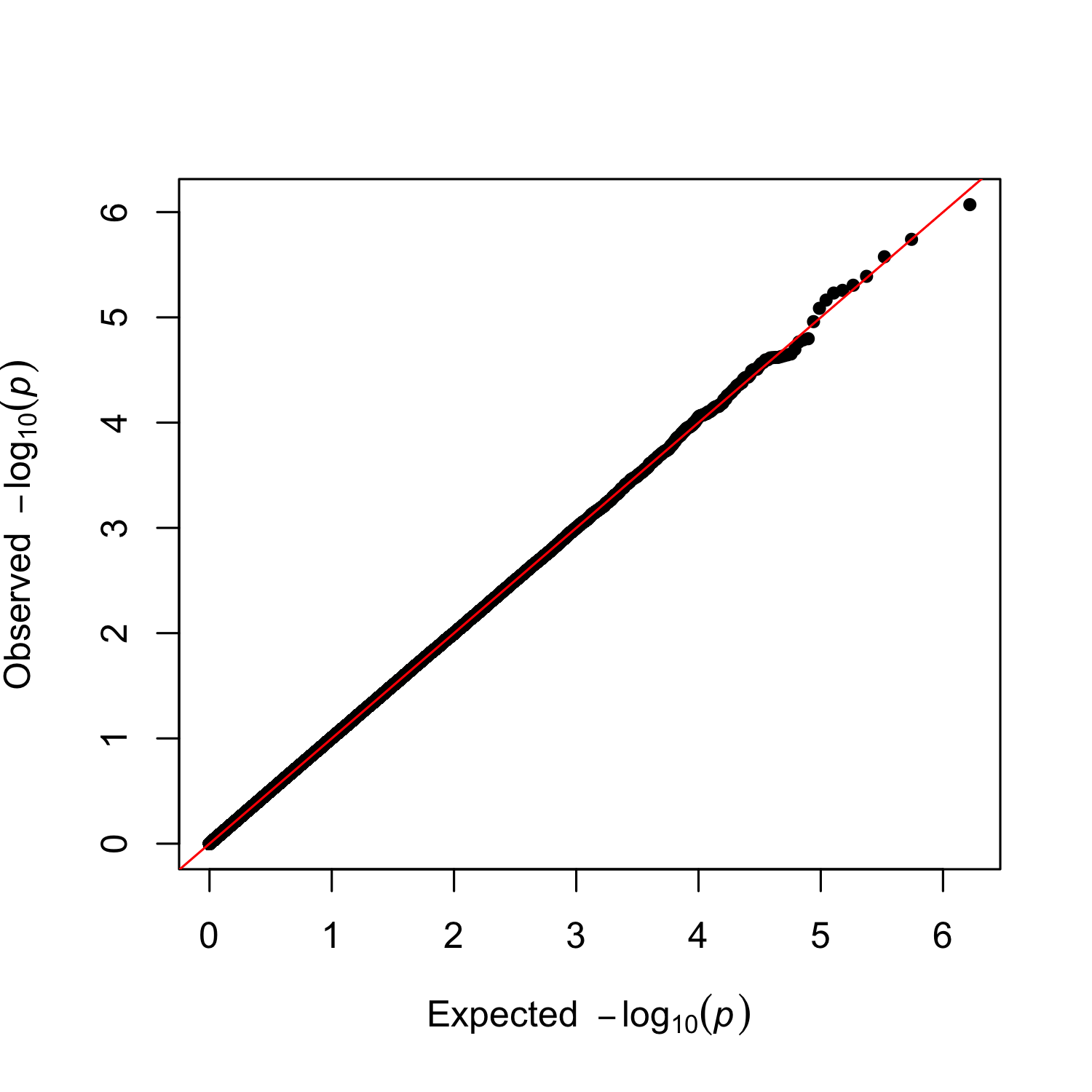 | 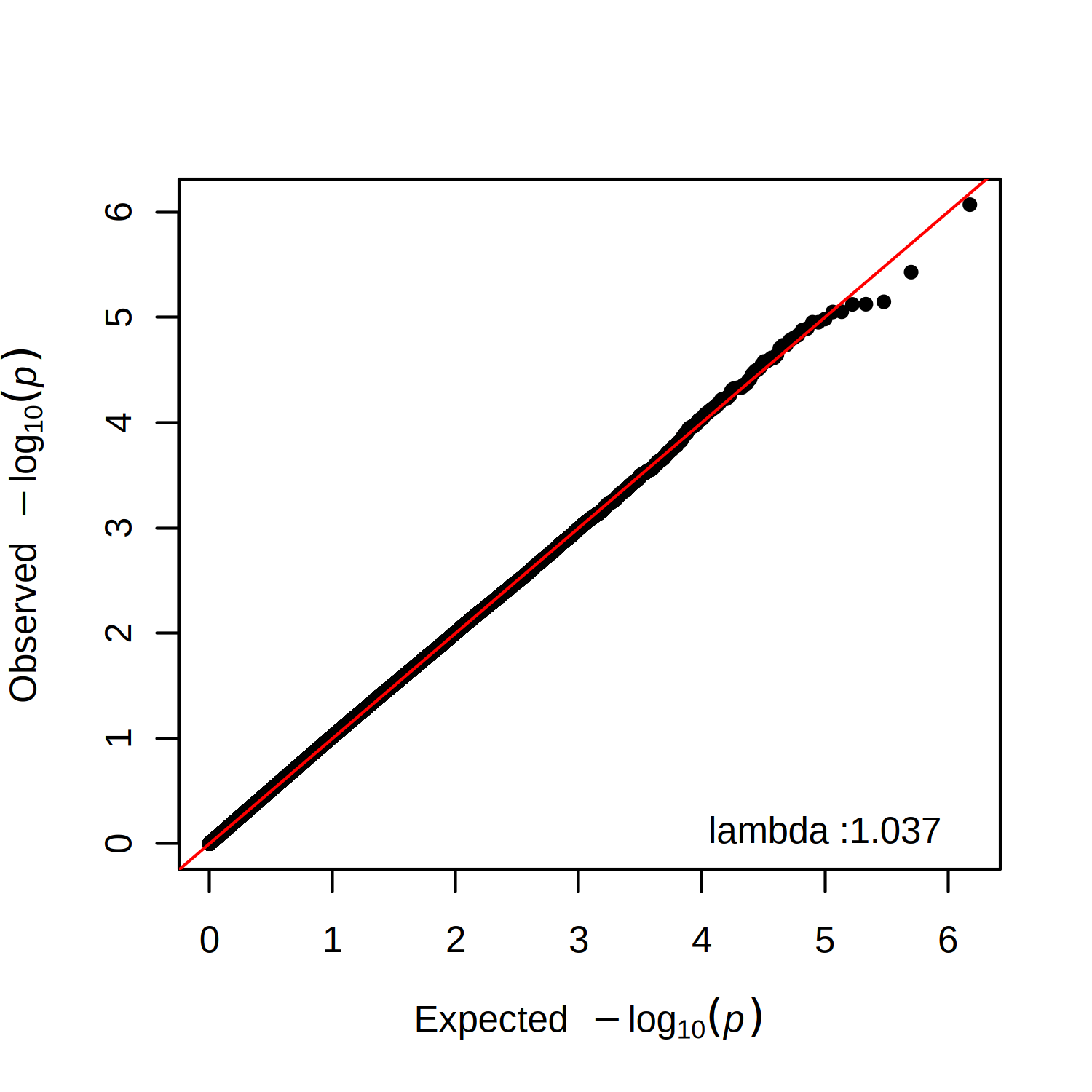 |
| 1. Halifax-Cagliari | 1. BIPOGENT-IPM | 1. TOP1 |
| 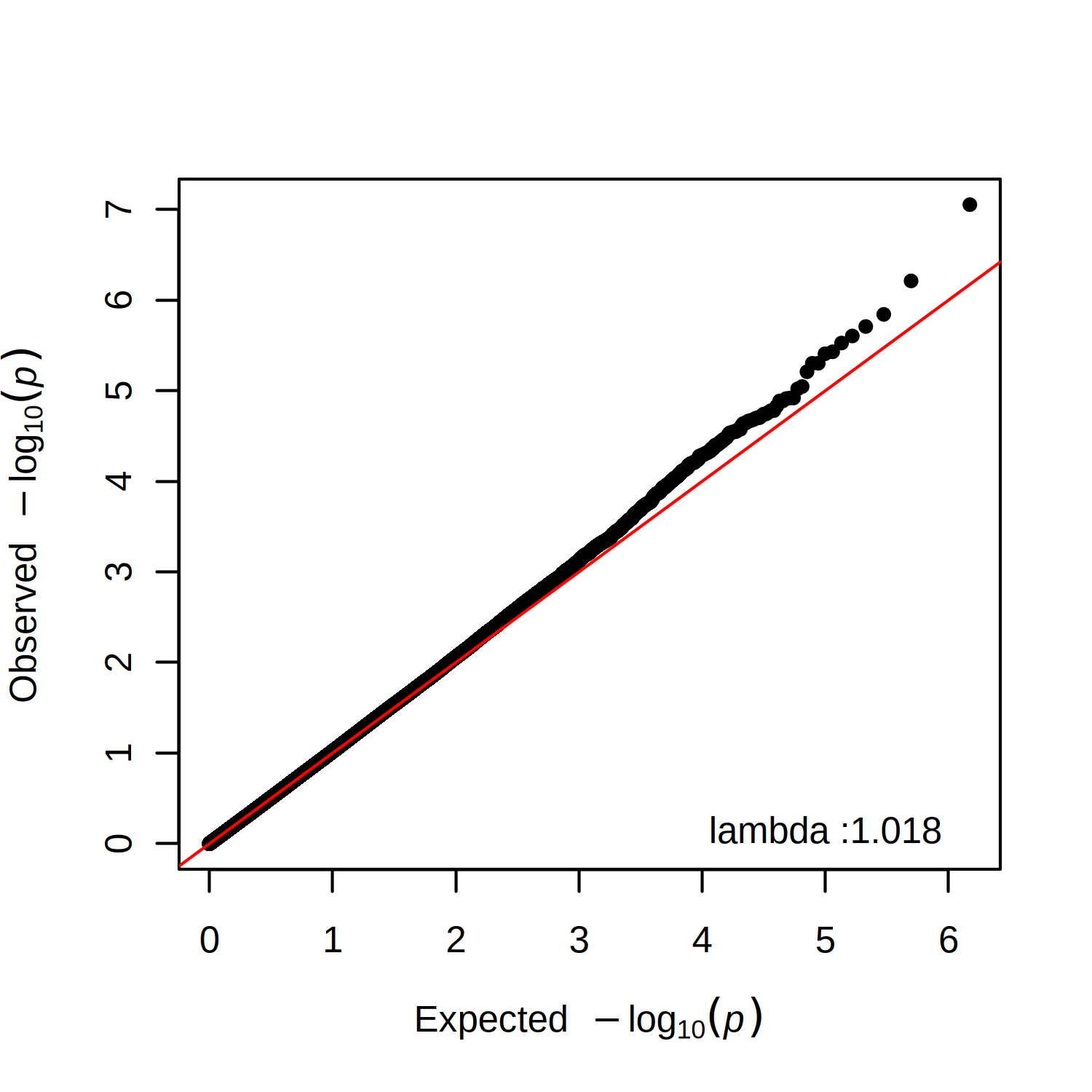 | 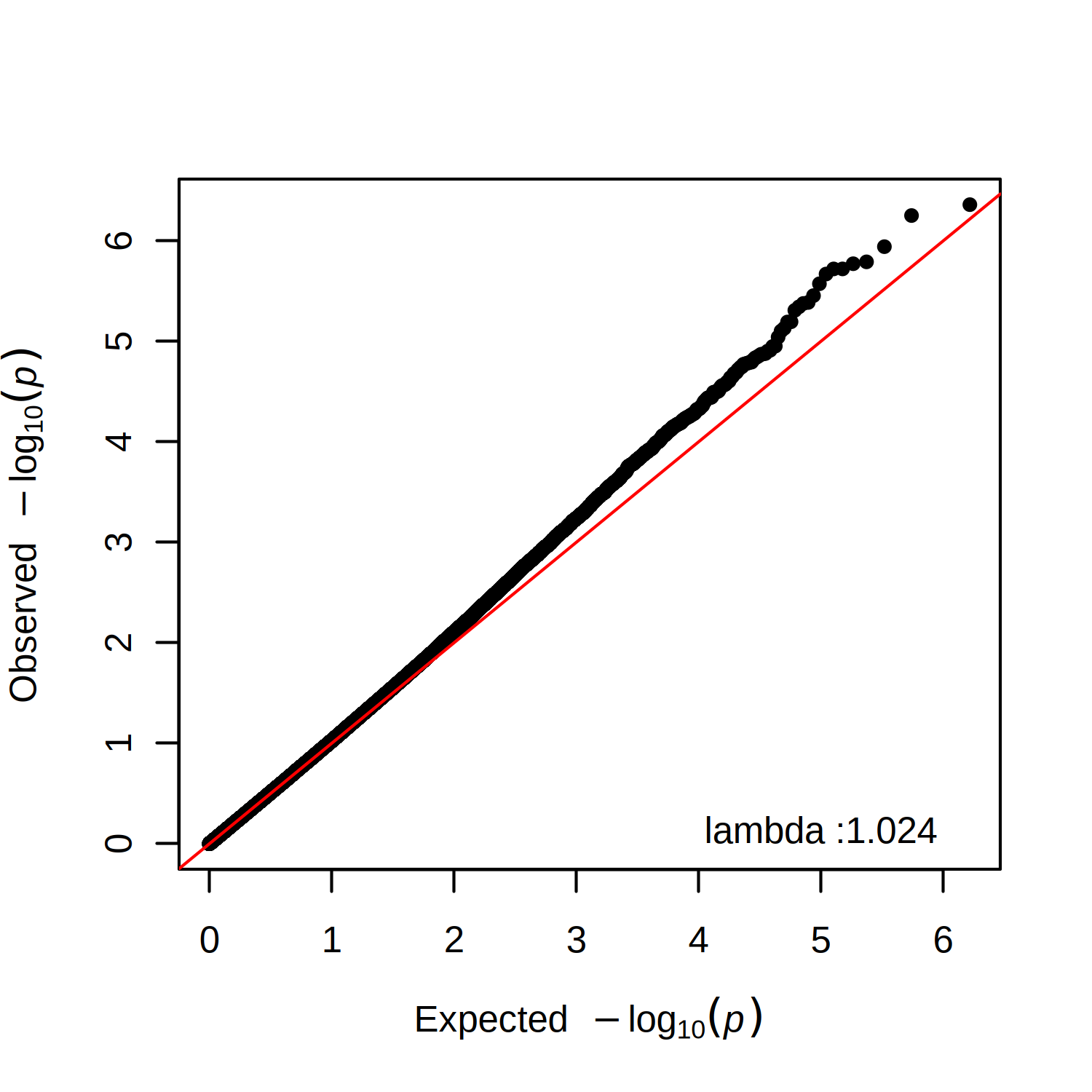 | 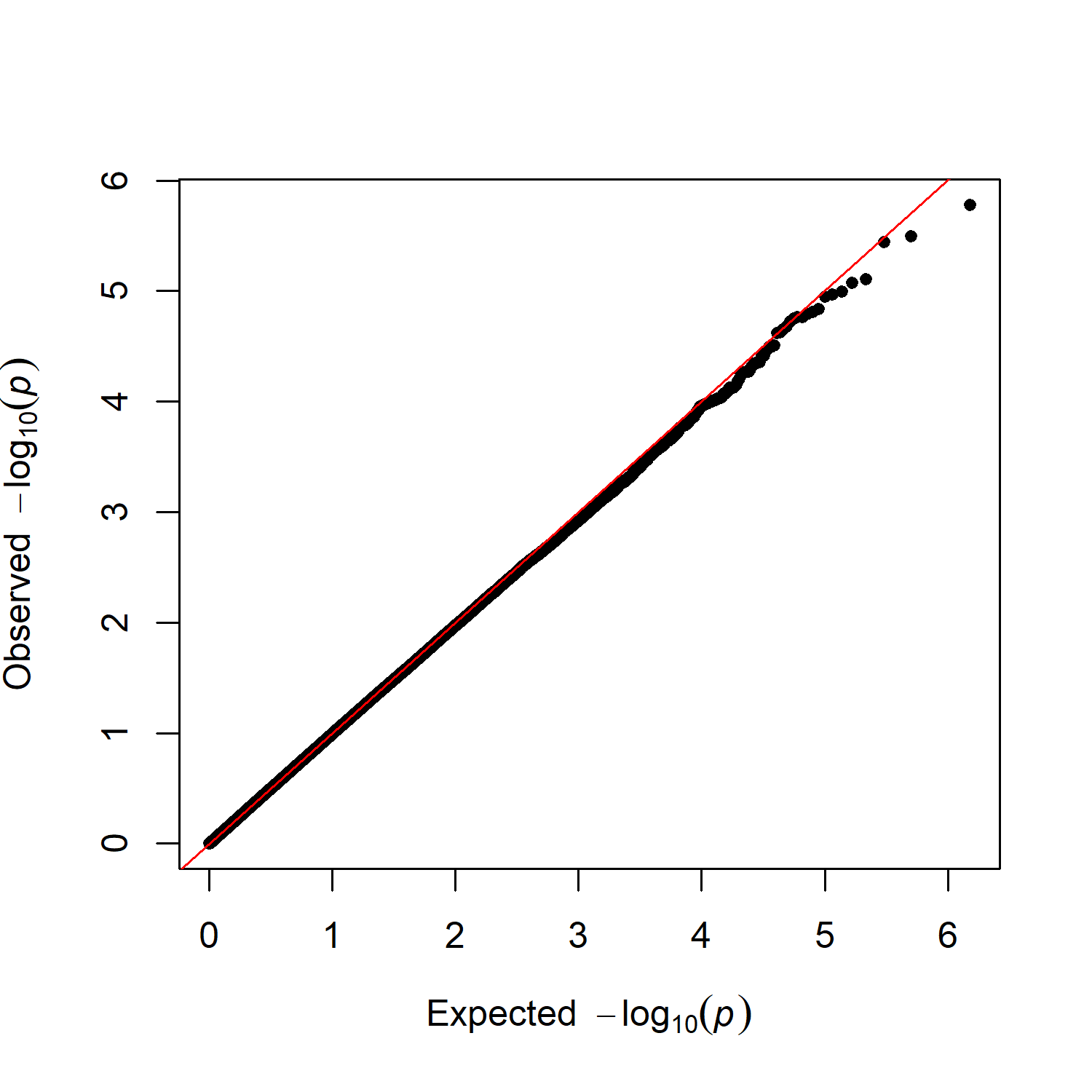 |
| 1. TOP3 | 1. TOP+ | 1. UTHealth-Houston |
|  | | |

## Figure S4: Forest plots and leave-one-out plots for DMPs.

For each significant DMP (defined by the CpG reference number) the left graph represents the effects in each cohort (box) with confidence interval (line), the meta-analysis is displayed as a diamond. SA- sex adjusted analysis, M- Male sample, F- Female sample. The graph on the right displays the changes in effect when the respective cohorts are left out of the meta-analysis. A. CpG significant in the total sample EWAS. B. CpG significant in the Female only EWAS.

|  | | | |
| --- | --- | --- | --- |
| 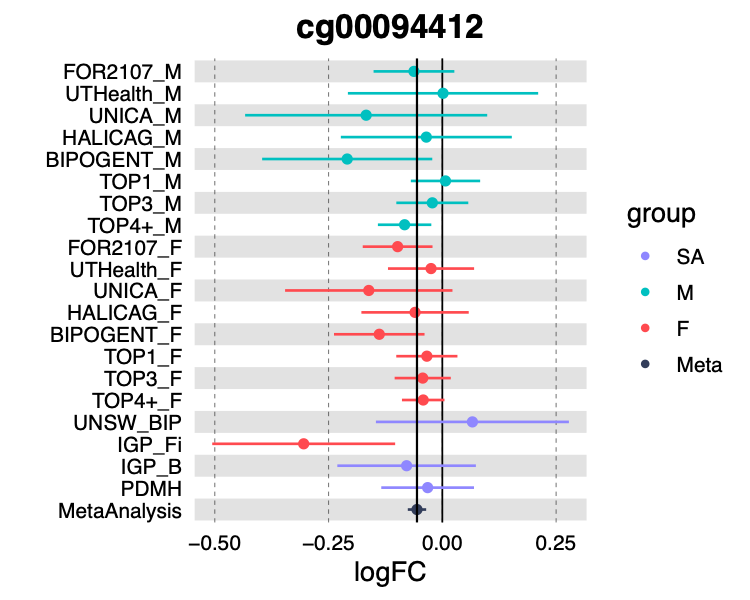 | 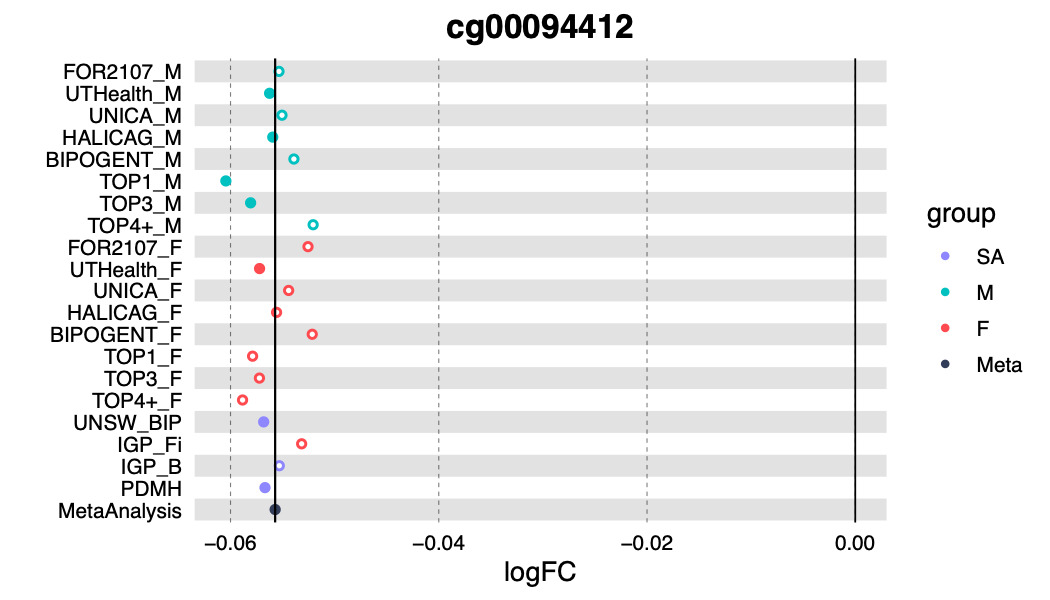 | 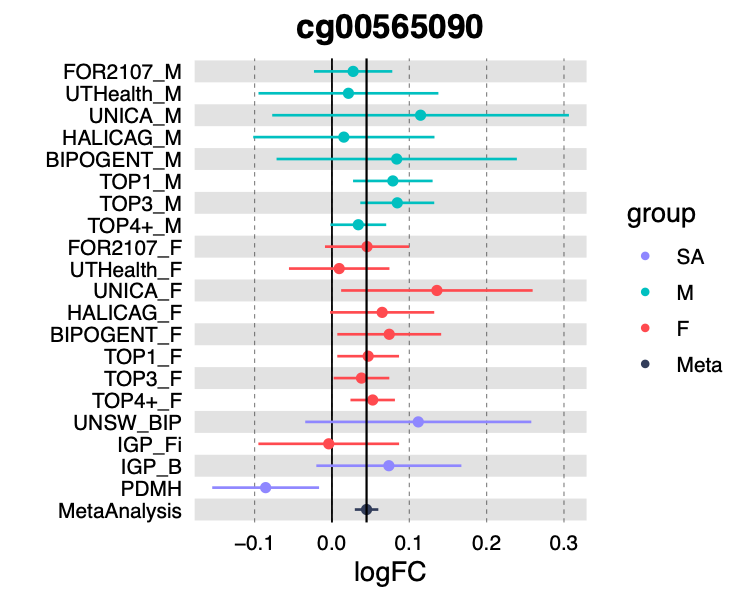 | 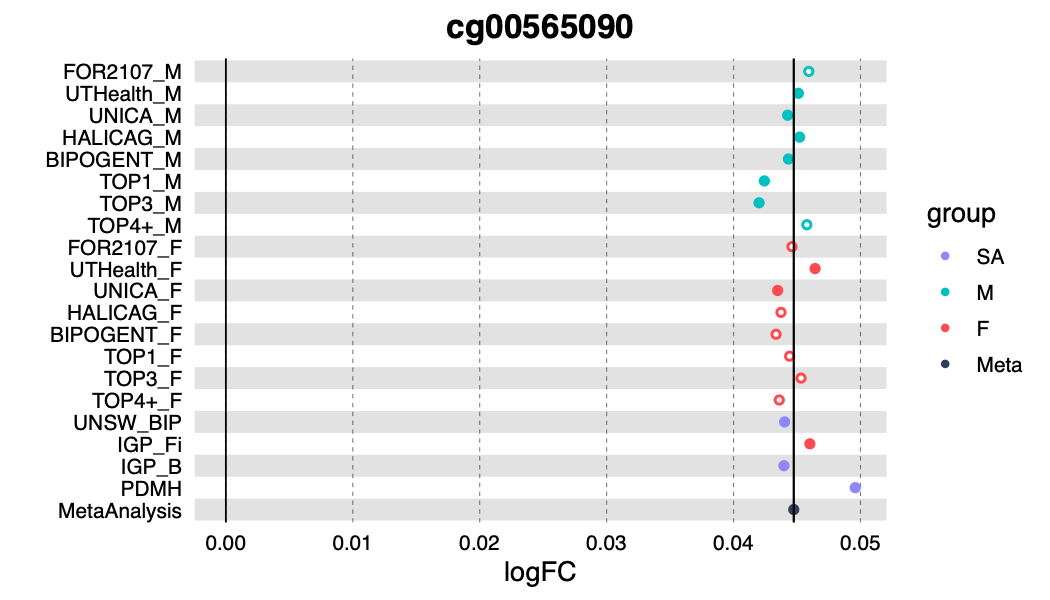 |
| 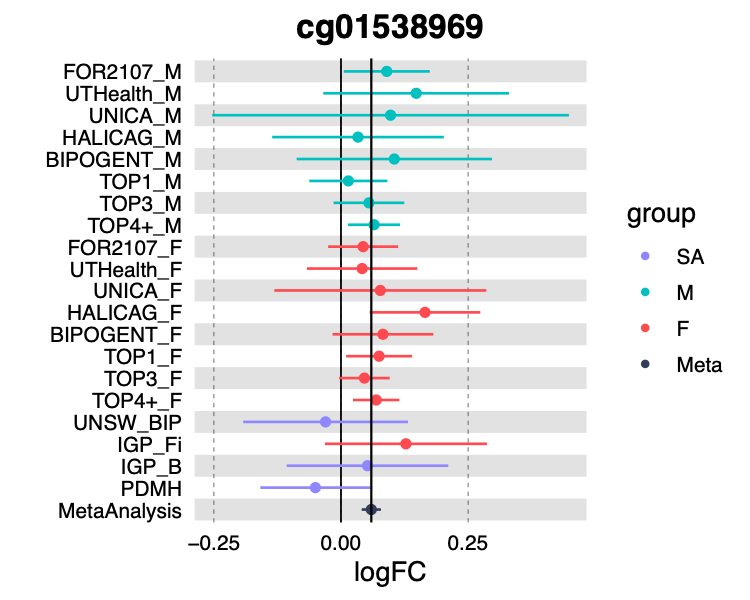 | 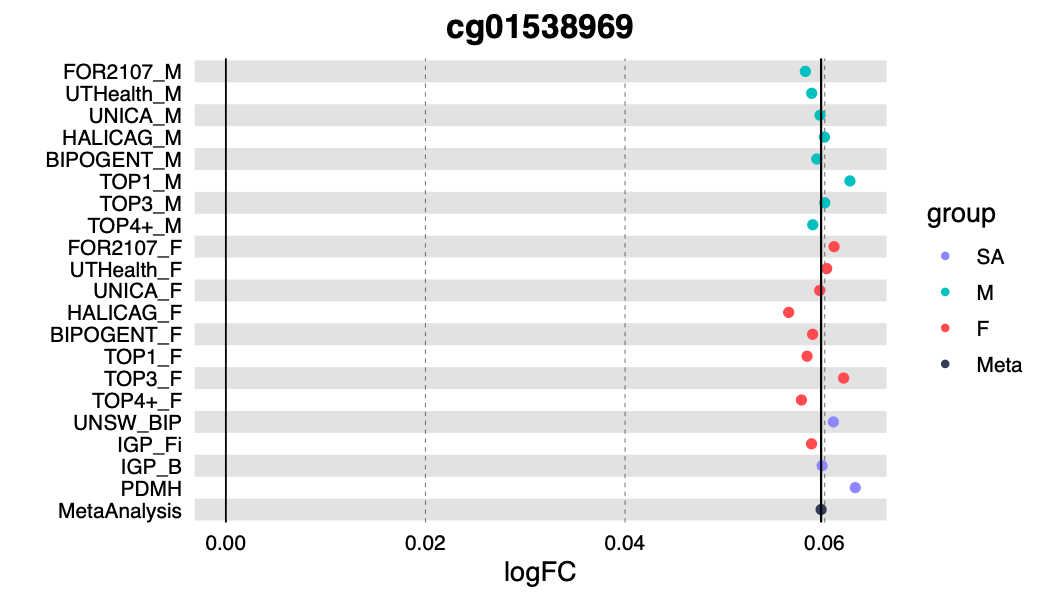 | 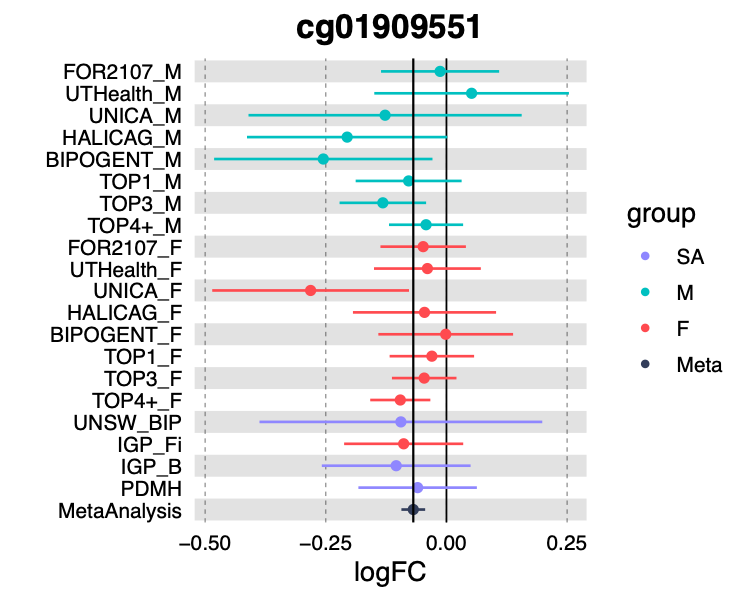 | 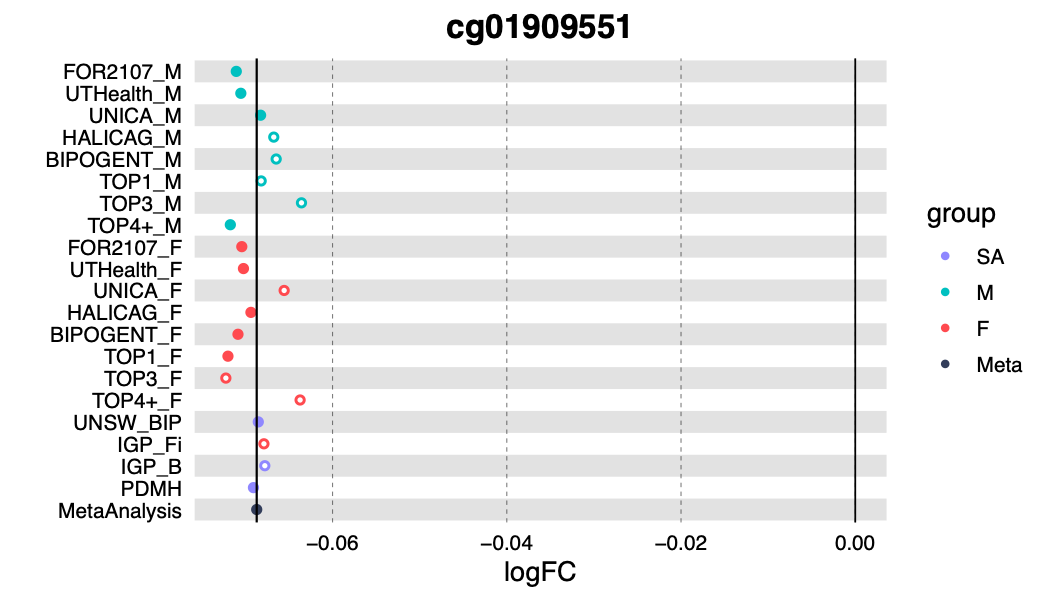 |
| 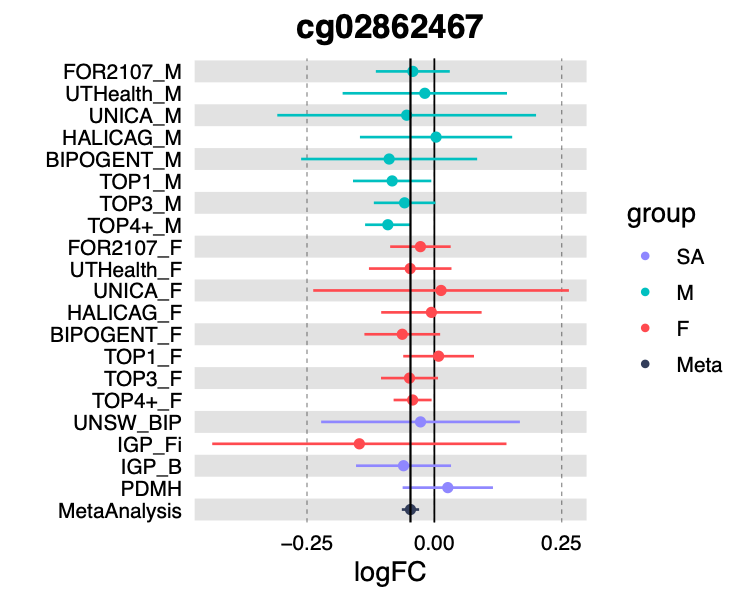 | 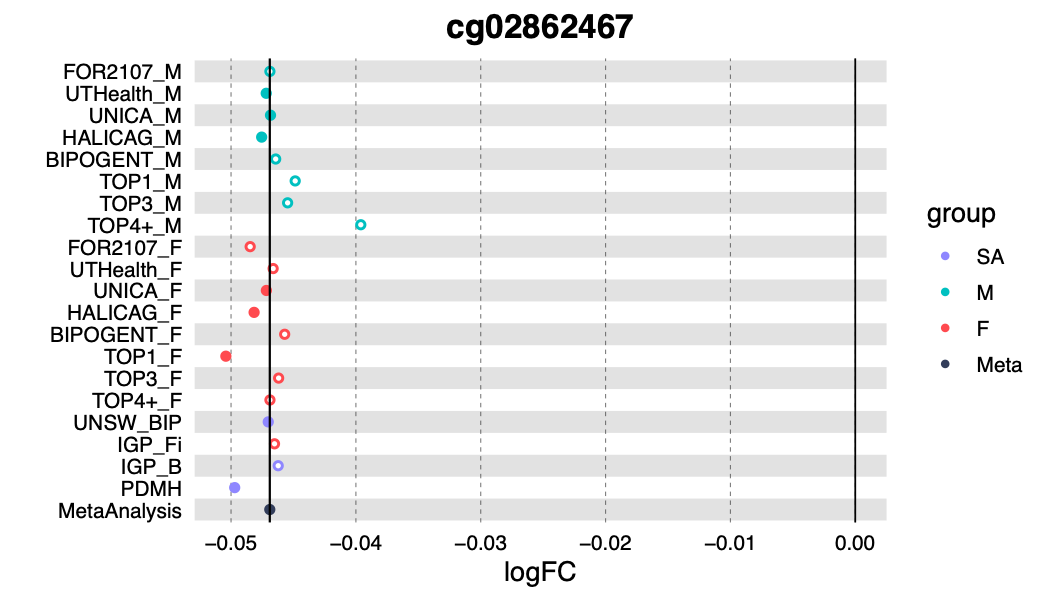 | 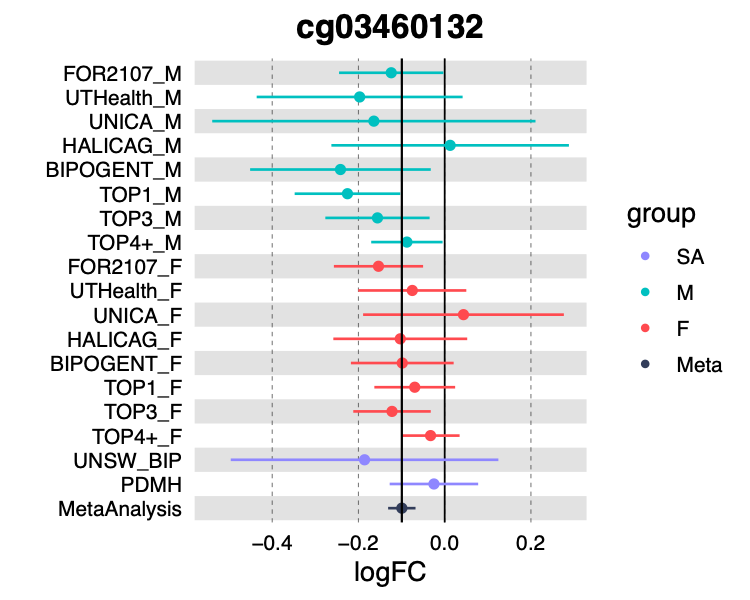 | 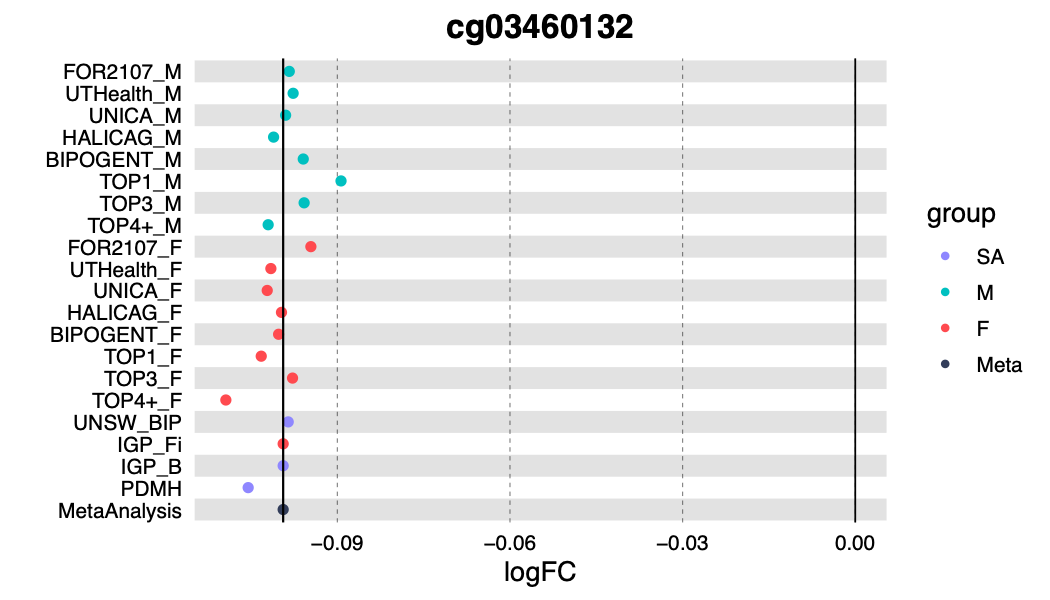 |
| 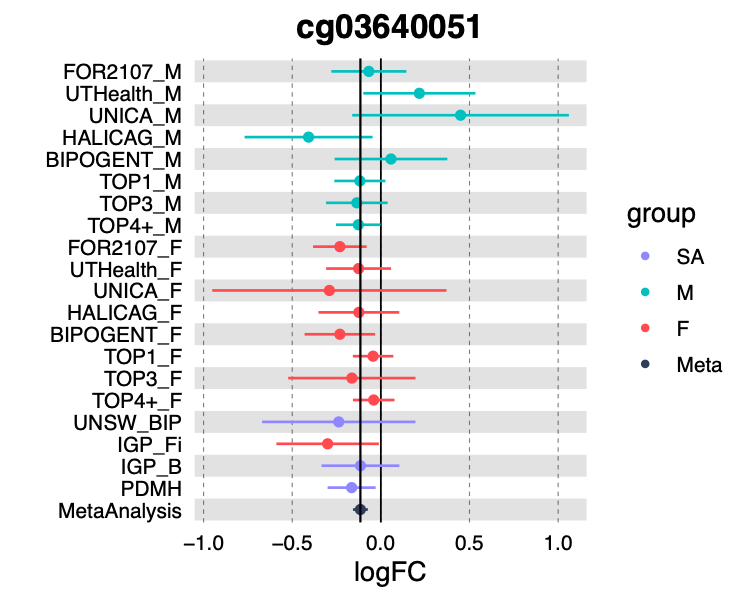 | 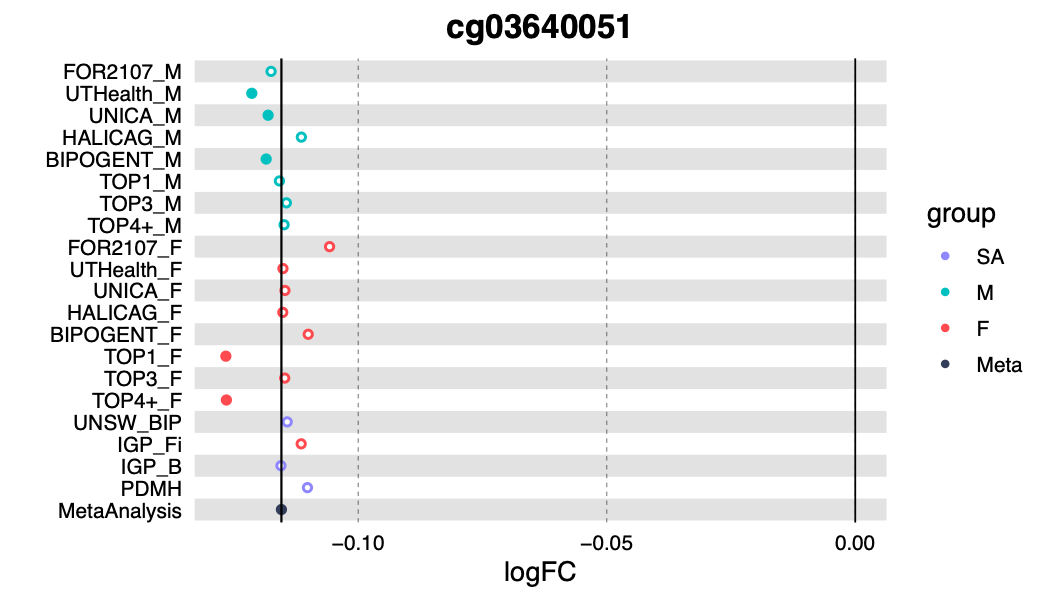 | 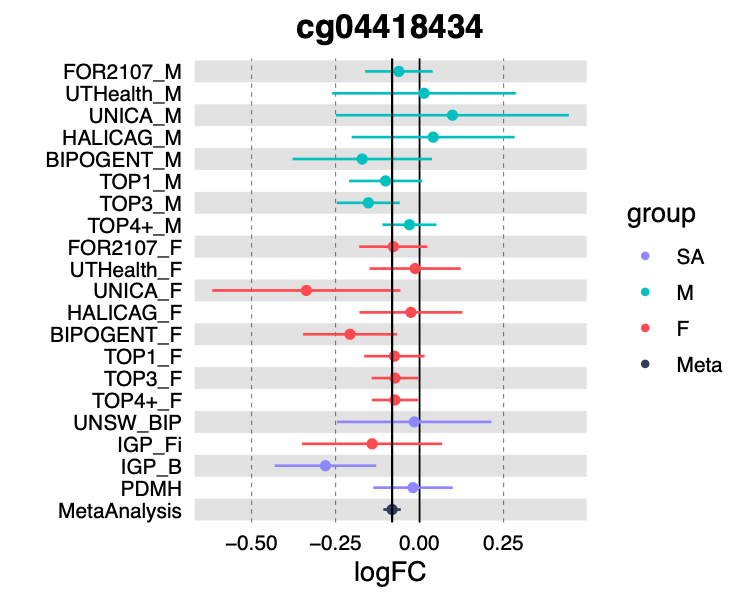 | 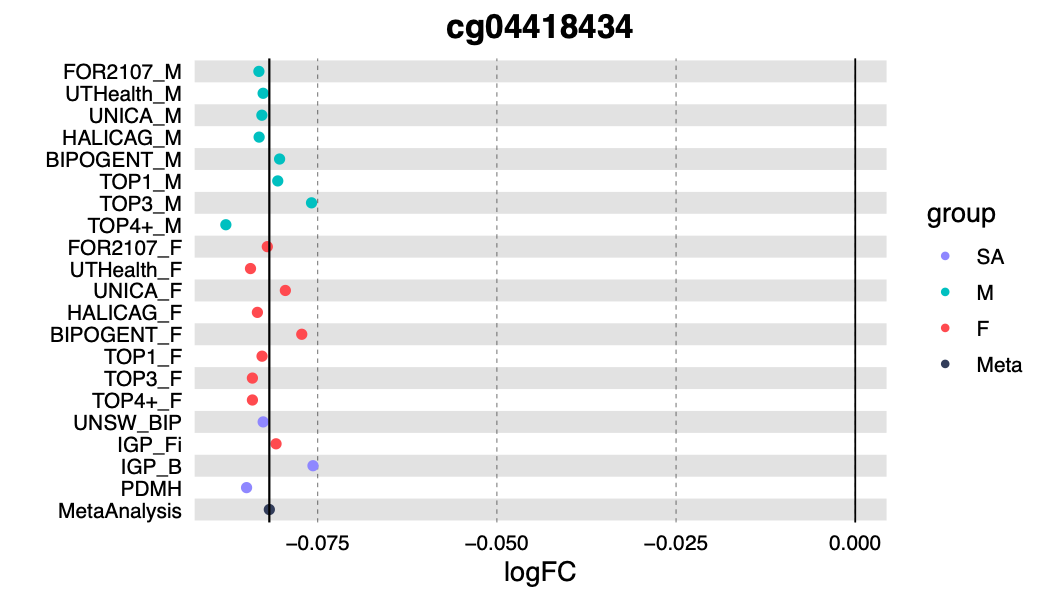 |
| 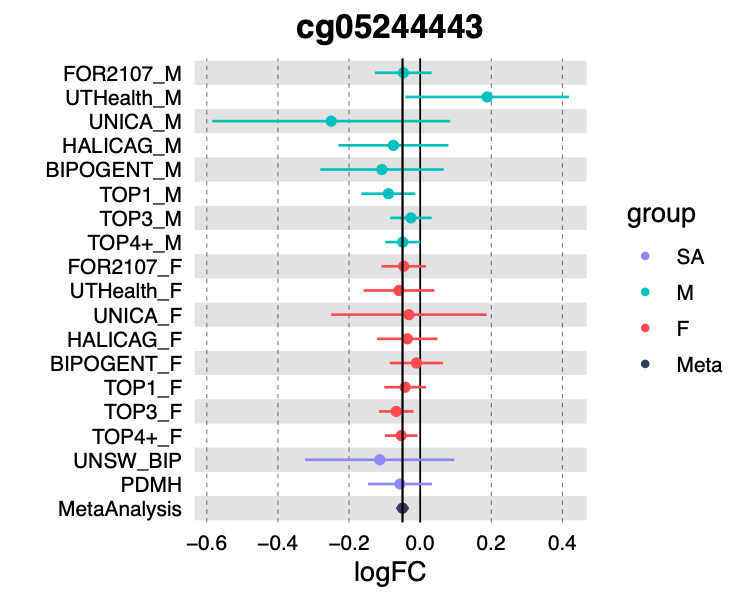 | 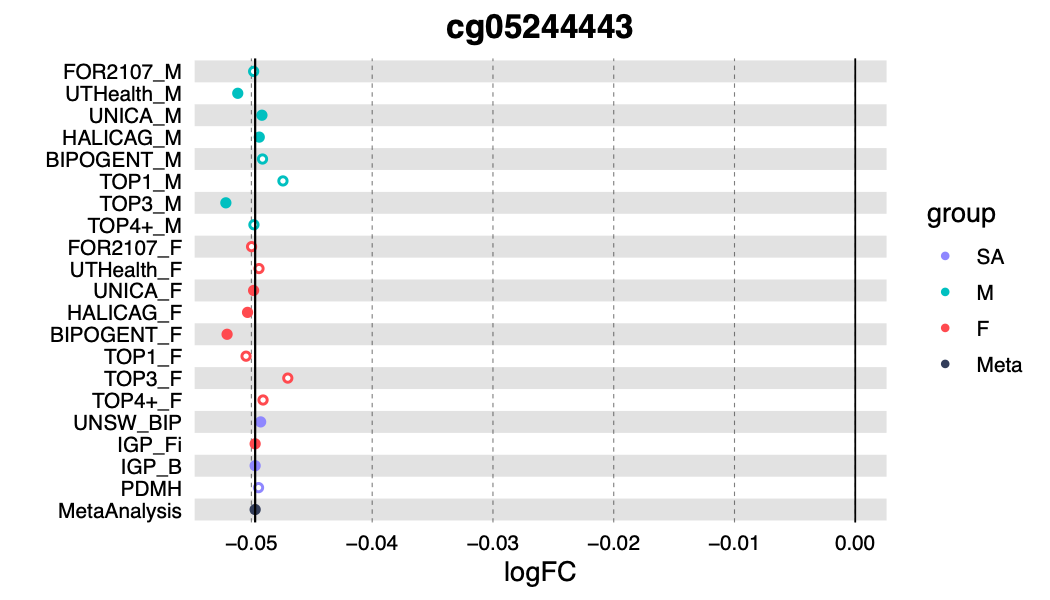 | 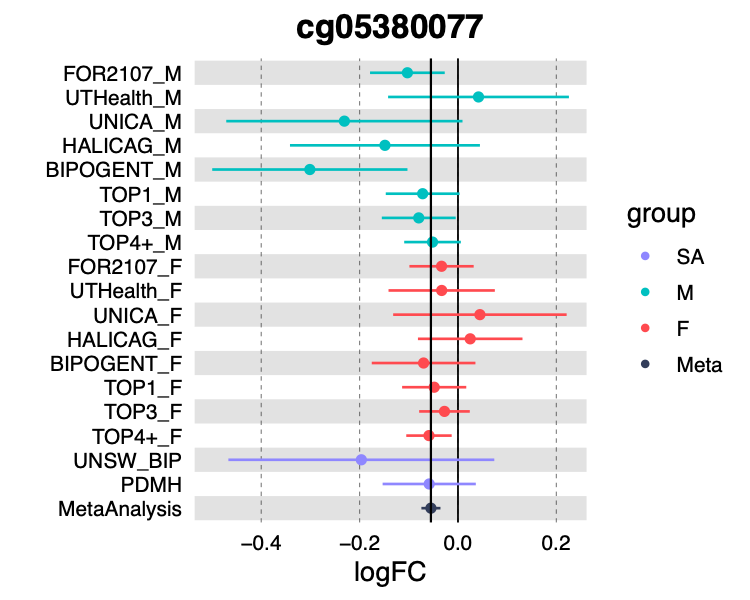 | 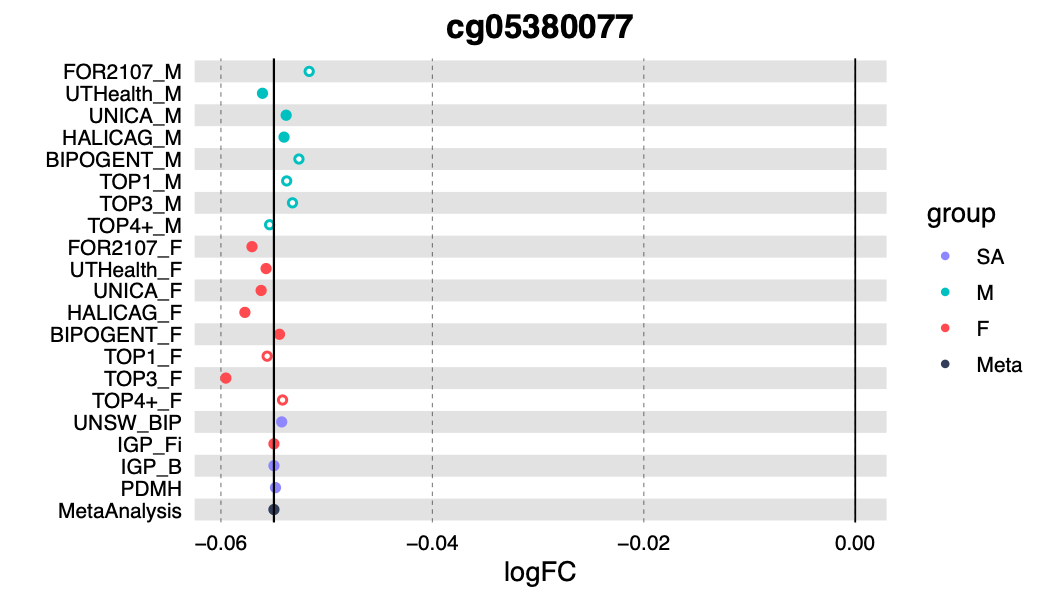 |
| 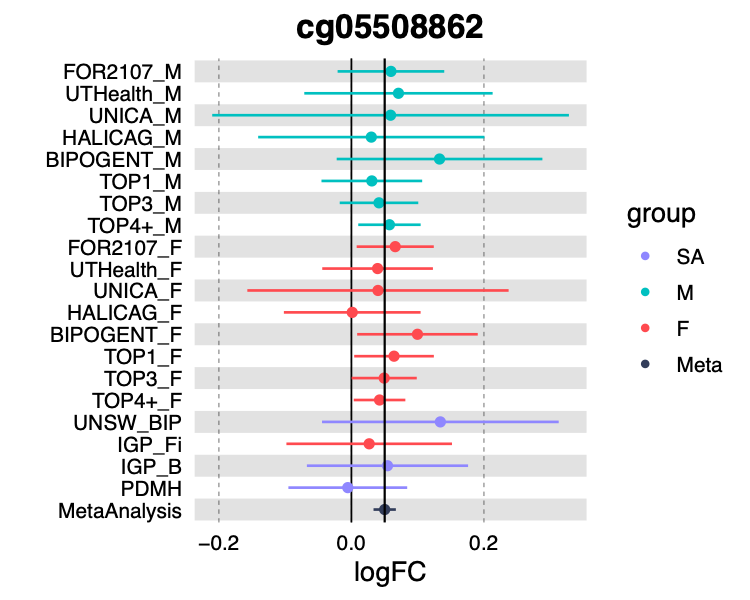 | 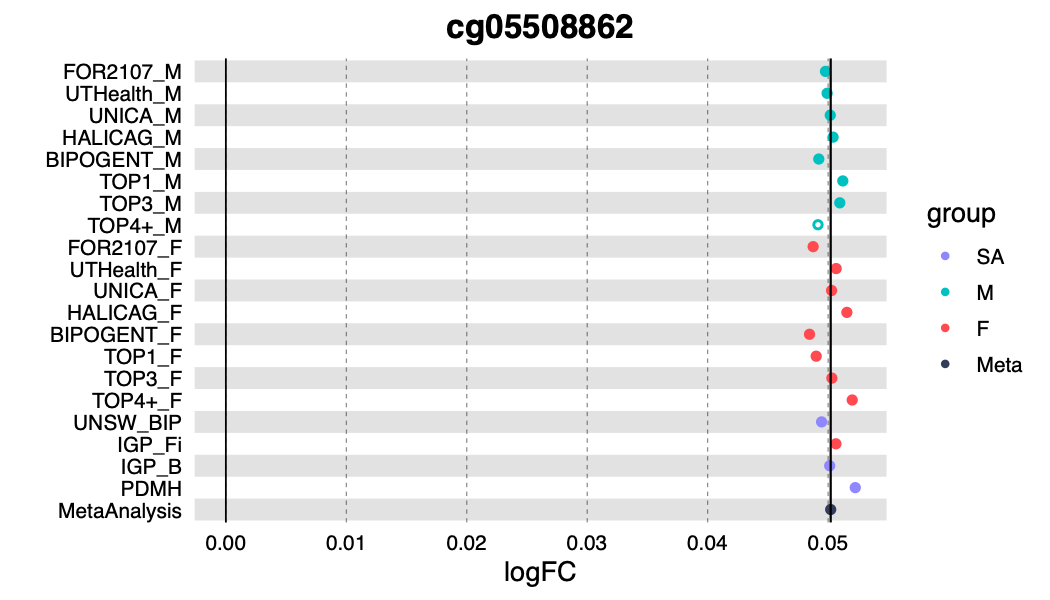 | 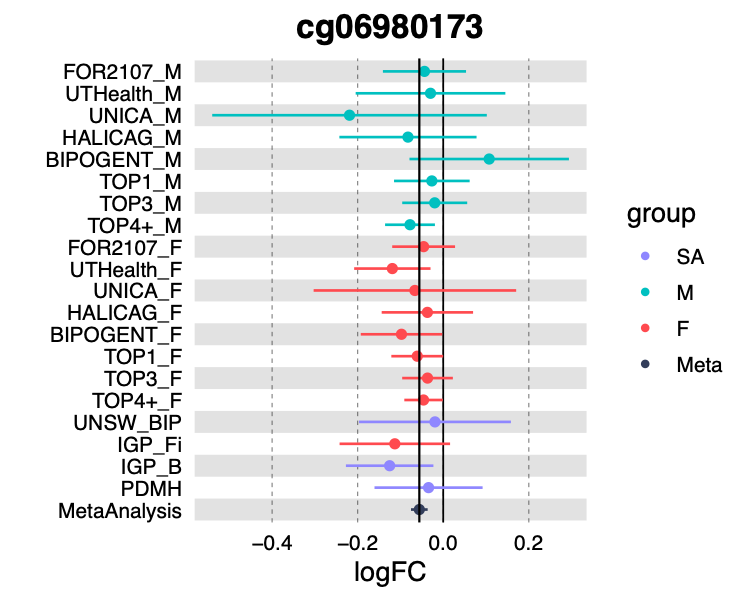 | 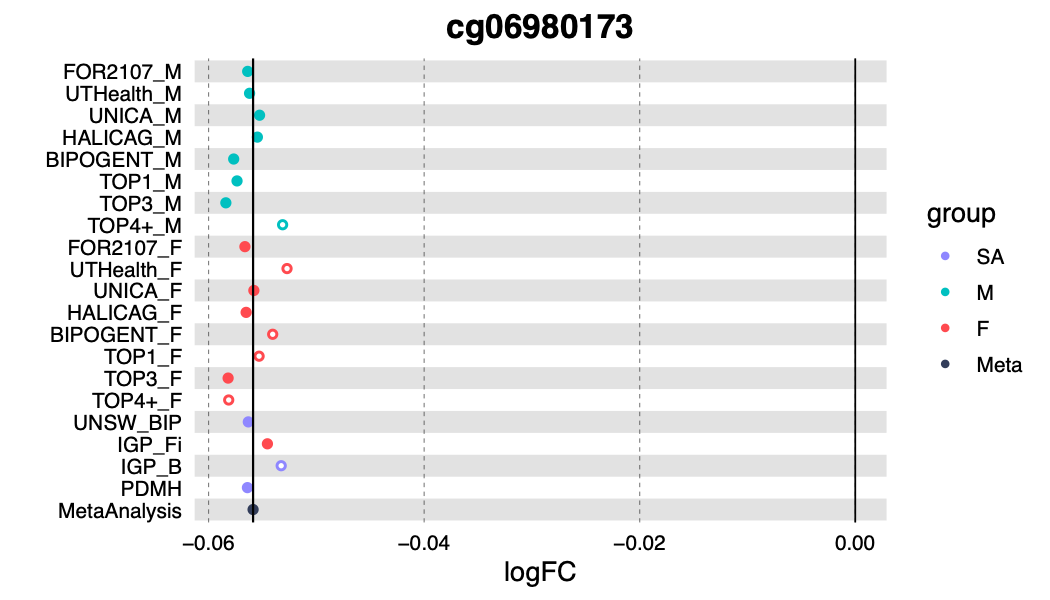 |
| 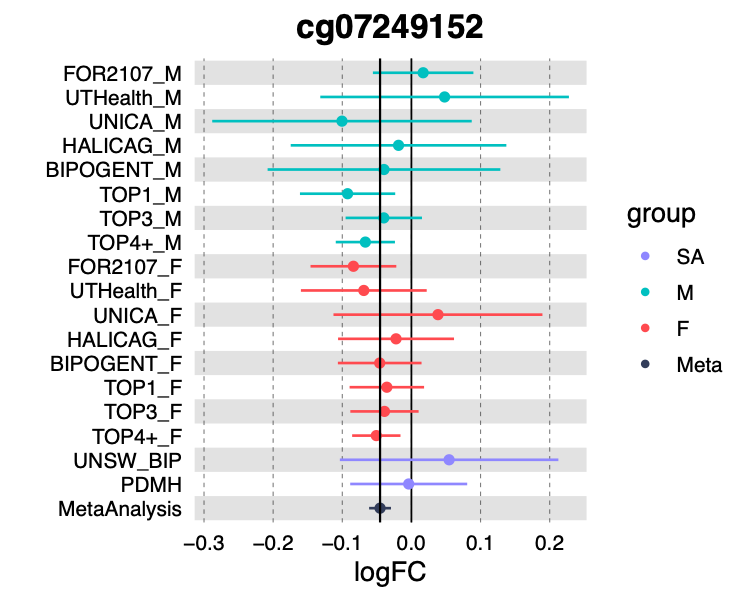 | 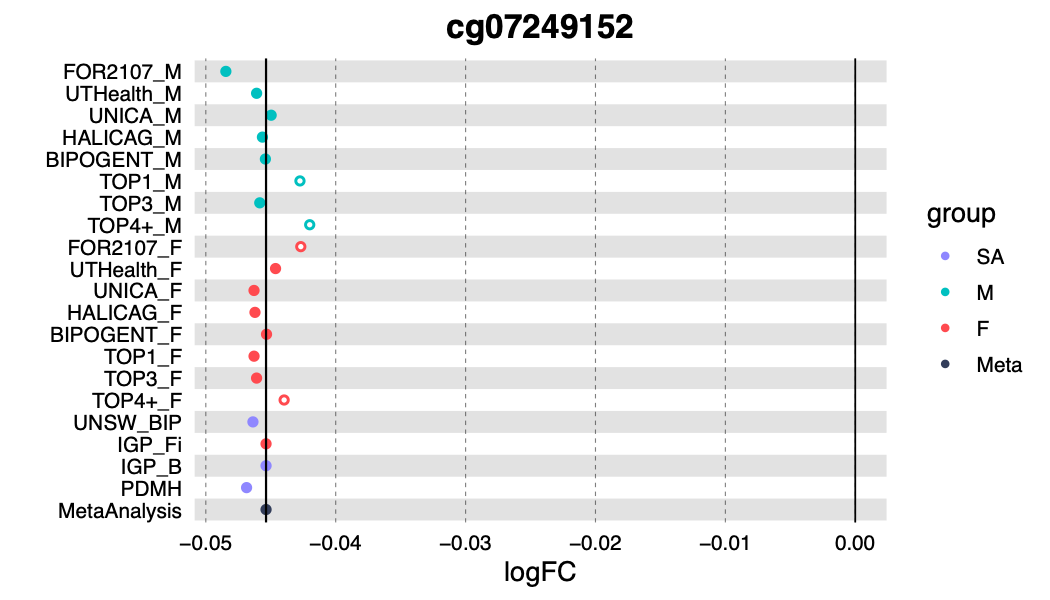 | 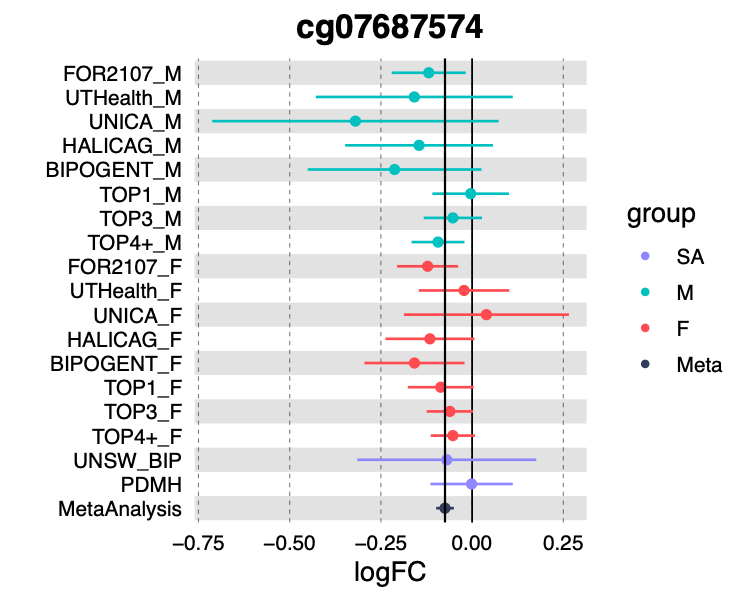 | 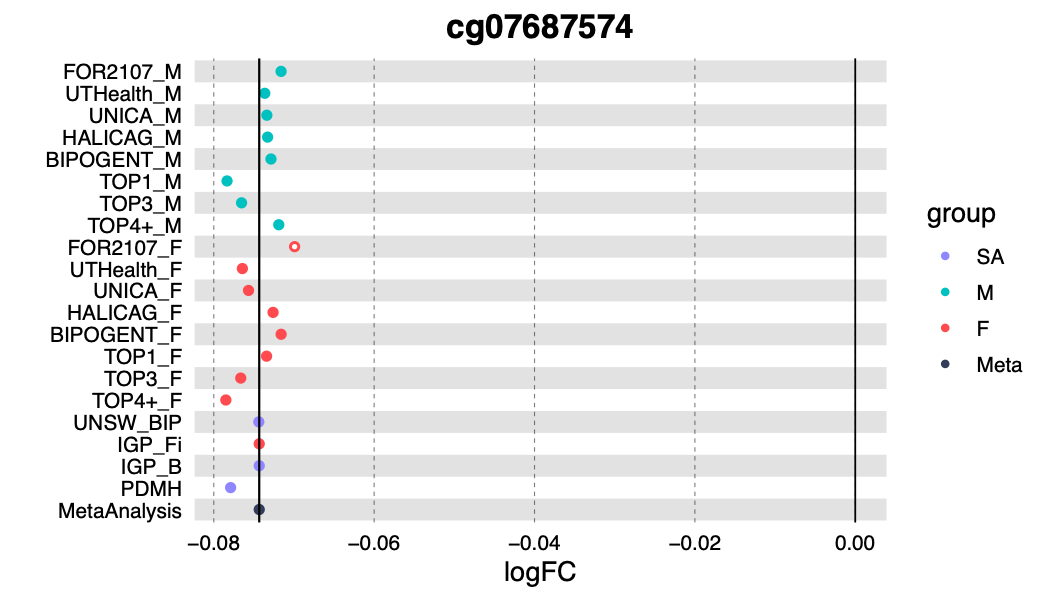 |
| 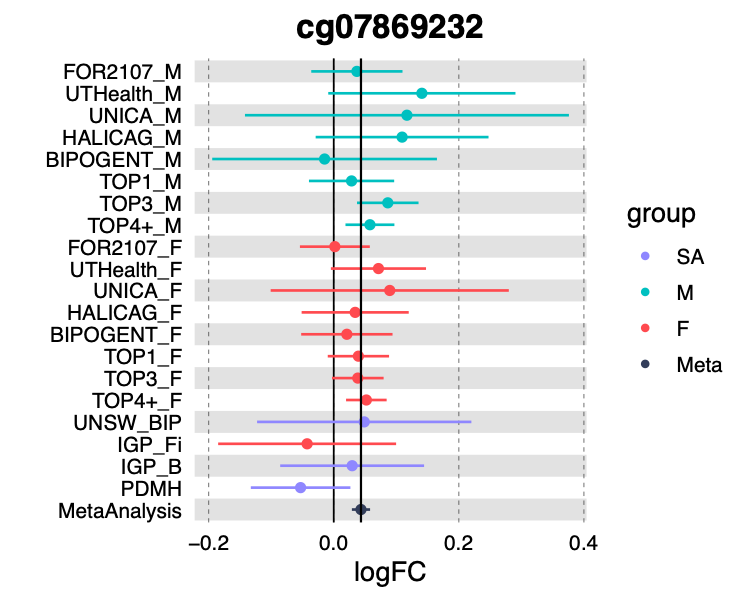 | 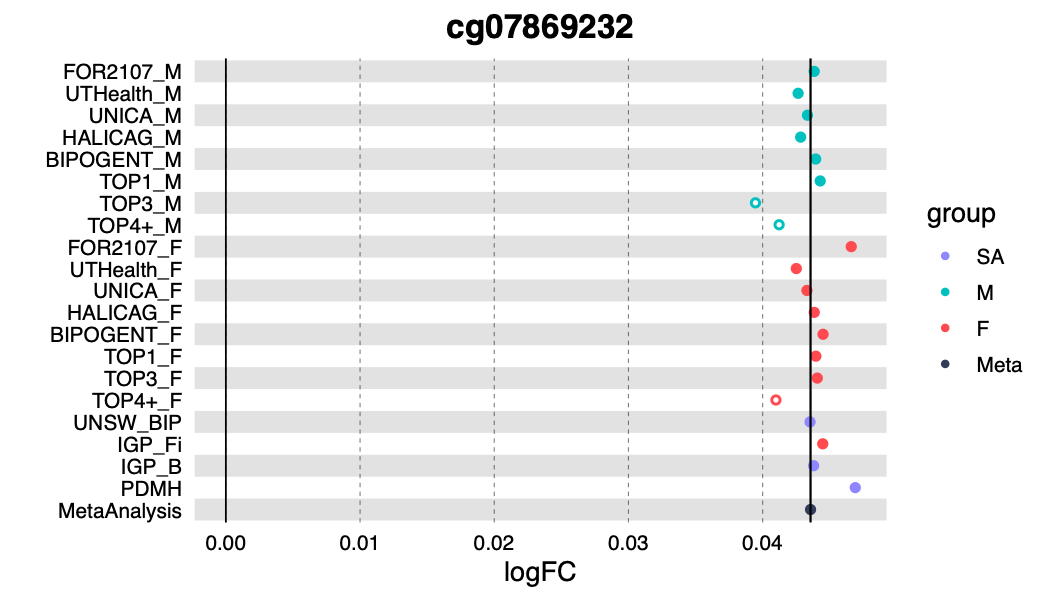 | 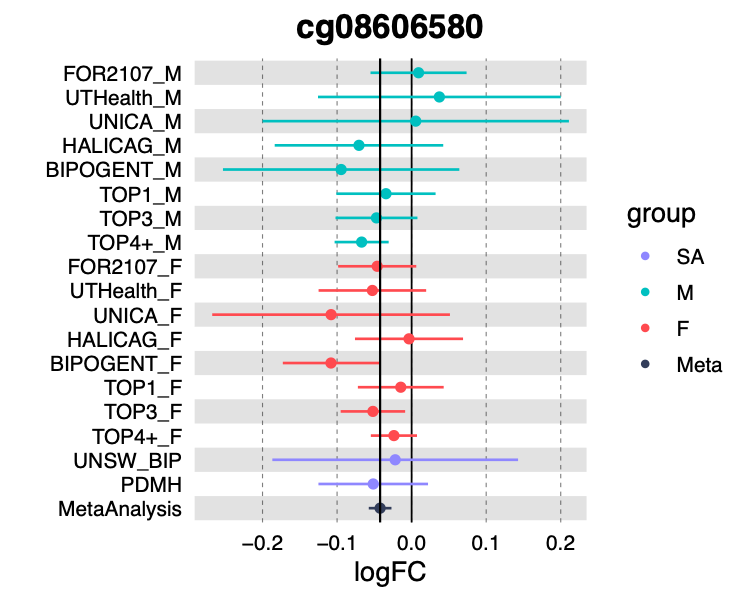 | 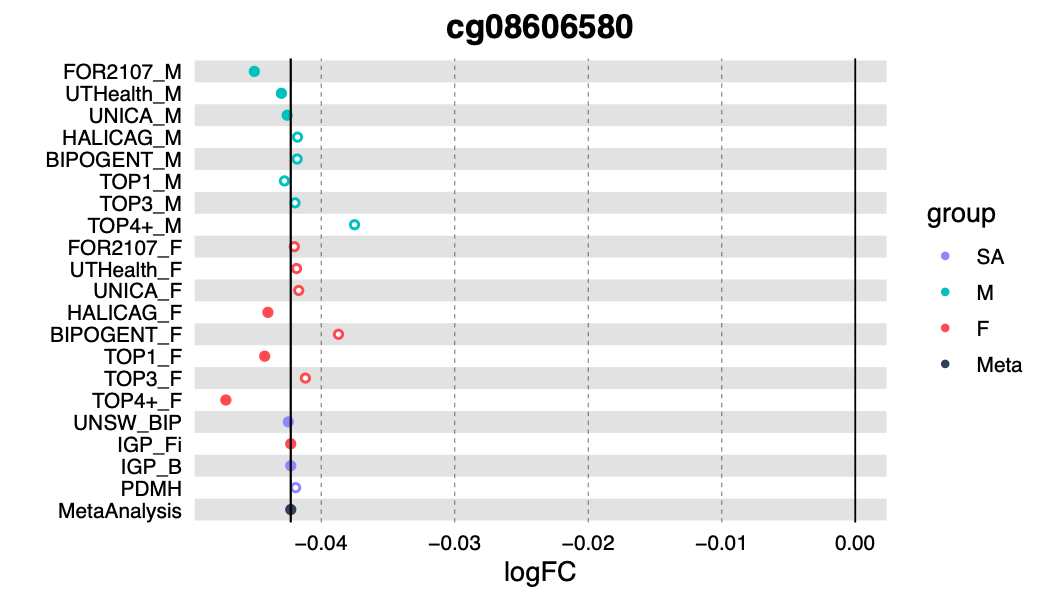 |
| 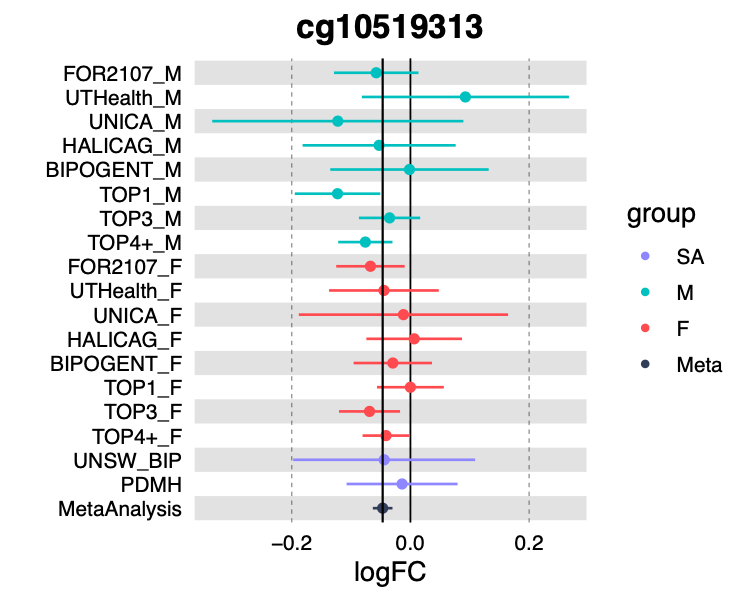 | 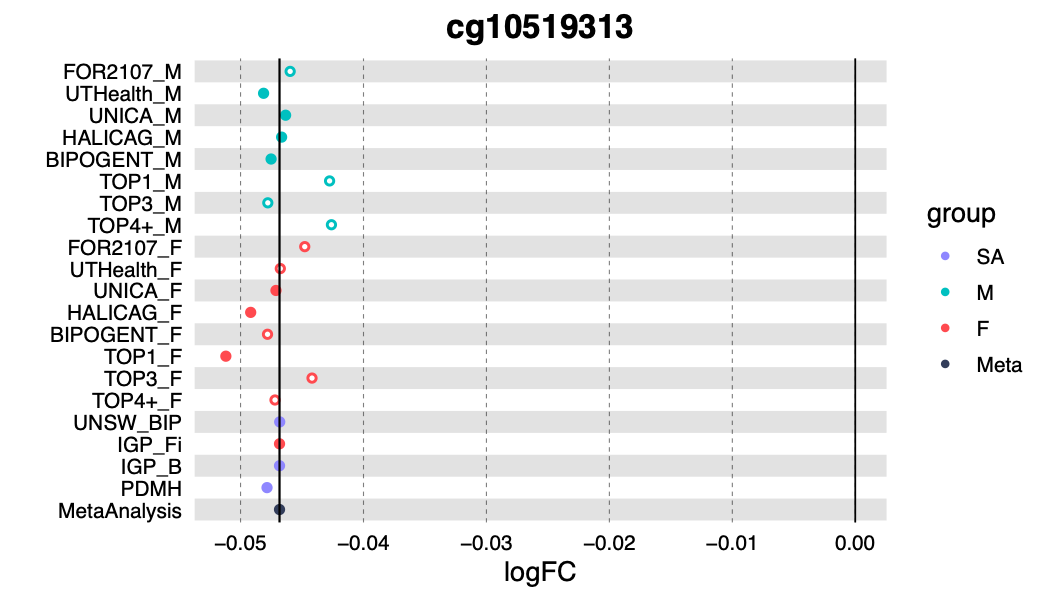 | 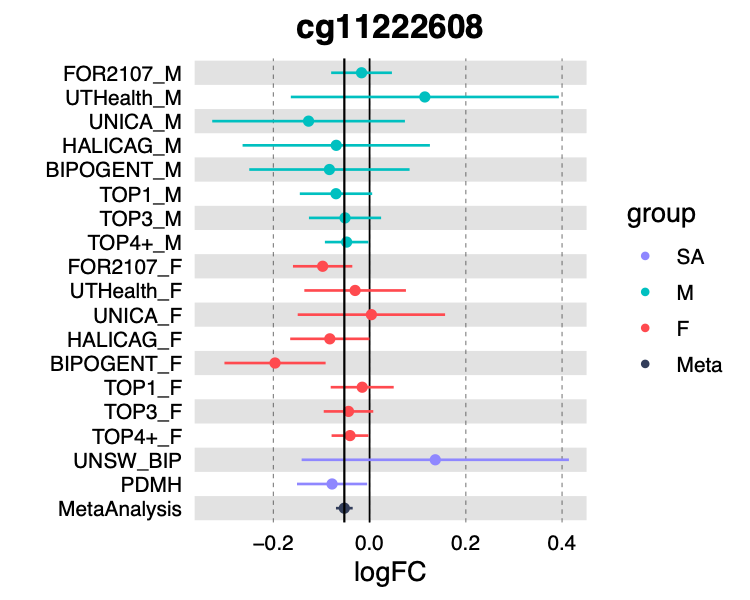 | 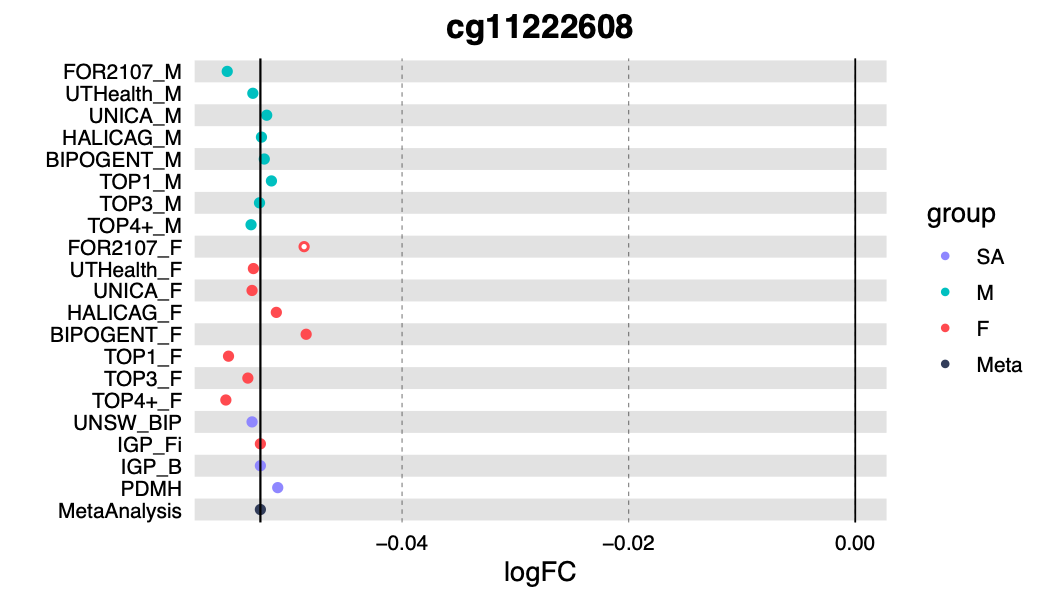 |
| 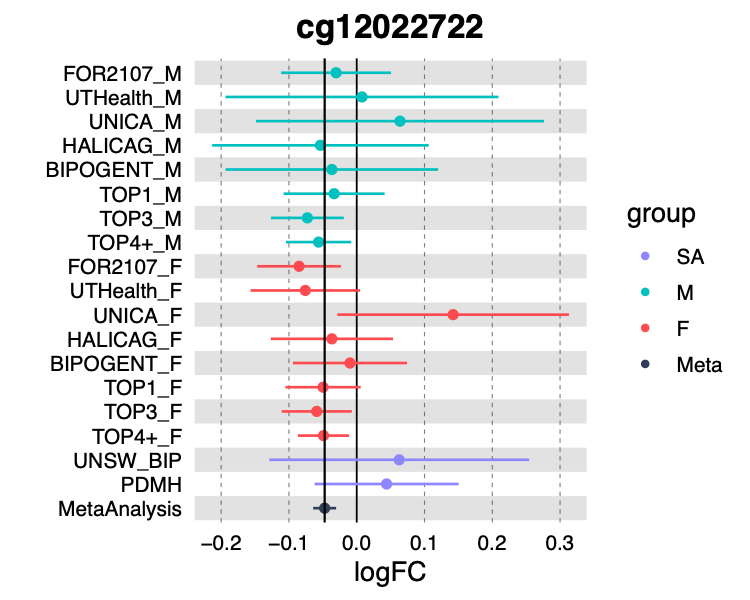 | 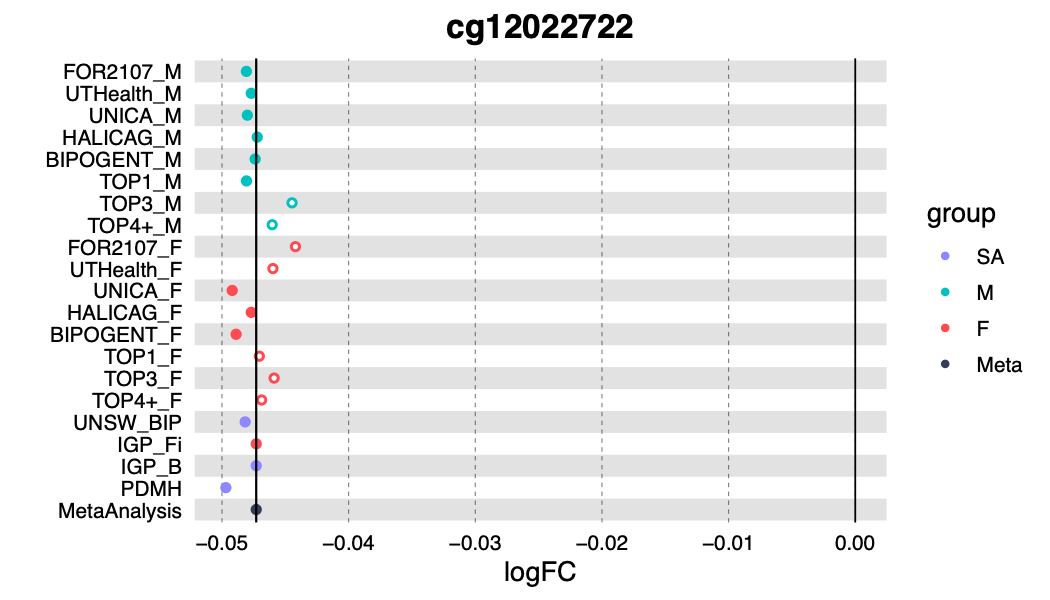 | 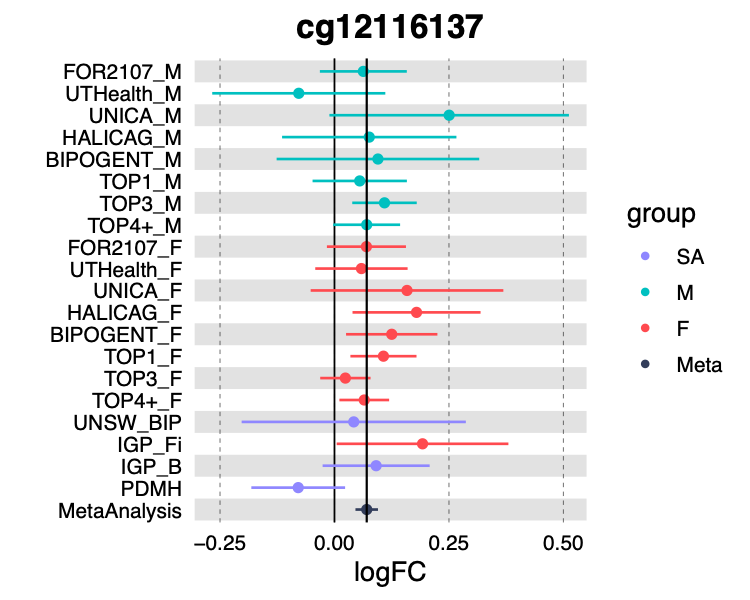 | 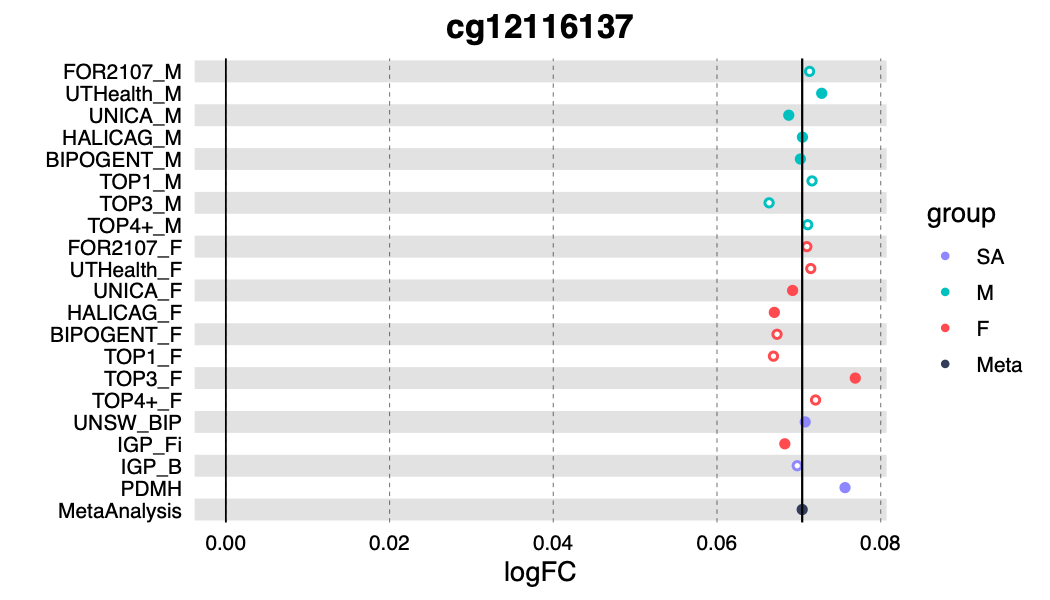 |
| 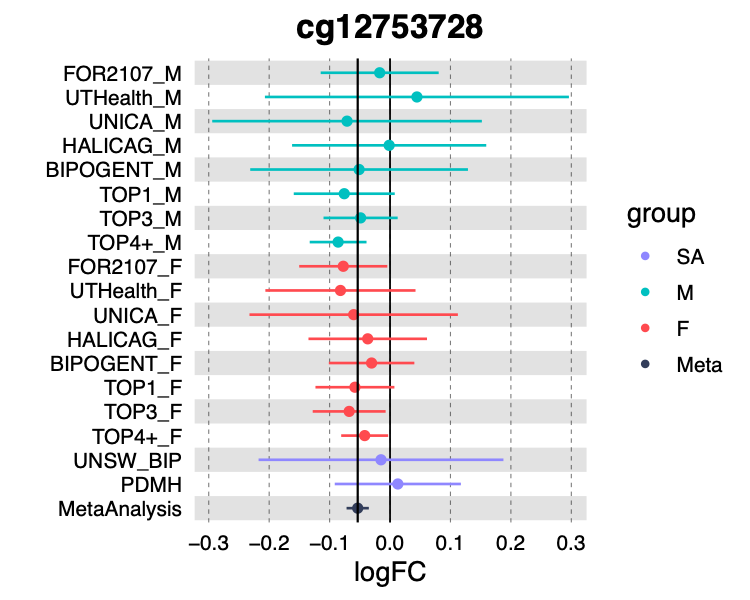 | 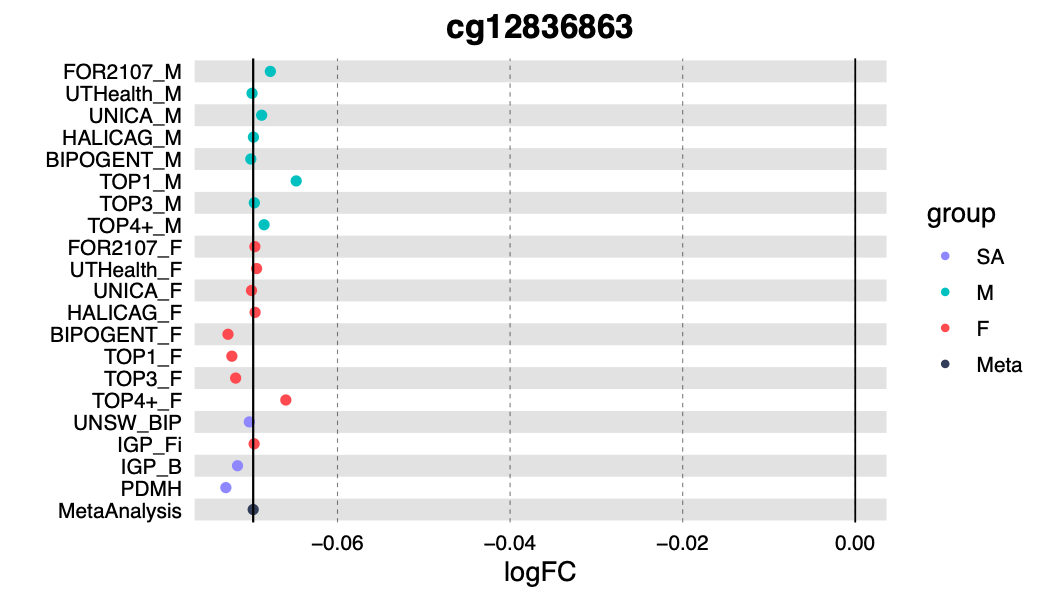 | 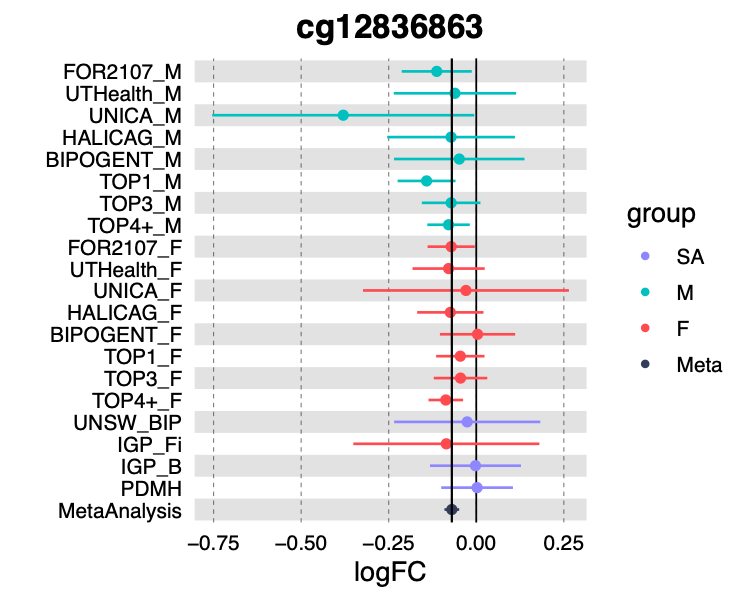 | 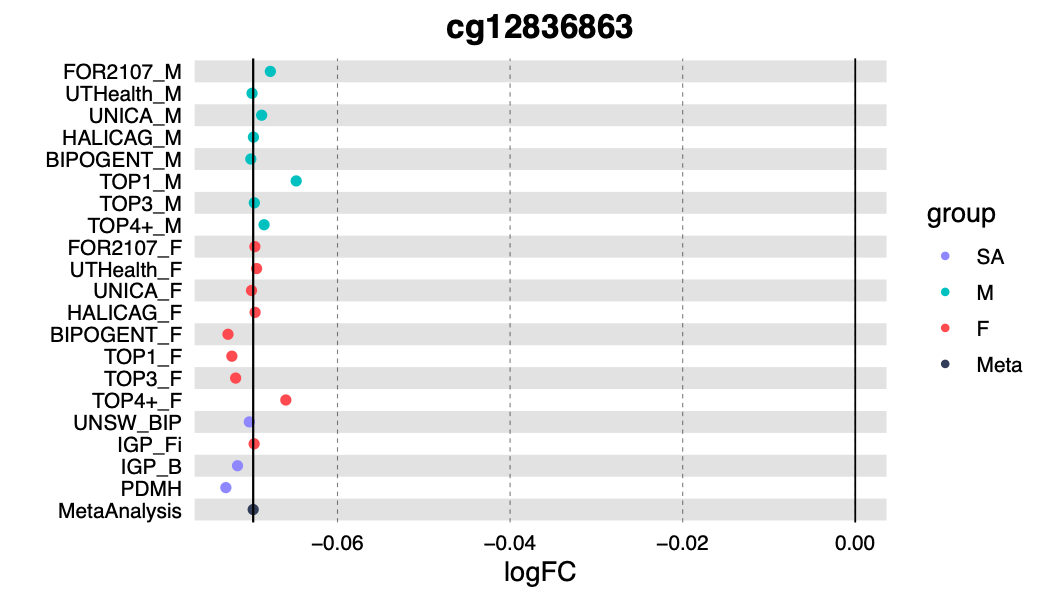 |
| 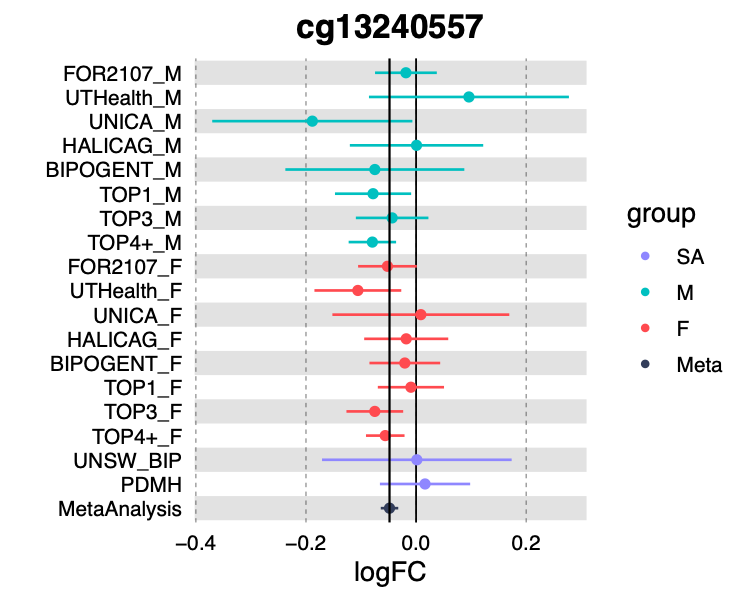 | 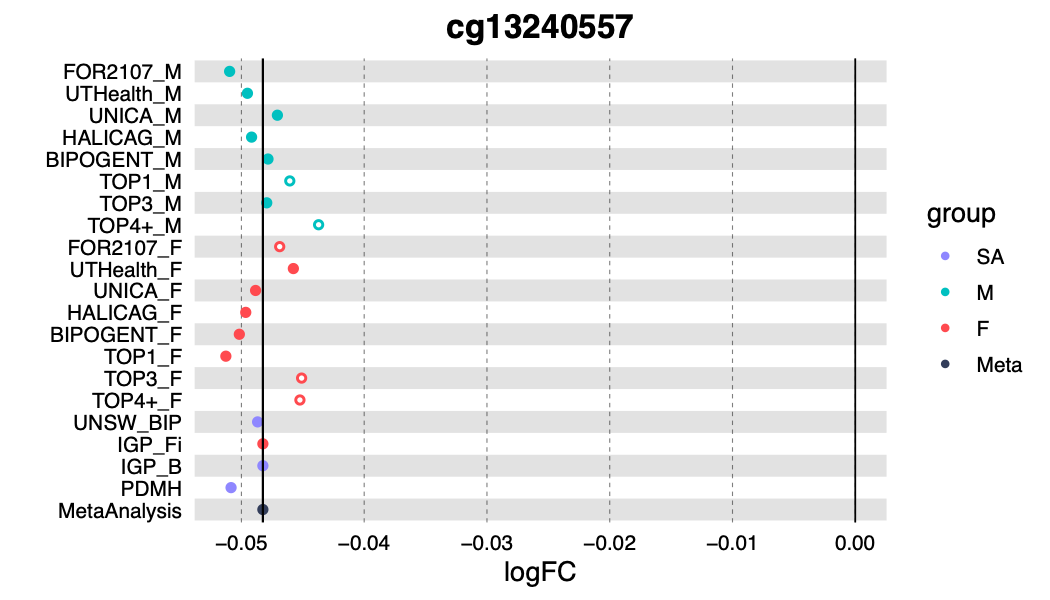 | 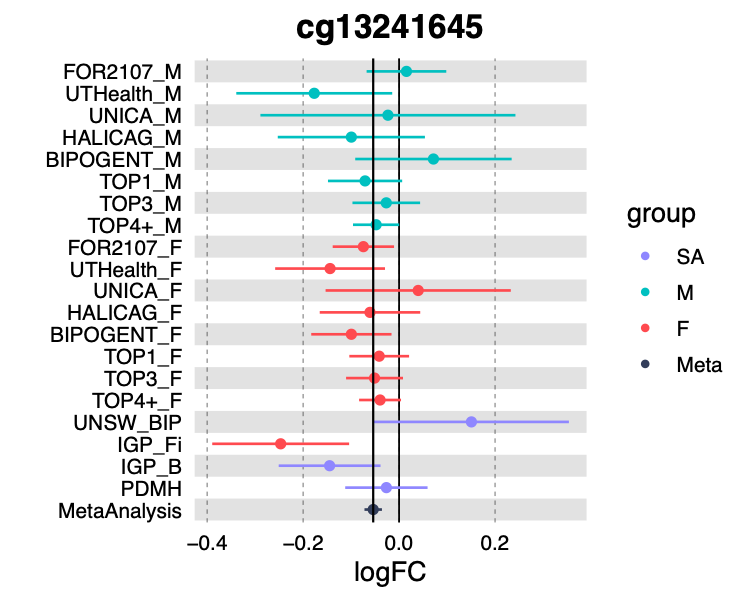 | 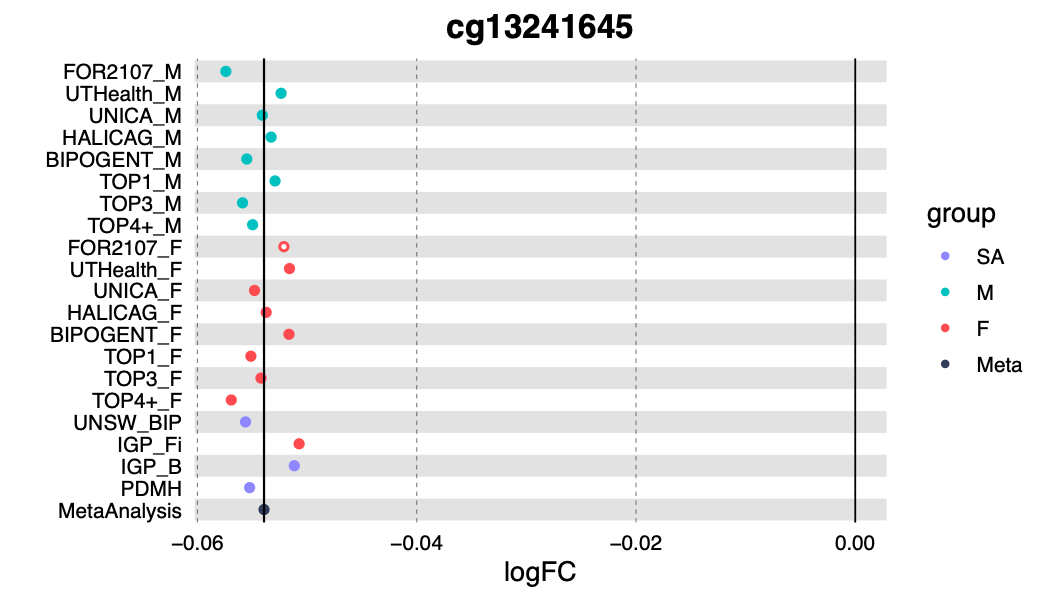 |
| 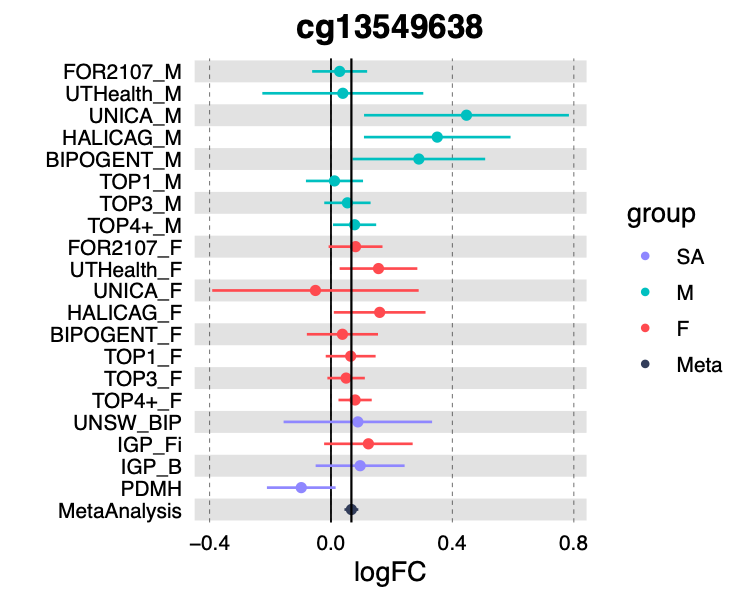 | 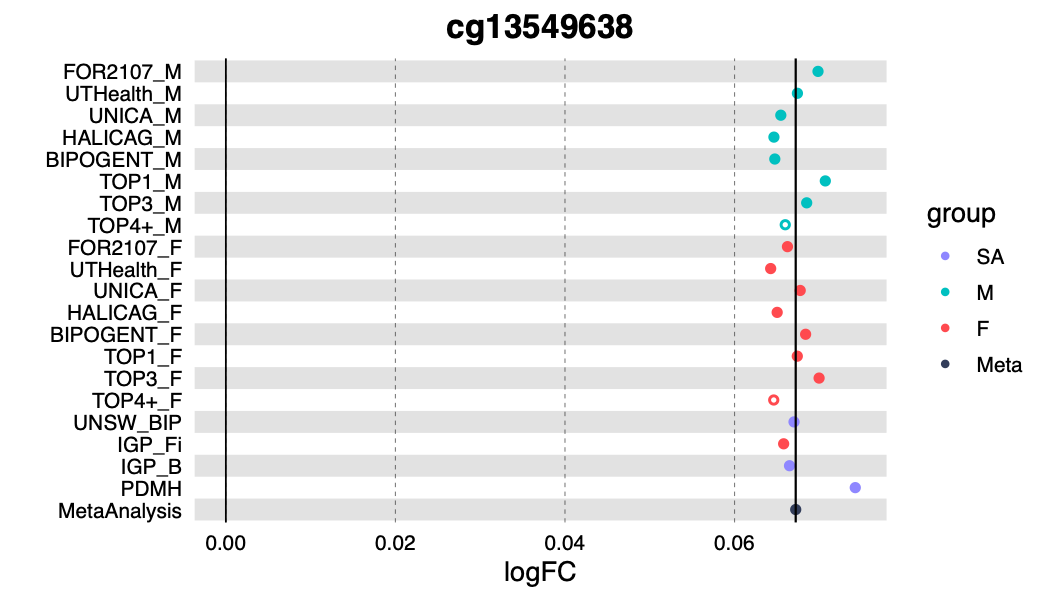 | 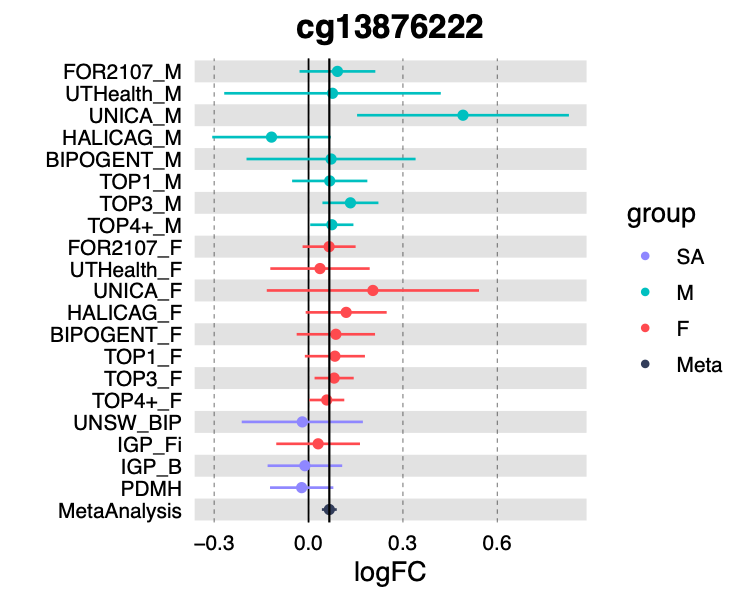 | 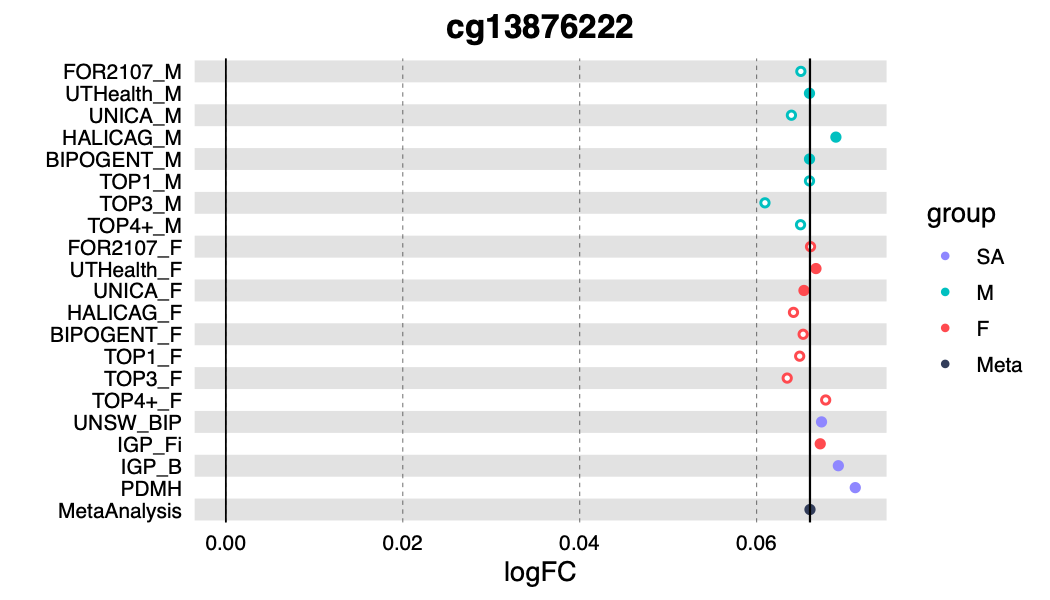 |
| 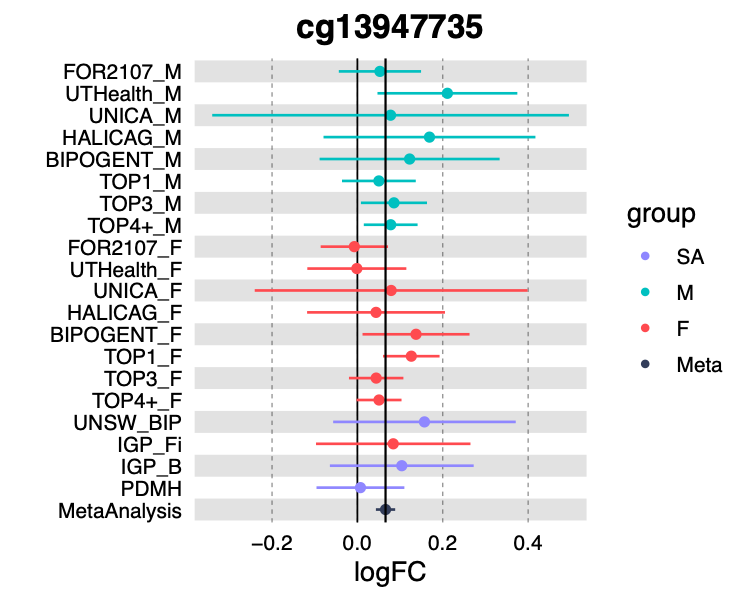 | 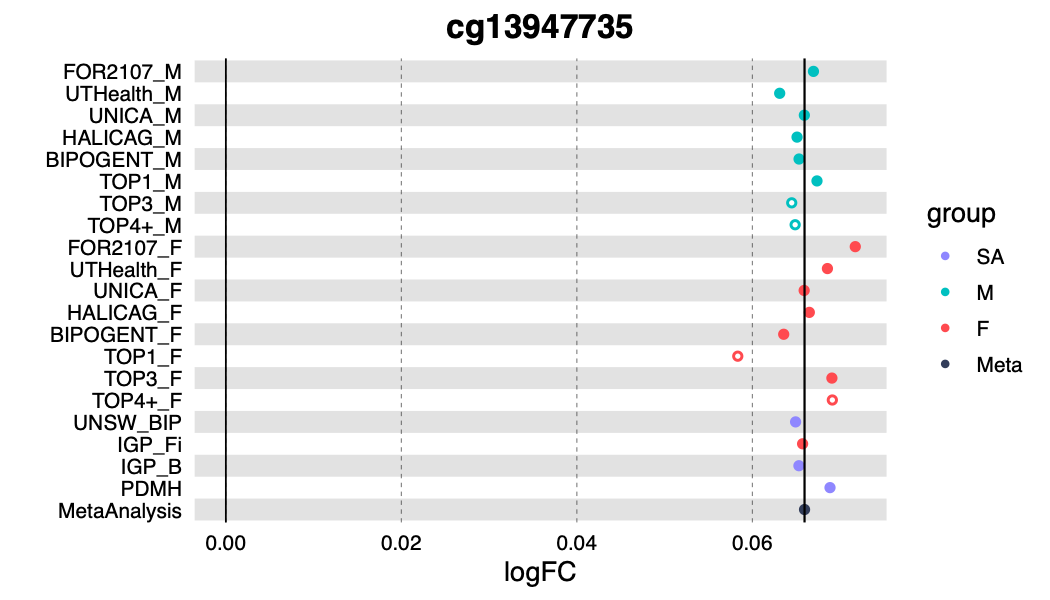 | 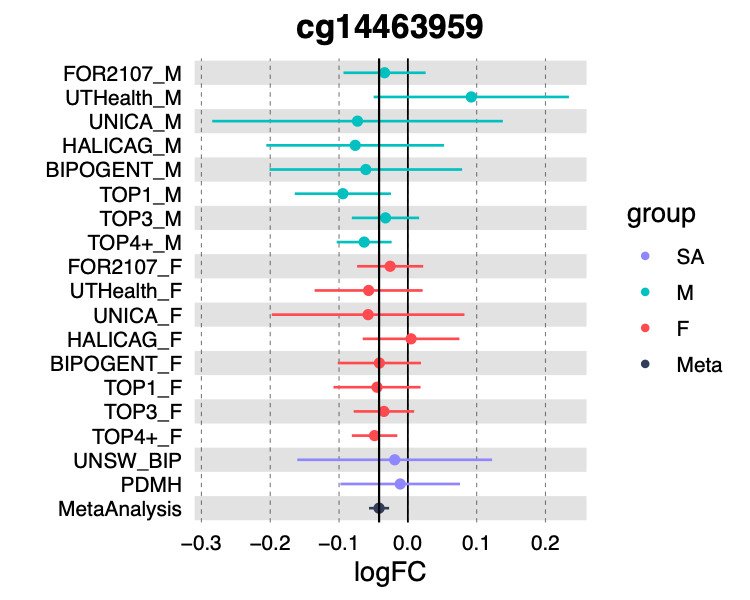 | 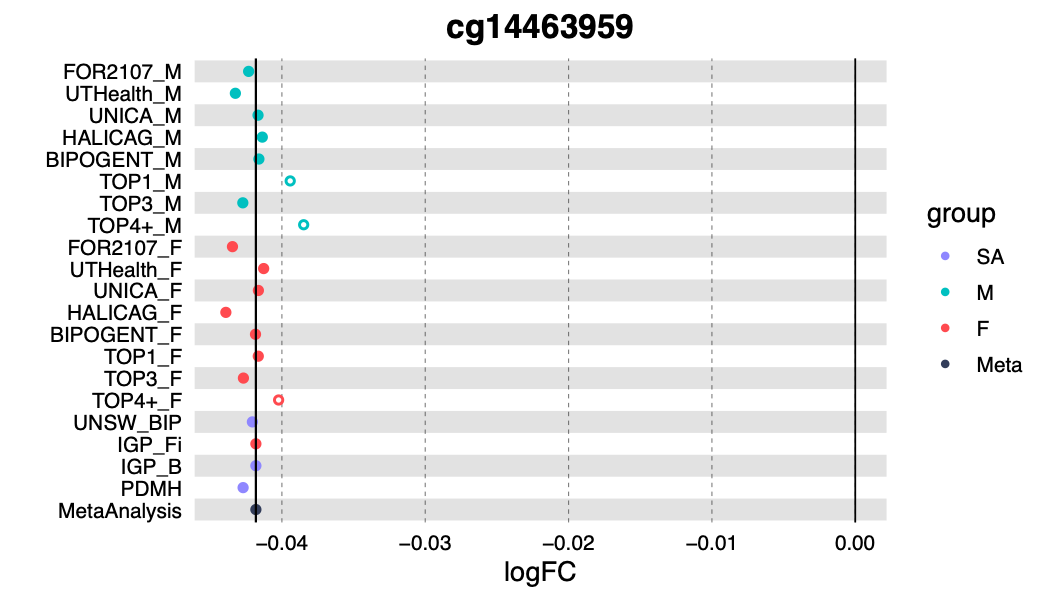 |
| 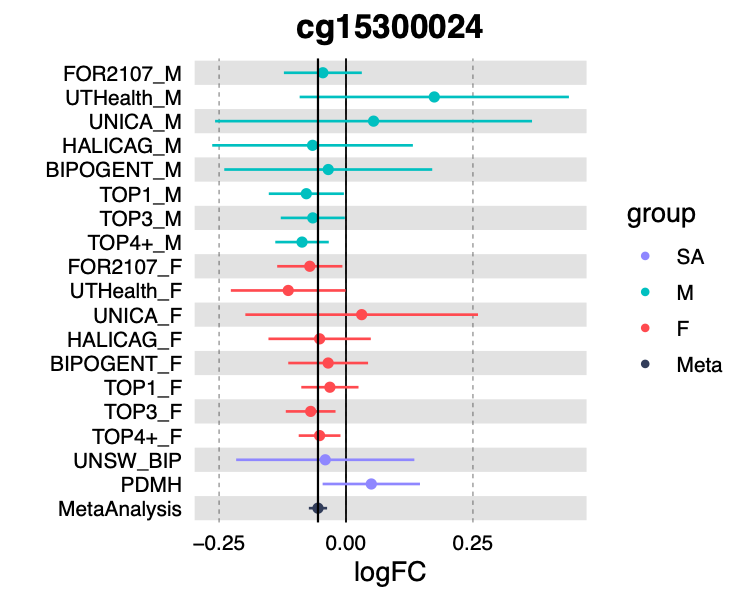 | 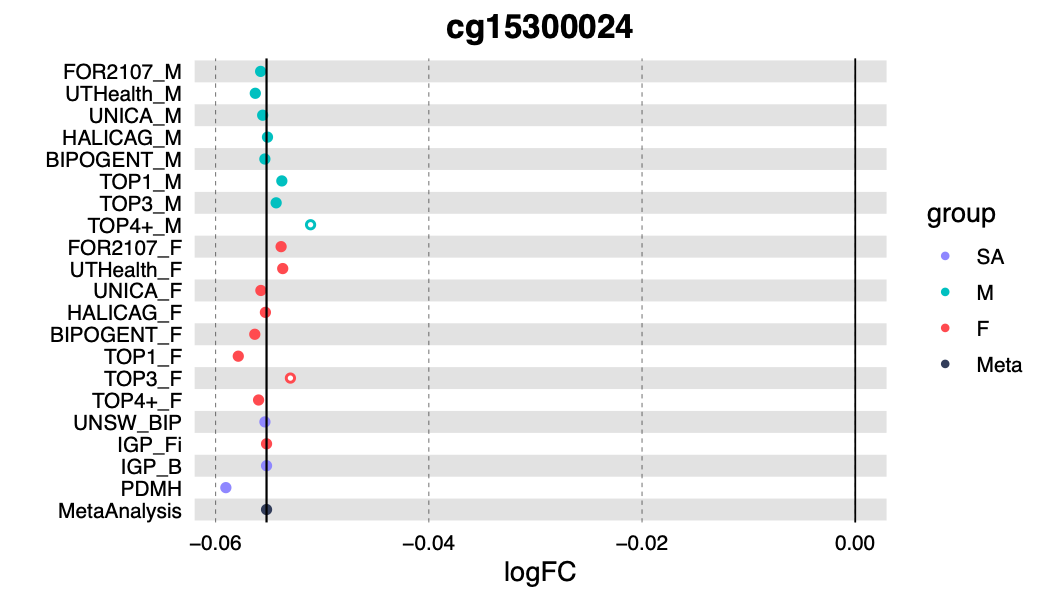 | 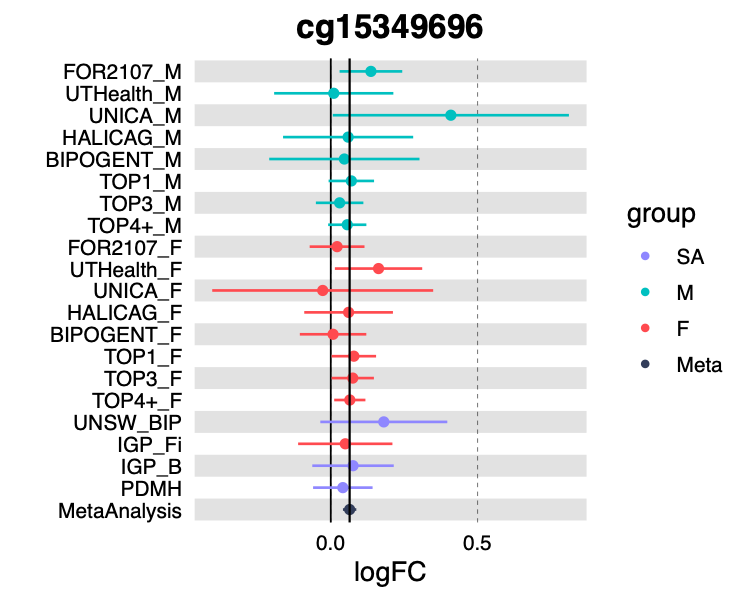 | 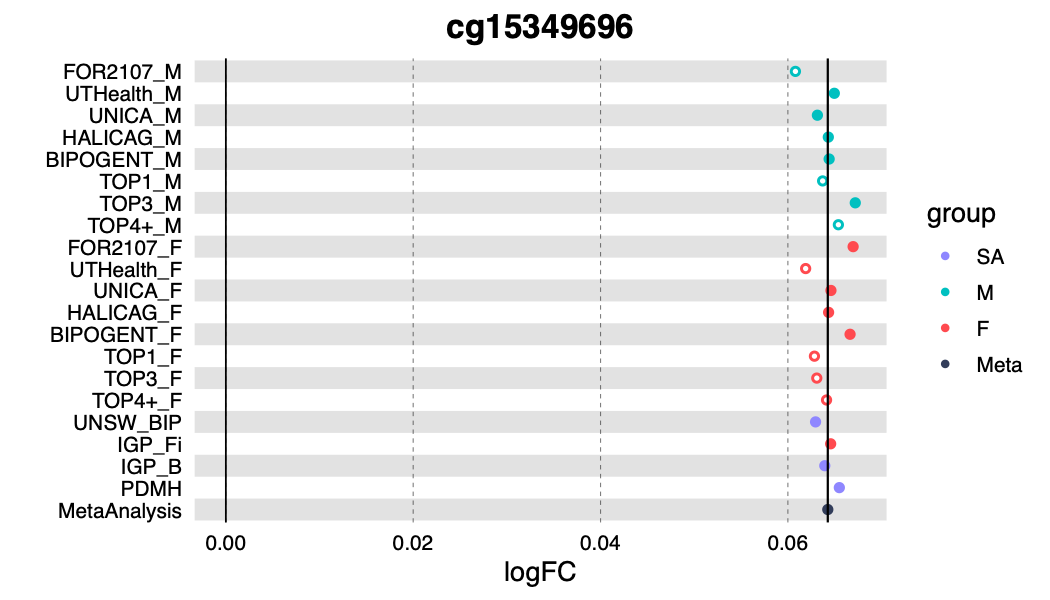 |
| 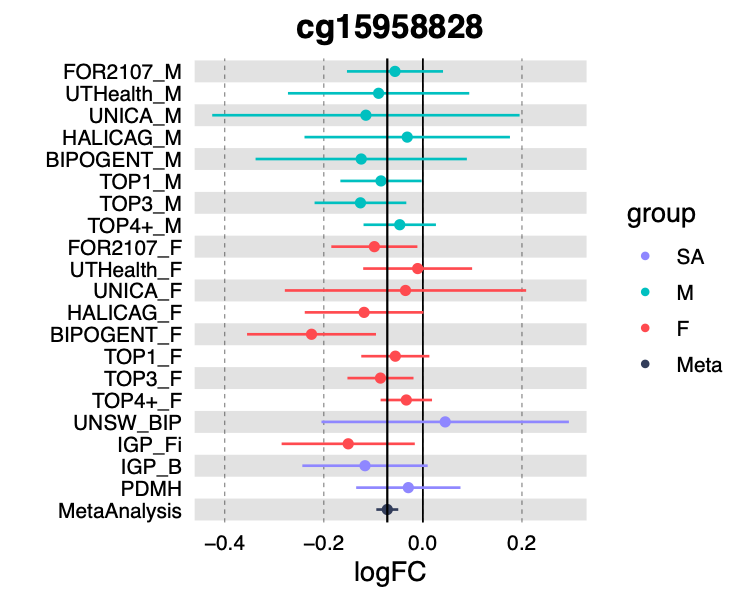 | 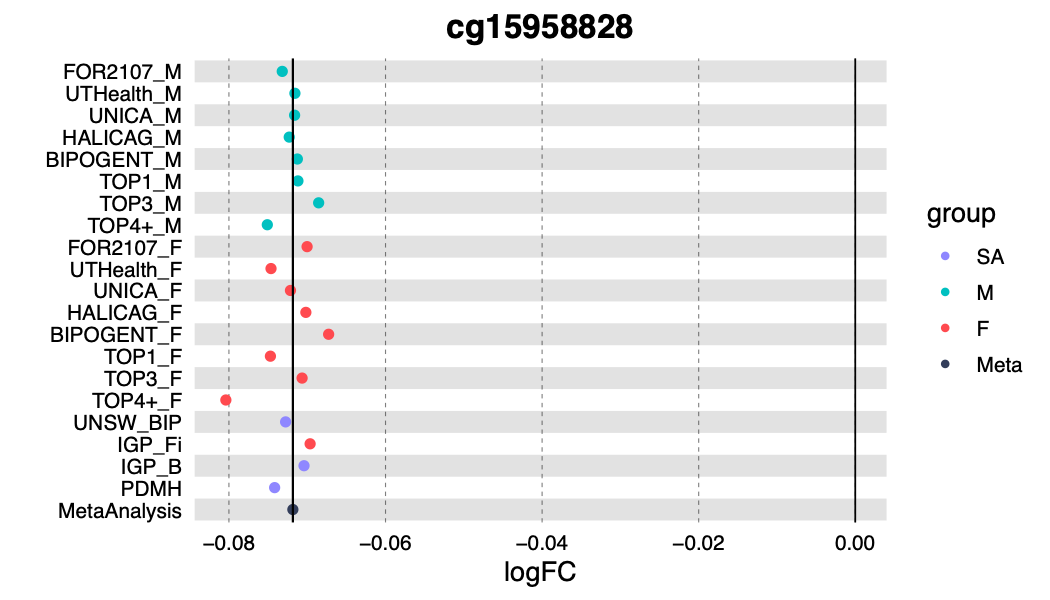 | 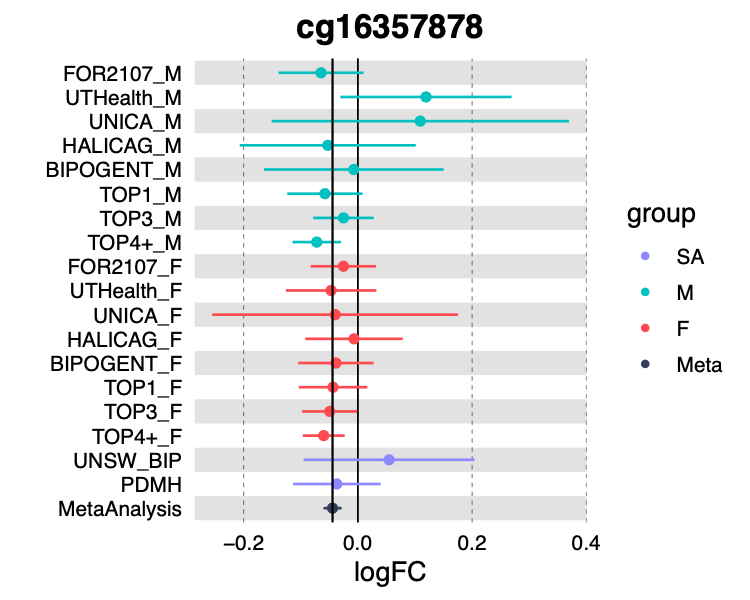 | 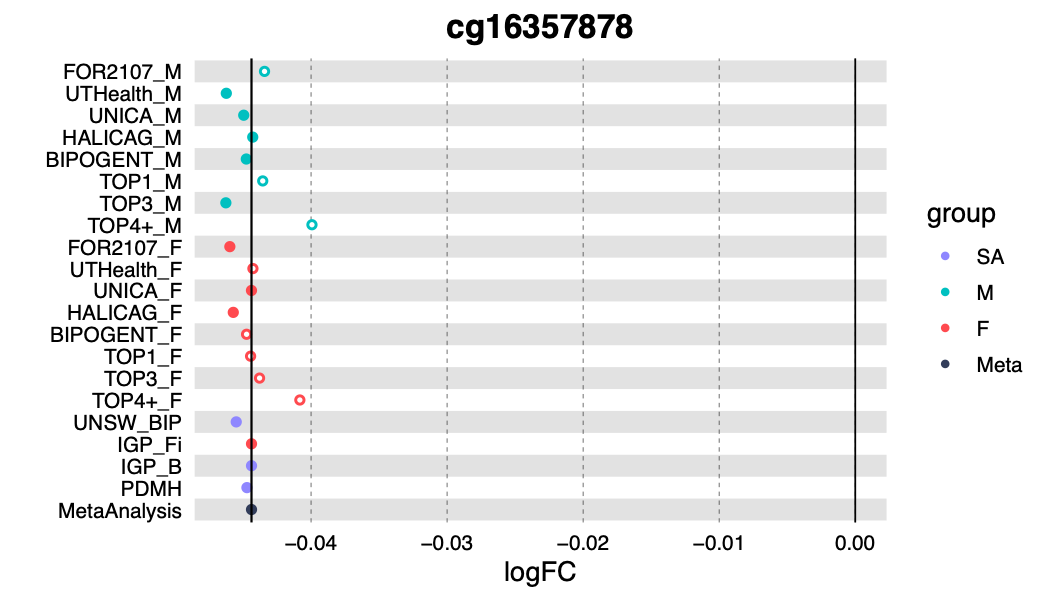 |
| 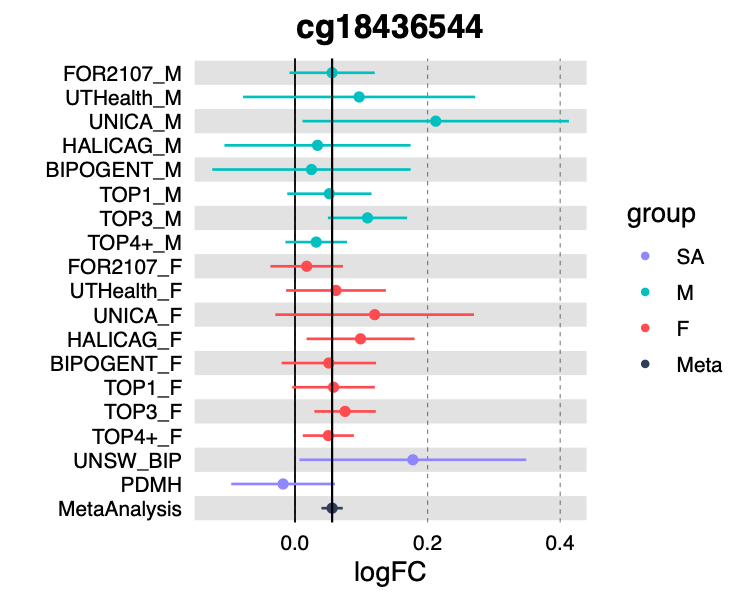 | 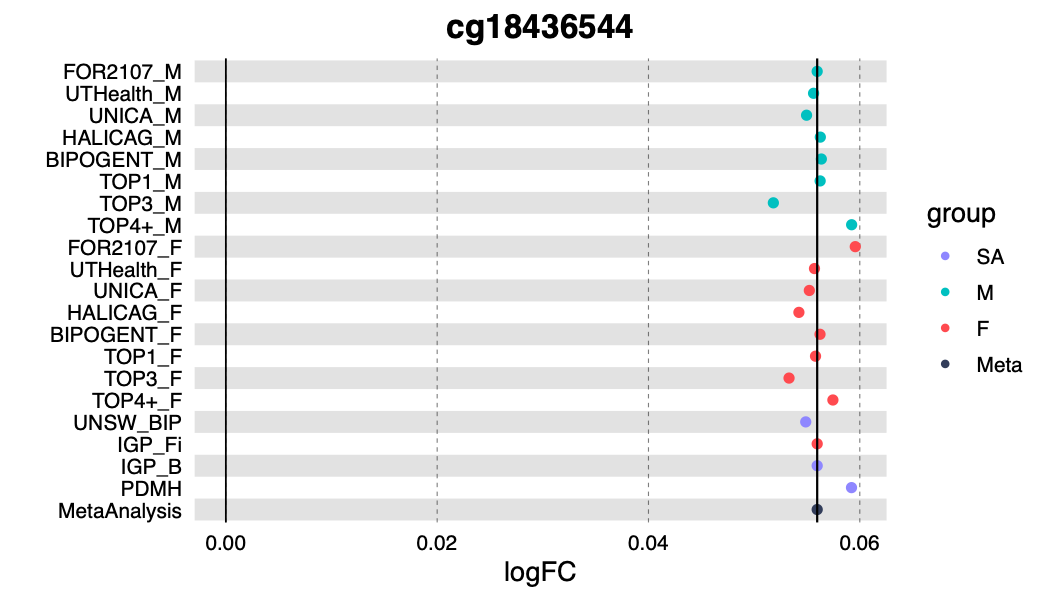 | 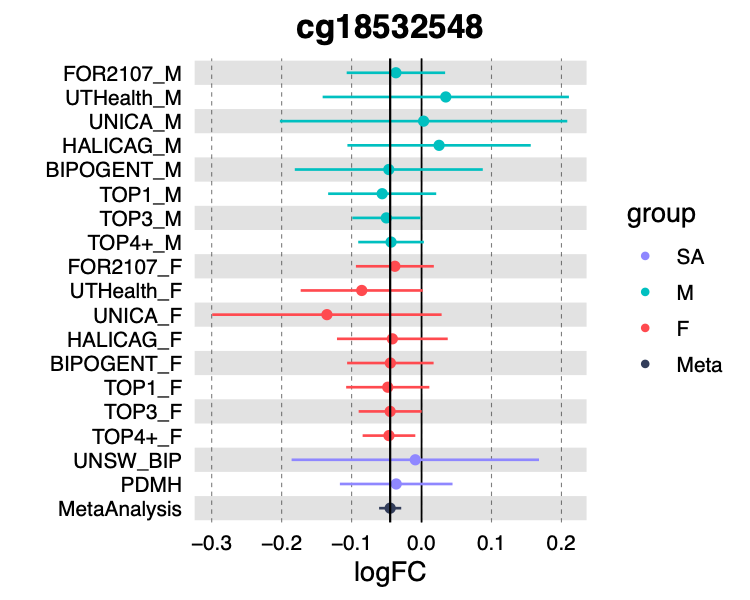 | 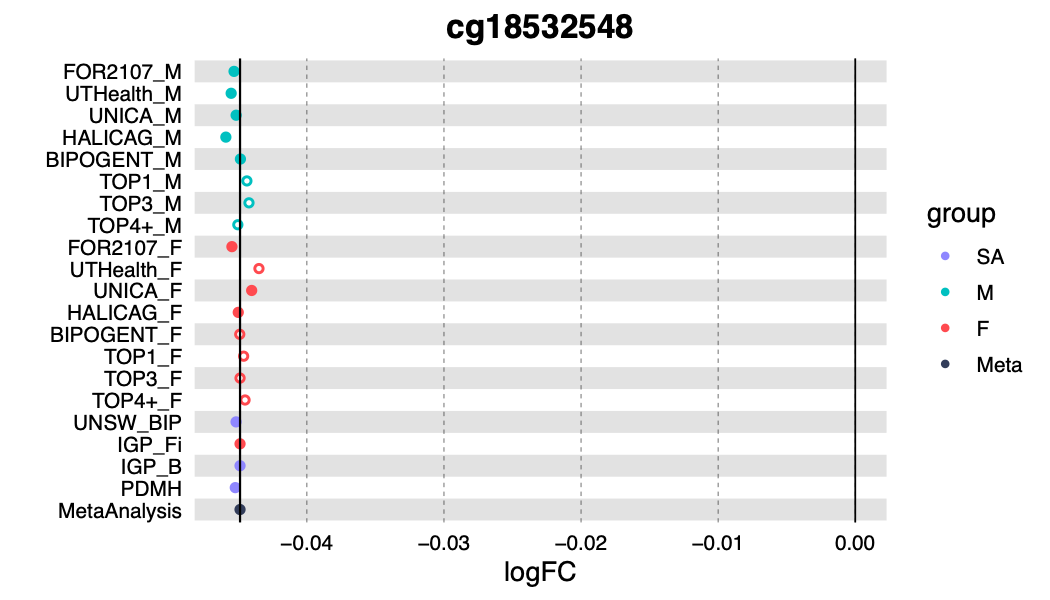 |
| 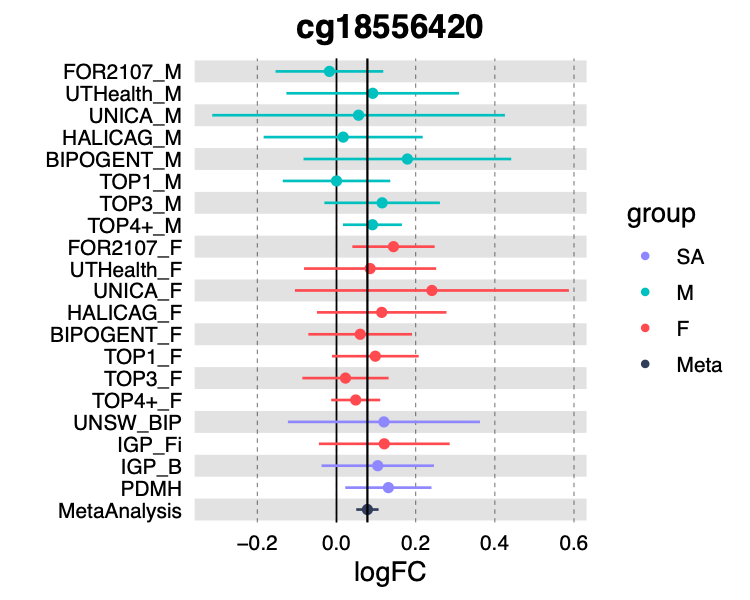 | 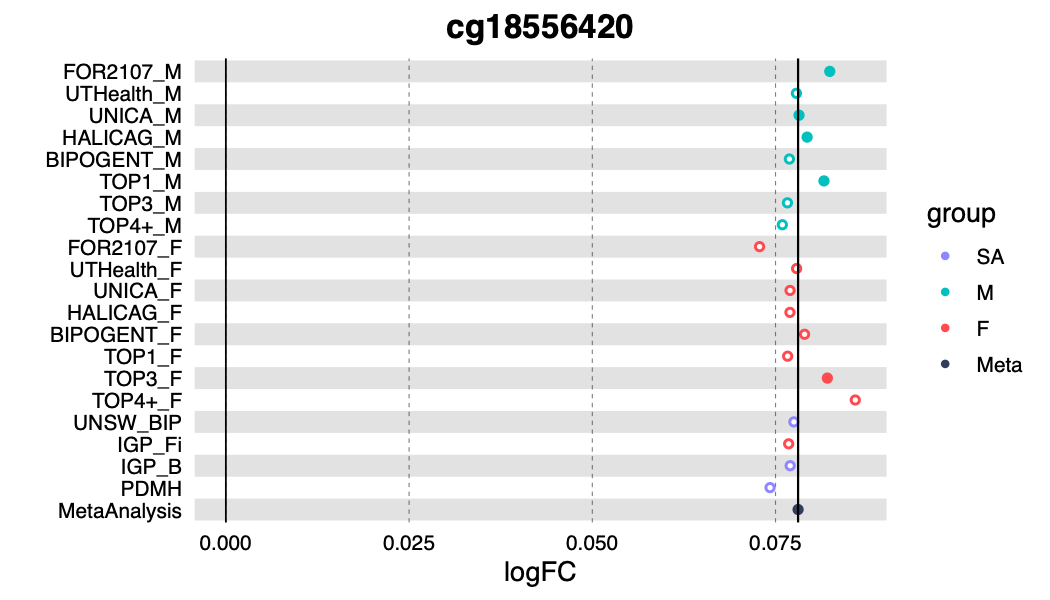 | 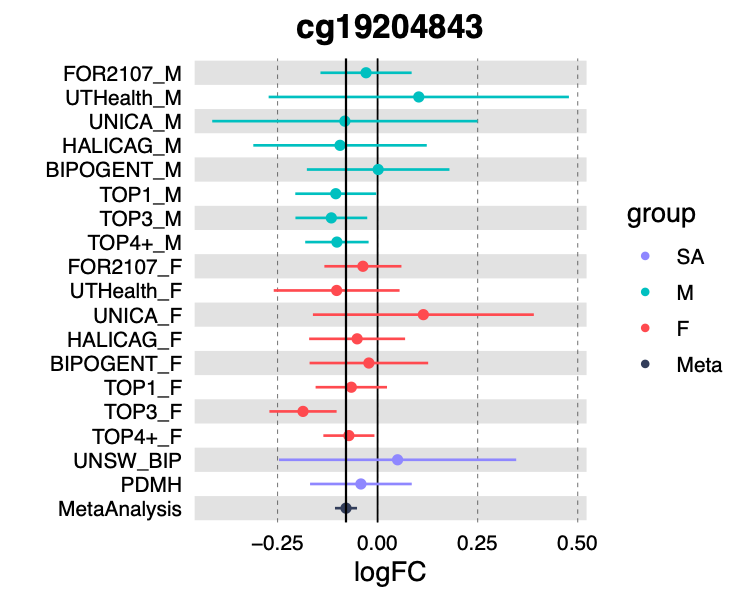 | 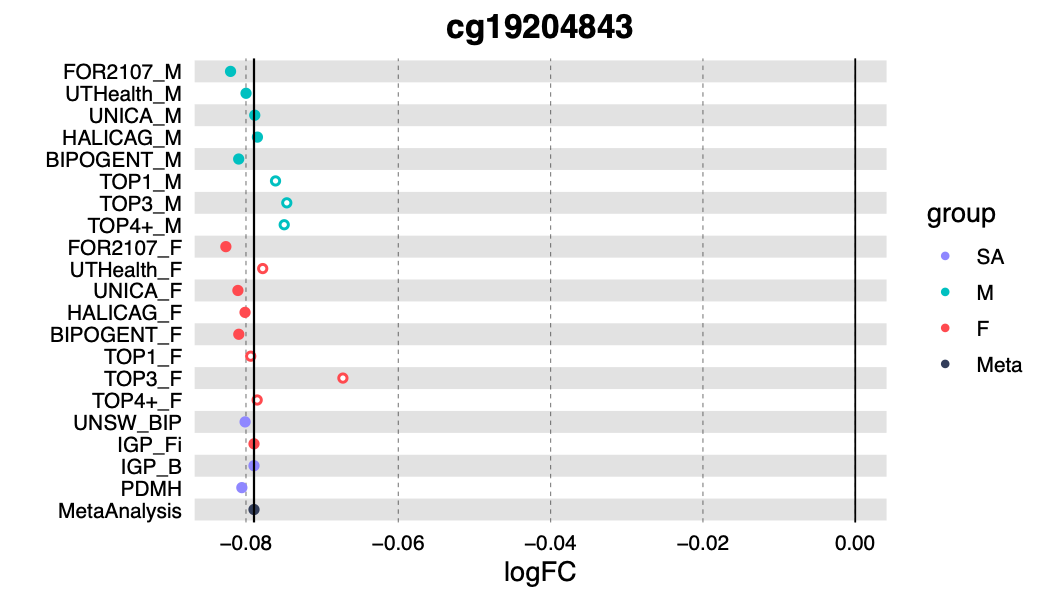 |
| 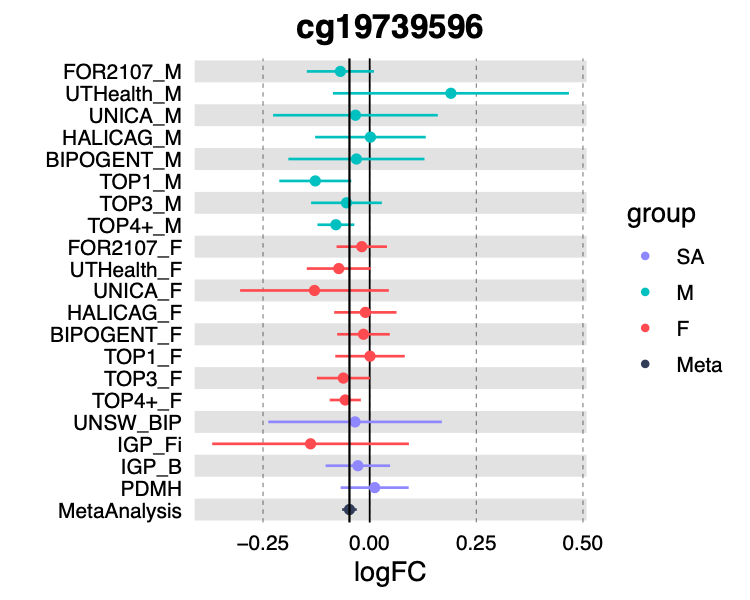 | 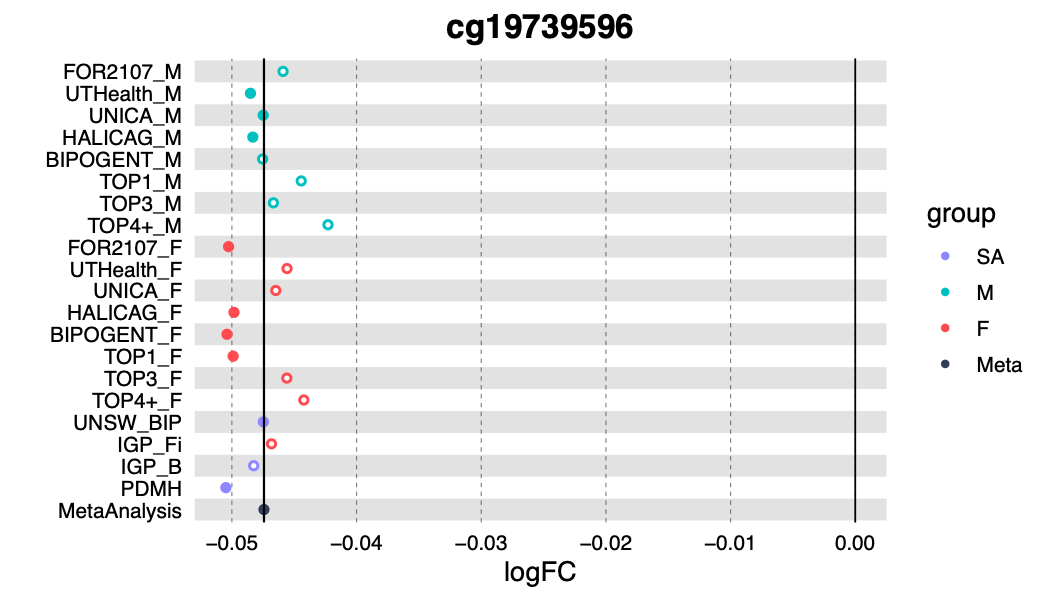 | 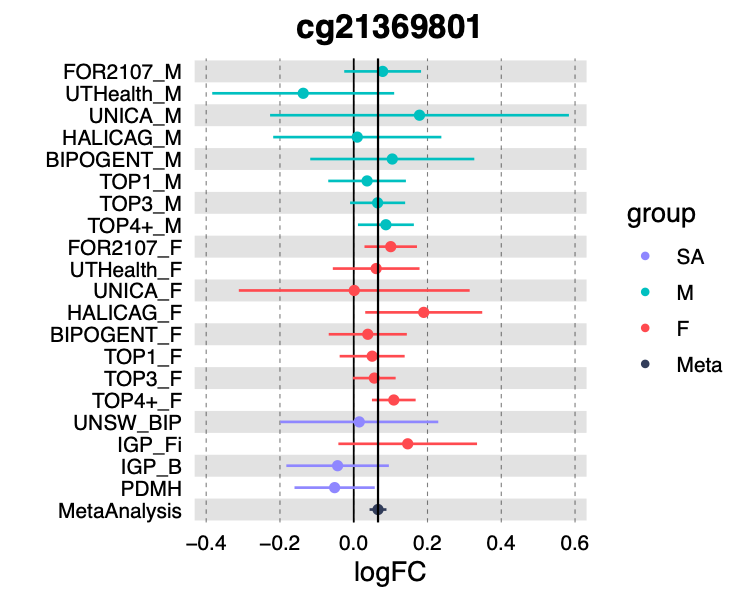 | 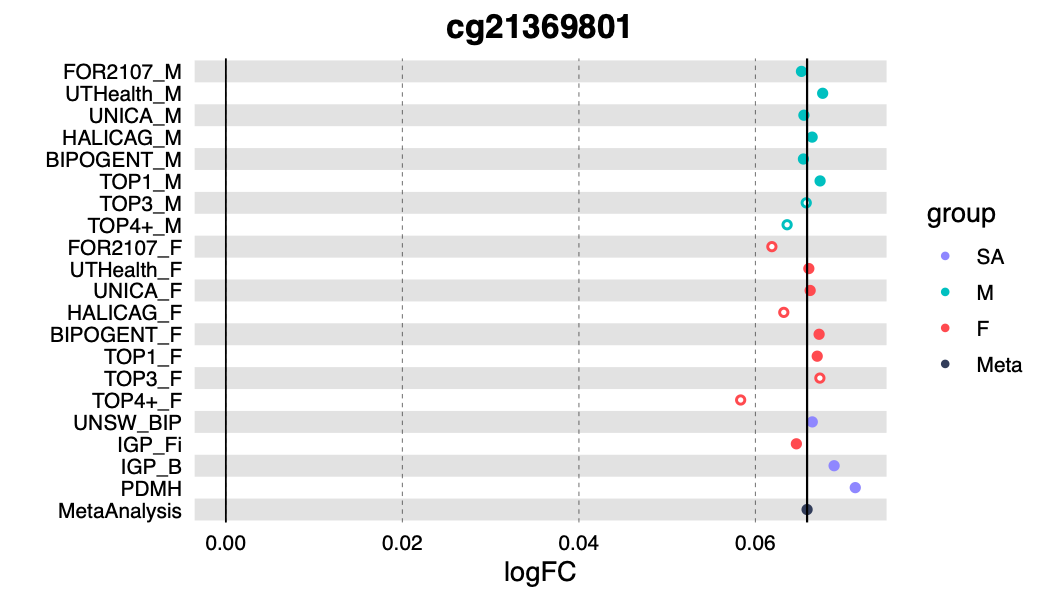 |
| 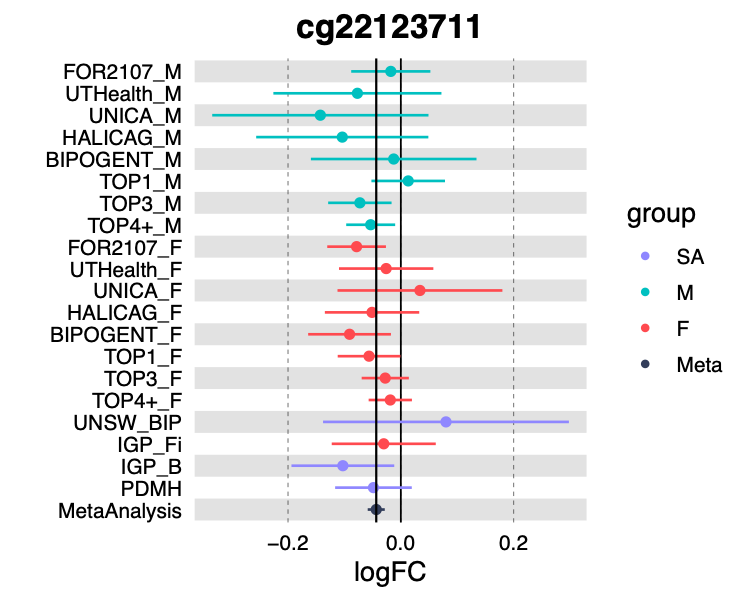 | 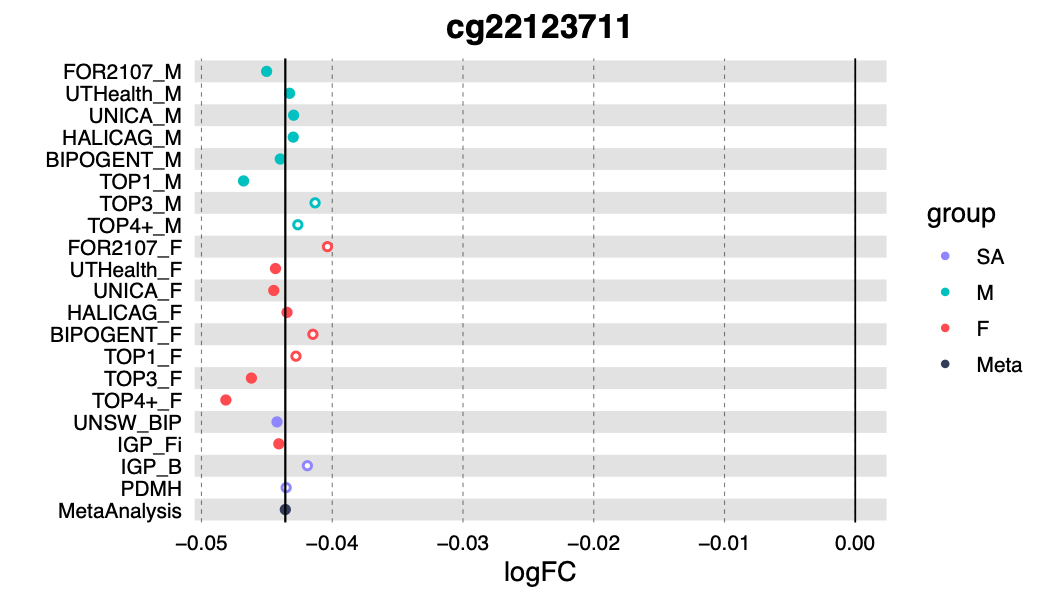 | 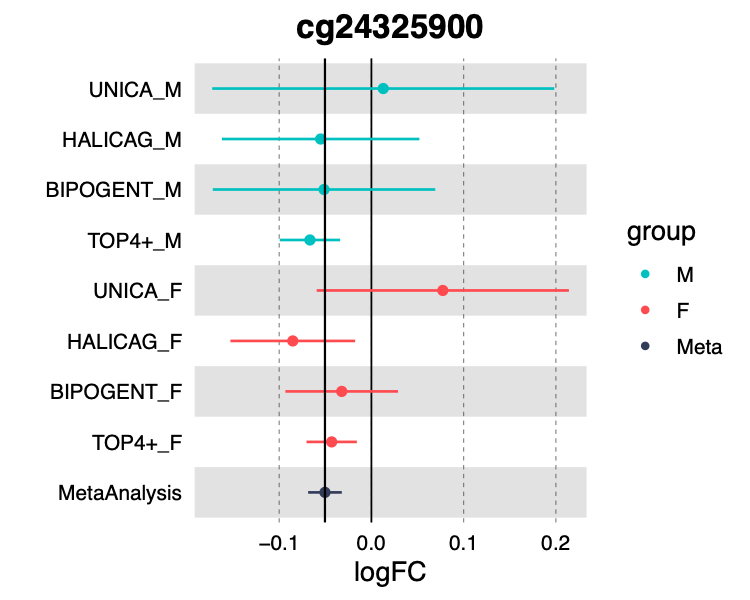 |  |
|  |  |  |  |
|  |  |  |  |
|  |  |  |  |
|  |  |  |  |
| B. | | | |
|  |  |  |  |
|  |  |  |  |

## Figure S5. GO term Revigo clusters (<http://revigo.irb.hr>) of enriched pathways for the total sample EWAS.^15^

A. Biological process clusters (part 1), B. Biological process clusters (part 2) C. Cellular component clusters, D. Molecular function clusters. Bubble color indicates the user-provided p-value; bubble size indicates the frequency of the GO term in the underlying GO database. Highly similar GO terms are linked by edges in the graph, where the line width indicates the degree of similarity.

## Blood-brain correlations

CpG Sites associated in meta-EWAS:

cg18436544, cg26689077, cg12836863, cg15958828, cg01538969, cg04418434, cg03460132, cg15300024, cg07687574, cg13240557, cg26720491, cg11222608, cg24410257, cg07869232, cg05508862, cg13241645, cg25247520, cg00565090, cg13549638, cg13947735, cg12753728, cg26538377, cg22123711, cg21369801, cg06980173, cg07249152, cg19204843, cg14463959, cg12116137, cg27663181, cg05380077, cg18532548, cg26262644, cg10519313, cg15349696, cg16357878, cg13876222, cg01909551, cg12022722, cg05244443, cg08606580, cg19739596

CpG sites which are not located on the 450k array and therefore cannot be checked

cg03460132, cg05244443, cg05380077, cg07249152, cg07687574, cg08606580, cg10519313, cg11222608, cg12022722, cg12753728, cg13240557, cg14463959, cg15300024, cg16357878, cg18436544, cg18532548, cg19204843, cg24410257, cg26262644, cg26538377, cg26720491, cg27663181

### Correlations in BECon database

Includes measurements from 16 individuals with samples taken from whole blood, Broadman Area 7, 10 and 20 and assessed using the Illumina 450k assay. The samples were collected post-mortem at the Douglas-Bell Canada Brain Bank. The subjects were aged between 15 and 87 years and included 4 females and 13 males. More information is published elsewhere.^16,17^

Spearman correlation was used to assess the correlation of CpG sites between blood and the brain areas using M-values. Variability represents the difference between the 10^th^ and 90^th^ percentile of CpG betas. Interesting sites were defined as sites with variability greater than 0.1 in the blood measurements and a correlation greater than 2x the standard deviation from the mean correlation of highly positively correlated polymorphic and sex chromosome CpG-enriched correlation peaks.

The results shown below were generated using the BeCon tool as implemented here: <https://redgar598.shinyapps.io/BECon/>

In all 19 sites were present in the database with cg13241645 (chr3, positive, ALAS1) and cg22123711 (chr1, negative, TNFRSF8) having the highest correlations. For more information see Supplementary Table (ST11).

In reference datasets with blood and brain methylation, four of the DMPs identified in sex-agnostic meta-analysis were significantly correlated between blood and brain regions in the Hannon et al. reference set (cg13876222 (*NOTCH1*) - prefrontal cortex; cg05508862 (*SLC5A10; FAM83G*)- entorhinal cortex; cg15349696 (*FOXK2*) and cg00565090 (*SNORD23*) - cerebellum (Supplementary file, and Supplementary Table S11 & S12). While in the Edgar et al. dataset (BECon), cg13241645 and cg22123711 showed the highest correlations. At the DMR level, we identified two CpGs (cg0482826 and cg05598727) with significant correlation between blood and brain methylation, both being located within the DMR chr5:8457425-8457857 annotated to *LINC02226; MIR4458HG.* Comparison of the genes annotated to the DMRs identified with those linked to BD in the prefrontal cortex^11^ showed that *RAB44*, *GET4, CLMN, PIK3R6, B3GNTL1*, and *ERG* overlapped between the two.

### Figure S6: CpG variability of the identified sites.

Figure generated from BECon.

### Figure S7: Correlation plots of the DMPs in relevant Brodmann regions

### Figure S8: Variance brain plot of the DMPs in relevant Brodmann regions

## Blood Brain Comparison Hannon et al. tool^18^

Includes measurements from 75 individuals with samples from whole blood as well as the entorhinal cortex, prefrontal cortex, superior temporal gyrus and cerebellum. Whole blood samples were obtained before death, while brain tissue was obtained post-mortem from the MRC London Neurodegenerative Disease Brain Bank and quantified using the 450k array. The subjects were aged between 70 and 99 years during blood sampling and 71 and 109 years at death and included 26 males and 49 females.^19^

Only sites were analyzed where the average difference between the 10^th^ and 90^th^ percentile was greater than 5%. Reported are the Pearson correlations in methylation values between the blood and brain areas. Correlations are significant if p < 0.05. In all, cg13876222 significantly correlated with the epigenetic signature of the prefrontal cortex (PFC), cg05508862 with the entorhinal cortex (EC), and cg15349696 as well as cg00565090, with the cerebellum (CER) (See Supplementary Tables: ST12)

None of the sites showed a low variation, defined as a DNA methylation range < 5%, in the blood sample and high variation, defined as a DNA methylation range > 5 % in the middle 80^th^ percentile of samples, in any of the brain samples, which would make the site ineligible as a blood-based proxy according to Hannon et al. (Figure S9-S12)

### Figure S9: cg13876222.

(Plot from: https://epigenetics.essex.ac.uk/bloodbrain/?probenameg=cg13876222)

### Figure S10: cg05508862.

(Plot from: https://epigenetics.essex.ac.uk/bloodbrain/?probenameg=cg05508862)

### Figure S11: cg15349696.

(Plot from https://epigenetics.essex.ac.uk/bloodbrain/?probenameg=cg15349696)

### Figure S12: cg00565090.

(Plot from https://epigenetics.essex.ac.uk/bloodbrain/?probenameg=cg00565090)

# Funding

**Acknowledgements**

GRo and MA are supported by CIHR (grant #166098), Genome Canada/Atlantic, ERA PerMed (PLOT-BD); AKS, and SLH are supported by RCN #250299, #273446, #223273; BC, LS are supported by ERA-NET (GEPI-BIOPSY - JTC2019), ANR JCJC (ANR-22-CE16-0029 - SchizoREmics), French government grant managed by the Agence Nationale de la Recherche under the France 2030 program (ANR-22-EXPR0013) and IReSP-INCa (SPAV1-22-018 - EPIPOLY); BJO, GR, JMF, PBM, PRS are supported by the Australian National Medical and Health Research Council (NHMRC) Program Grant #1037196 to Philip B. Mitchell, NHMRC Project Grants #1066177 and #1063960 to Janice M. Fullerton, NHMRC Investigator Grants #1176716 to Peter R. Schofield and #1177991 to Philip B. Mitchell, and NHMRC & Medical Research Futures Fund (MRFF) Grant #1200428 to Janice M. Fullerton. Janice M. Fullerton is the grateful recipient of the Janette Mary O’Neil Research Fellowship. Additional philanthropic support was provided by the Lansdowne Foundation, GoodTalk charity, the Gordon Pettigrew Family, Mrs Betty C. Lynch OAM (dec), and the Aberdeen Foundation; PRS is additionally supported by grant from MRFF (Australia). ES, EV, JL, LM are supported by Instituto de Salud Carlos III (grant ID PI15-00852 and PI18-00945), Brain and Behavior Research Foundation (grant ID 25811); JL is supported by an ERA-NET grant by the Instituto de Salud Carlos III and the European Regional Development Fund (ERDF) “A way to build Europe” (GEPI-BIOPSY; AC19/00129), and his group belongs to REIS (Red Temática Española de Investigación en Estrés), Ministerio de Ciencia e Innovación, REF. RED2022–134191-T); FS is supported by a 2023 NARSAD Young Investigator Grant (#31537) from the Brain & Behavior Research Foundation with support from the Families for Borderline Personality Disorder Research, and by the Hector Foundation II; GRF is supported by NIMH (K01MH121580), the John S. Dunn Foundation, and the Baszucki Group; IM is supported by Southern and Eastern Norway Regional Health Authority (Grant Numbers #2006233, #2006258, #2007004, #2011085, #2014102); JCB is supported by a Senior Research Career Scientist Award, VA Clinical Sciences Research and Development, lCX002767;KDH is supported by a University of Bergen PhD Grant; KJB is supported by a Career Development Award, VA Clinical Sciences Research and Development, IK2CX002694; KSO is supported by RCN #334920 and NIH5R01MH124839-02;MM is supported by ERA PerMed grant (PLOT-BD, JTC2018); MT is supported by RCN #273446, # 273291, and the National Institutes of Health grants R01MH125938; NAK and AAK are supported by a Research Career Scientist Award, VA Biomedical & Laboratory Research and Development Service, IK6BX006523; OAA is supported by RCN #324499, #324252, and Nordforsk #164218; TK is supported by consortia grants from the DFG FOR 2107 (grants FOR2107 KI588/14-1, KI588/14-2, KI588/20-1, KI588/22-1) and SFB/TRR 393 (project grant no 521379614); UD was funded by the German Research Foundation (DFG, grant FOR2107 DA1151/5-1, DA1151/5-2, DA1151/9-1, DA1151/10-1, DA1151/11-1 to UD; SFB/TRR 393, project grant no 521379614) and the Interdisciplinary Center for Clinical Research (IZKF) of the medical faculty of Münster (grant Dan3/016/26 to UD); AJF is supported by SFB/TRR 393 (project grant no 521379614). AT has been funded by the Deutsche Forschungsgemeinschaft (DFG, German Research Foundation) – 542489987. SM was funded by the Deutsche Forschungsgemeinschaft (DFG, German Research Foundation; Project-ID 521379614 – SFB/TRR 393, and ME62262-1), the Else Kröner-Fresenius-Stiftung (grant no. 2023_EKEA.153), and the Innovative Medical Research (IMF) of the Medical Faculty of the University of Münster (grant no. ME122205, ME122405). F. Stein was funded by the Deutsche Forschungsgemeinschaft (DFG, German Research Foundation) – grant STE3301/1-1 (project number 527712970) and Collaborative Research Centre/Transregio 393 (CRC/TRR 393, project number 521379614); and the Von Behring-Röntgen Society (project number 72_0013). OW reports funding support from the NHMRC, the Australian Government, the Edward C Dunn Foundation, the Australian Schizophrenia Research Bank, and the University of Queensland. TVL received research project support from the Research Council of Norway (RCN #288542) and South-East Health Authorities, Norway. The content is solely the responsibility of the authors and does not necessarily represent the official views of the funding agencies and the National Institutes of Health.

The Halifax-Cagliari and UNICA cohorts are supported by an ERA PerMed grant (PLOT-BD ERA PerMed JTC2018); the TOP cohort is supported by RCN #250299, #273446, #223273; the John S. Dunn Foundation and Pat Rutherford Chair in Psychiatry at UTHealth Houston supported the UTHealth Houston cohort. The FOR2107 cohort is part of the German multicentre consortium "Neurobiology of Affective Disorders. A translational perspective on brain structure and function", funded by the German Research Foundation (KI 588/14-1, KI 588/14-2, KI 588/22-1). FOR2107 is in part supported by DFG SFB/TRR 393 consortium, project number 521379614, and by the DYNAMIC initiative, which is funded by the LOEWE program of the Hessian Ministry of Science and Arts (Grant Number: LOEWE1/16/519/03/09.001(0009)/98). Biosamples and corresponding data were sampled, processed, and stored in the Marburg Biobank CBBMR.

# References

1. Garcia-Ruiz B, Jimenez E, Aranda S, et al. Associations of altered leukocyte DDR1 promoter methylation and childhood trauma with bipolar disorder and suicidal behavior in euthymic patients. *Mol Psychiatry* 2024; **29**(8): 2478–86.

2. Kircher T, Wohr M, Nenadic I, et al. Neurobiology of the major psychoses: a translational perspective on brain structure and function-the FOR2107 consortium. *Eur Arch Psychiatry Clin Neurosci* 2019; **269**(8): 949–62.

3. Hou L, Heilbronner U, Degenhardt F, et al. Genetic variants associated with response to lithium treatment in bipolar disorder: a genome-wide association study. *Lancet* 2016; **387**(10023): 1085–93.

4. Manchia M, Paribello P, Arzedi C, et al. A multidisciplinary approach to mental illness: do inflammation, telomere length and microbiota form a loop? A protocol for a cross-sectional study on the complex relationship between inflammation, telomere length, gut microbiota and psychiatric disorders. *BMJ Open* 2020; **10**(1): e032513.

5. Manchia M, Squassina A, Pisanu C, et al. Investigating the relationship between melatonin levels, melatonin system, microbiota composition and bipolar disorder psychopathology across the different phases of the disease. *Int J Bipolar Disord* 2019; **7**(1): 27.

6. O'Connell KS, Koromina M, van der Veen T, et al. Genomics yields biological and phenotypic insights into bipolar disorder. *Nature* 2025; **639**(8056): 968–75.

7. Watkeys OJ, Cohen-Woods S, Quide Y, et al. Derivation of poly-methylomic profile scores for schizophrenia. *Prog Neuropsychopharmacol Biol Psychiatry* 2020; **101**: 109925.

8. Loughland C, Draganic D, McCabe K, et al. Australian Schizophrenia Research Bank: a database of comprehensive clinical, endophenotypic and genetic data for aetiological studies of schizophrenia. *Aust N Z J Psychiatry* 2010; **44**(11): 1029–35.

9. Mitchell PB, Johnston AK, Corry J, Ball JR, Malhi GS. Characteristics of bipolar disorder in an Australian specialist outpatient clinic: comparison across large datasets. *Aust N Z J Psychiatry* 2009; **43**(2): 109–17.

10. Brancu M, Wagner HR, Morey RA, et al. The Post-Deployment Mental Health (PDMH) study and repository: A multi-site study of US Afghanistan and Iraq era veterans. *Int J Methods Psychiatr Res* 2017; **26**(3).

11. Djurovic S, Gustafsson O, Mattingsdal M, et al. A genome-wide association study of bipolar disorder in Norwegian individuals, followed by replication in Icelandic sample. *J Affect Disord* 2010; **126**(1-2): 312–6.

12. Hesam-Shariati S, Overs BJ, Roberts G, et al. Epigenetic signatures relating to disease-associated genotypic burden in familial risk of bipolar disorder. *Transl Psychiatry* 2022; **12**(1): 310.

13. Nurnberger JI, Jr., McInnis M, Reich W, et al. A high-risk study of bipolar disorder. Childhood clinical phenotypes as precursors of major mood disorders. *Arch Gen Psychiatry* 2011; **68**(10): 1012–20.

14. Mirza S, Lima CNC, Del Favero-Campbell A, et al. Blood epigenome-wide association studies of suicide attempt in adults with bipolar disorder. *Transl Psychiatry* 2024; **14**(1): 70.

15. Supek F, Bosnjak M, Skunca N, Smuc T. REVIGO summarizes and visualizes long lists of gene ontology terms. *PLoS One* 2011; **6**(7): e21800.

16. Edgar RD, Jones MJ, Meaney MJ, Turecki G, Kobor MS. BECon: a tool for interpreting DNA methylation findings from blood in the context of brain. *Transl Psychiatry* 2017; **7**(8): e1187.

17. Farre P, Jones MJ, Meaney MJ, Emberly E, Turecki G, Kobor MS. Concordant and discordant DNA methylation signatures of aging in human blood and brain. *Epigenetics Chromatin* 2015; **8**: 19.

18. Hannon E, Lunnon K, Schalkwyk L, Mill J. Interindividual methylomic variation across blood, cortex, and cerebellum: implications for epigenetic studies of neurological and neuropsychiatric phenotypes. *Epigenetics* 2015; **10**(11): 1024–32.

19. Lunnon K, Smith R, Hannon E, et al. Methylomic profiling implicates cortical deregulation of ANK1 in Alzheimer's disease. *Nat Neurosci* 2014; **17**(9): 1164–70.
