# Supplementary material for: DNA methylation signatures associated with bipolar disorder in peripheral blood improve prediction models
Source: eBioMedicine. 2026 May 8;128:106284. doi: 10.1016/j.ebiom.2026.106284 (PMC13187609; doi:10.1016/j.ebiom.2026.106284)
Supplement: Workgroup table PUBMED [file mmc3.docx]

| **First name** | **Last name** |
| --- | --- |
| Jean | Beckham |
| Patrick | Calhoun |
| Eric | Dedert |
| Eric | Elbogen |
| Hurley | Robin |
| Jason | Kilts |
| Nathan | Kimbrel |
| Angela | Kirby |
| Anna | Magnante |
| Sarah | Martindale |
| Christine | Marx |
| Scott | McDonald |
| Scott | Moore |
| Victoria | O'Connor |
| Rajendra | Morey |
| Jennifer | Naylor |
| Jared | Rowland |
| Robert | Shura |
| Cindy | Swinkels |
| Ryan | Wagner |
